# Epidemiological trends and disparities in iodine, vitamin A, and iron deficiencies among children aged 0–14 years globally, 1990–2021

**DOI:** 10.3389/fnut.2025.1622945

**Published:** 2025-12-10

**Authors:** Fang Peng, Xiaojuan Lin, Shanshan Liu, Hui Zou, Canhong Yi, Yuquan Tian

**Affiliations:** Hunan Provincial Key Laboratory of Pediatric Respirology, Pediatric Medical Center, Hunan Provincial People’s Hospital (the First Affiliated Hospital of Hunan Normal University), Changsha, China

**Keywords:** nutritional deficiencies, child health, global burden of disease, vitamin A deficiency, iron deficiency, socio-demographic index (SDI), iodine deficiency

## Abstract

**Background:**

Nutritional deficiencies in iodine, vitamin A, and iron remain major public health challenges for children under 15 years, impairing growth, cognitive development, and long-term health outcomes. Despite global interventions, disparities persist across socioeconomic strata. This study evaluates trends, regional variations, and sociodemographic correlates of these deficiencies from 1990 to 2021.

**Methods:**

Data from the Global Burden of Disease 2021 database were analyzed for 204 countries, focusing on children aged 0–14 years. Age-standardized incidence rate, DALYs, and SDI correlations were assessed using Expected Annual Percentage Change (EAPC) and Bayesian age-period-cohort models to project trends to 2050.

**Results:**

From 1990 to 2021, iodine deficiency incidence rate declined by 92.9% (EAPC: −0.04), with DALY rates dropping 98.7%. Vitamin A deficiency incidence rate decreased by 94.9% (EAPC: −2.02), while DALY rates fell 92.8%. Dietary iron deficiency DALY rates remained stable (EAPC: 0.53). Low SDI regions exhibited 7.3 × higher iodine and 151 × higher vitamin A deficiency DALY rates than high SDI regions. Projections indicate sustained declines for iodine and vitamin A deficiencies but stagnation in iron deficiency, particularly in conflict-affected areas.

**Conclusion:**

While iodine and vitamin A deficiencies have significantly reduced globally, dietary iron deficiency persists in low SDI regions. Tailored interventions, including fortification programs and health system strengthening, are critical to addressing inequities and achieving global nutrition targets.

## 1 Introduction

Millions of children globally are affected by nutritional deficiencies, including iodine deficiency, vitamin A deficiency, and dietary iron deficiency. These preventable public health issues pose continuous threats to the survival quality and developmental prospects of children aged 0–14 years. Iodine deficiency may result in goitre, cognitive and physical development delays in children aged 0–14 years, hypothyroidism, and heightened mortality in newborns (1). Vitamin A deficiency elevates susceptibility to diarrhea, measles, lower respiratory tract infections, and can induce immune deficiencies, growth abnormalities, and anemia (2, 3). Dietary iron deficiency primarily presents as iron-deficiency anemia, which compromises cognitive function, motor skills, immune regulation, and other nutrient metabolisms (4). These micronutrient deficiencies not only cause growth failure, compromised immunity, and cognitive development disorders during childhood (5), but they also have long-term adverse effects in adulthood, elevating the likelihood of chronic ailments and diminishing societal productivity.

Children, due to their distinct physiological characteristics and nutritional needs, are particularly vulnerable to nutritional deficiencies, a risk further exacerbated by immature digestive and absorption functions, limited dietary diversity, and high exposure to infectious diseases (6–9). Prior research has predominantly centered on cross-sectional analyses at specific points in time, which may not adequately capture the evolving epidemiological characteristics of global nutritional deficiencies from 1990 to 2021. This is particularly evident when considering the marked disparities in intervention effectiveness and dietary transitions between regions with a low socio-demographic development index (SDI) and those with medium and high SDI (10).

Recent UNICEF and WHO global nutrition reports (2023–2024) have similarly documented substantial reductions in iodine and vitamin A deficiencies among children worldwide, consistent with the downward trends identified in our analysis. However, both organizations also highlight persistent iron deficiency and anemia as ongoing challenges, particularly in low- and middle-income countries, which aligns with our findings on the stagnation of dietary iron deficiency.

In contrast, the majority of existing research has incorporated data on childhood nutritional deficiencies within a broad population-based analytical framework, without specific focus on this age group. This has led to skewed estimations of disease burden in high-risk subgroups, particularly among children under the age of five (10). These data gaps significantly impede policy makers’ ability to accurately assess the priority of child nutrition interventions. For instance, areas with low SDI should prioritize the provision of basic nutrients, while areas with medium and high SDI need to be aware of the potential risk of hidden hunger due to dietary refinement (11).

This study utilizes data from the Global Burden of Disease (GBD) 2021 to conduct a systematic analysis of the epidemiological status of iodine deficiency, vitamin A deficiency, and dietary iron deficiency among children aged 0–14 years in 2021. It quantifies the spatial distribution characteristics of their incidence rates and disability-adjusted life-years (DALYs), and assesses the trend of the burden of disease related to these three nutritional deficiencies over the period 1990–2021. This evaluation aims to reveal the effectiveness of long-term interventions as well as identify potential challenges. Furthermore, by integrating the SDI classification, the study compares the differences in disease burden among regions and countries at varying levels of development. The age-specific analyses based on global data on the burden of disease not only illuminate the spatial and temporal evolution of nutritional deficiencies in children but also lay a critical foundation for the development of a staged and differentiated intervention strategy (12).

## 2 Methods

### 2.1 Data sources

This study utilizes data from the GBD 2021 database, which collects and analyzes information on nutritional deficiencies among children aged 0–14 years across 204 countries and territories worldwide from 1990 to 2021. It encompasses key indicators such as morbidity, DALY rates, and SDI. The GBD dataset amalgamates information from a variety of sources, including surveillance data reported by national health authorities, population health surveys (e.g., WHO Global Health Observatory), systematic reviews of scientific literature, and various modeling estimates. This integration ensures both spatial and temporal continuity of the data, as well as its comparability. For further details, please visit the data source at: https://ghdx.healthdata.org/gbd-results-tool. This study was approved by the First Affiliated Hospital of Hunan Normal University. This study employed publicly available data that did not include confidential or personally identifiable patient information.

The SDI is a composite, multidimensional evaluation system that quantitatively assesses the stage of social development (13). It integrates three core dimensions: regional economic output capacity, population quality base, and demographic characteristics. These characteristics include parameters such as per capita economic income, the number of years of schooling for groups aged 15 years and above, and the total fertility rate. The computational model of the SDI employs a standardized algorithm to map the results to a continuous interval of 0–1. In this model, a value closer to 1 indicates a higher maturity of socio-demographic development. The gradient is divided into five tiers (low, medium-low, medium, medium-high, and high-development), thereby providing a standardized benchmarking framework for the analysis of global health disparities and policy formulation.

### 2.2 Definition of cases

Nutritional deficiencies represent a primary cause for the non-fatal estimation of several subcauses, including: (1) vitamin A deficiency, (2) iodine deficiency, (3) dietary iron deficiency, (4) protein-energy malnutrition, and (5) other nutritional deficiencies. These five subcauses are modelled separately, with distinct case definitions, input data, strategies, and severity distribution analyses. Consequently, we discuss each subcause in sequence.

Vitamin A deficiency is a condition stemming from insufficient dietary intake or bioavailability of vitamin A to meet physiological requirements. It is marked by reduced serum or breast milk retinol concentrations, or the presence of clinical symptoms such as night blindness, xerophthalmia, and retinitis. In the GBD 2021, a case of vitamin A deficiency is defined by a serum retinol concentration of less than 0.7 μmol/L.

Iodine deficiency is characterized by impaired thyroid hormone production due to insufficient iodine intake, leading to a range of adverse health outcomes from thyroid gland enlargement (goitre) to severe physical and intellectual disability. Our assessment of the non-fatal burden of iodine deficiency includes estimates of only the subset associated with visible goitre (grade 2) and its subsequent effects, such as thyroid dysfunction, heart failure, and intellectual disability (historically referred to as cretinism). However, it does not incorporate estimates of sub-clinical iodine deficiency or non-visible goitre (e.g., the absence of a visible goitre) induced by iodine deficiency.

In the GBD cause analysis, dietary iron deficiency is characterized as mild, moderate, or severe anemia stemming from insufficient dietary iron intake. This deficiency is not attributed to other factors that compromise the absolute or functional availability of iron needed by the body.

Nutritional deficiencies D50–D53. 9, E00–E02, E40–E46. 9, E50–E61. 9, E63–E64. 9 were classified according to the International Classification of Diseases and Injuries, 10th edition (ICD-10). These include iodine deficiency (coded as E00–E02), dietary iron deficiency (D50–D53. 9), and vitamin A deficiency (E50–E61. 9, E64. 1) (14).

### 2.3 Statistical methods

The Expected Annual Percentage Change (EAPC) is a regression modeling-based tool used in epidemiological analysis. It is designed to evaluate the average annual relative change in a specific health indicator (such as disease incidence, mortality, or burden of disease indicator) over time (15). The fundamental principle involves transforming the natural logarithm of the indicator and then linearly fitting it to the time variable, as calculated by the formula defined as:


$$\text{EAPC}=(e^{\alpha }-1)\times 100$$

The slope coefficient *α* represents the time variable in the regression analysis, while *e* denotes a natural constant, approximately 2.71828. By utilizing a log-linear model, researchers can scrutinize both the direction (positive or negative) and intensity of an indicator trend. To bolster the reliability of the findings, estimates of the EAPC and its 95% confidence interval (95% CI) are commonly computed using the R language (version 4.21).

Bayesian age-period-cohort modeling, a statistical tool employed to analyze and predict shifts in demographic data, has gained widespread use in the forecasting of epidemiological trends for various diseases (16). Utilizing this model, we projected the number of incidence cases and DALYs for nutritional deficiency subtypes among children aged 0–14 from 2022 to 2050. This projection aims to elucidate the future trajectory of the burden associated with these nutritional deficiencies in children within the specified age range. Prior to analysis, raw GBD 2021 data were screened for missing or inconsistent values. Outliers were cross-checked against WHO and national health survey data. The EAPC and Bayesian models were validated by examining residual diagnostics and performing sensitivity analyses using alternative prior distributions to ensure robustness and reproducibility.

## 3 Results

### 3.1 Global trends in different subtypes of nutritional deficiencies, 0–14 years, 2021

**Figure 1:**
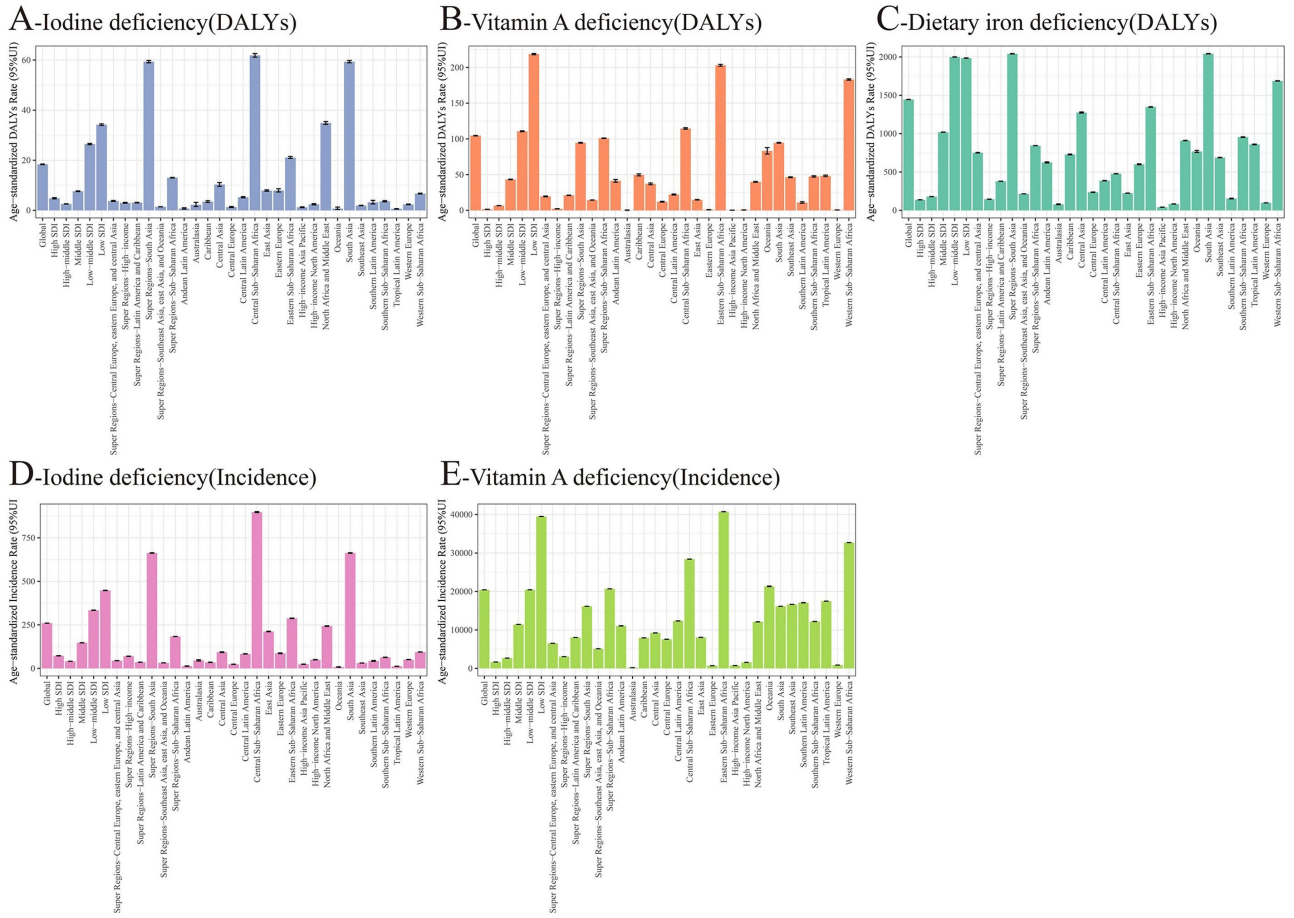
Age-standardized incidence and DALY rates of nutritional deficiencies among children aged 0–14 years at the global and regional levels in 2021. **(A)** Age-standardized DALY rate of iodine deficiency (per 100,000 population). **(B)** Age-standardized DALY rate of vitamin A deficiency (per 100,000 population). **(C)** Age-standardized DALY rate of dietary iron deficiency (per 100,000 population). **(D)** Age-standardized incidence rate of iodine deficiency (per 100,000 population). **(E)** Age-standardized incidence rate of vitamin A deficiency (per 100,000 population). *X*-axis = region; *Y*-axis = rate (per 100,000 population). Error bars represent 95% uncertainty intervals where available. SDI, sociodemographic index; DALY, disability-adjusted life year.

**Figure 2:**
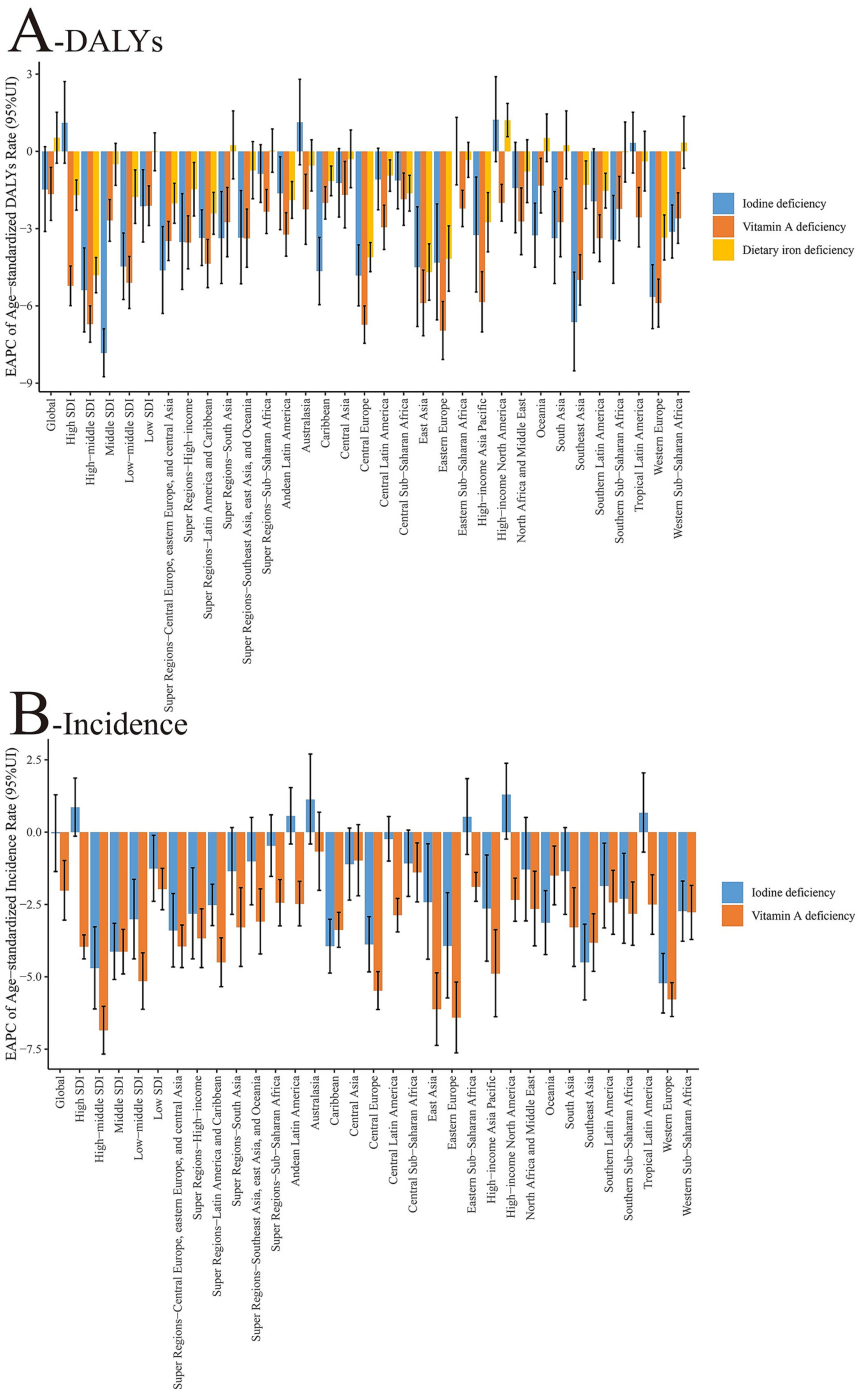
Changing trends of age-standardized incidence and DALY rates of nutritional deficiencies among children aged 0–14 years at the global and regional levels from 1990 to 2021. **(A)** Age-standardized DALY rate (per 100,000 population). **(B)** Age-standardized incidence rate (per 100,000 population). *X*-axis = year (1990–2021); *Y*-axis = rate (per 100,000 population). Shaded areas or error bars indicate 95% uncertainty intervals where applicable. SDI, sociodemographic index; DALY, disability-adjusted life year.

According to the 2021 GBD data, there are significant differences in the epidemiological characteristics of nutritional deficiency subtypes among children aged 0–14 years. With regards to incidence rate, the global age-standardized incidence rate of iodine deficiency was 18.36/100,000 in 2021, marking an approximate 92.9% decrease from 259.76/100,000 in 1990. Additionally, the incidence rate of vitamin A deficiency declined by about 94.9%, from 20426.14/100,000 in 1990 to 10,450/100,000 in 2021. In terms of DALY rates, iodine deficiency saw a reduction from 1445.61/100,000 in 1990 to 18.36/100,000 in 2021 (a 98.7% decrease), while the DALY rate for vitamin A deficiency decreased from 1445.61/100,000 to 1045.50/100,000 (a 92.8% decrease). The global age-standardized DALY rate for dietary iron deficiency was 1480.99/100,000 in 2021, reflecting a stable trend compared with 1990, and clearly indicating that this measure refers to age-standardized DALYs per 100,000 population. Time trend analysis revealed that the EAPC in the incidence rate of iodine deficiency from 1990–2021 was −0.04 (95% CI: −0.07 to −0.01), indicating a modest but statistically significant decreasing trend. For vitamin A deficiency, the EAPC was −2.02, denoting a significant downward trend. Regarding changes in the DALY rate, the EAPC for iodine deficiency was −1.48, for vitamin A deficiency was −1.66, and for dietary iron deficiency was 0.53, suggesting no substantial improvement in their burden (Tables 1, 2 and Figures 1, 2).

**Table 1:** Age-standardized incidence and DALYs rates of nutritional deficiency in 0–14 years at the global and regional levels in 2021.

Location	Iodine deficiency	Vitamin A deficiency	Dietary iron deficiency
DALYs (disability-adjusted life years)			
Global	18.36 (18.24, 18.48)	104.50 (104.22, 104.79)	1445.61 (1444.52, 1446.71)
High-middle SDI	2.57 (2.50, 2.63)	6.68 (6.56, 6.79)	179.55 (178.96, 180.14)
High SDI	4.82 (4.59, 5.06)	1.47 (1.36, 1.58)	137.88 (136.82, 138.94)
Low-middle SDI	26.47 (26.22, 26.72)	110.69 (110.15, 111.24)	1999.55 (1997.21, 2001.89)
Low SDI	34.20 (33.89, 34.51)	218.63 (217.84, 219.42)	1985.09 (1982.67, 1987.52)
Middle SDI	7.63 (7.48, 7.78)	43.28 (42.90, 43.67)	1019.26 (1017.39, 1021.12)
Central Europe, Eastern Europe, and Central Asia	3.75 (3.57, 3.94)	19.52 (18.97, 20.09)	751.65 (748.20, 755.11)
High-income	3.01 (2.85, 3.19)	2.23 (2.09, 2.39)	145.66 (144.45, 146.88)
Latin America and Caribbean	3.09 (2.98, 3.20)	21.08 (20.83, 21.34)	379.02 (377.92, 380.12)
Southeast Asia, East Asia, and Oceania	1.43 (1.40, 1.47)	14.42 (14.30, 14.54)	215.49 (215.03, 215.96)
Sub-Saharan Africa	13.04 (12.94, 13.15)	100.93 (100.63, 101.23)	844.04 (843.18, 844.91)
North Africa and Middle East	34.89 (34.33, 35.46)	39.89 (39.38, 40.41)	909.81 (907.28, 912.34)
South Asia	59.36 (58.89, 59.83)	94.58 (94.07, 95.09)	2041.71 (2039.25, 2044.17)
Andean Latin America	0.73 (0.51, 1.02)	41.29 (39.39, 43.26)	625.01 (617.70, 632.38)
Australasia	2.24 (1.55, 3.18)	0.11 (0.01, 0.53)	78.75 (74.08, 83.67)
Caribbean	3.51 (3.18, 3.87)	49.64 (48.28, 51.03)	728.70 (723.50, 733.92)
Central Asia	10.32 (9.59, 11.09)	37.07 (35.89, 38.28)	1275.74 (1268.51, 1283.02)
Central Europe	1.32 (1.14, 1.53)	12.06 (11.48, 12.67)	236.30 (233.72, 238.90)
Central Latin America	5.24 (5.00, 5.48)	22.19 (21.52, 22.88)	387.12 (384.33, 389.92)
Central Sub-Saharan Africa	61.86 (61.15, 62.58)	114.63 (113.69, 115.59)	477.81 (475.88, 479.74)
East Asia	7.90 (7.63, 8.18)	14.85 (14.52, 15.19)	223.92 (222.59, 225.26)
Eastern Europe	7.96 (7.33, 8.63)	0.88 (0.67, 1.14)	600.33 (594.50, 606.21)
Eastern Sub-Saharan Africa	21.09 (20.71, 21.49)	203.01 (201.75, 204.26)	1347.85 (1344.63, 1351.07)
High-income Asia Pacific	1.22 (1.06, 1.40)	0.27 (0.19, 0.37)	40.85 (39.85, 41.87)
High-income North America	2.43 (2.20, 2.67)	0.52 (0.41, 0.66)	83.34 (81.87, 84.83)
North Africa and Middle East	34.89 (34.33, 35.46)	39.89 (39.38, 40.41)	909.81 (907.28, 912.34)
Oceania	0.73 (0.38, 1.30)	83.22 (78.81, 87.83)	768.12 (754.96, 781.48)
South Asia	59.36 (58.89, 59.83)	94.58 (94.07, 95.09)	2041.71 (2039.25, 2044.17)
Southeast Asia	1.94 (1.86, 2.02)	46.33 (45.75, 46.91)	688.14 (685.94, 690.34)
Southern Latin America	3.22 (2.57, 4.00)	10.99 (9.96, 12.11)	153.92 (149.67, 158.26)
Southern Sub-Saharan Africa	3.63 (3.40, 3.88)	47.50 (46.58, 48.44)	955.13 (950.99, 959.29)
Tropical Latin America	0.60 (0.52, 0.71)	48.29 (47.28, 49.33)	859.63 (855.36, 863.92)
Western Europe	2.38 (2.25, 2.51)	0.58 (0.51, 0.65)	97.75 (96.88, 98.63)
Western Sub-Saharan Africa	6.71 (6.57, 6.85)	183.15 (182.22, 184.08)	1688.03 (1685.30, 1690.77)
Incidence			
Global	259.76 (259.30, 260.21)	20426.14 (20422.19, 20430.10)	#N/A
High-middle SDI	41.05 (40.79, 41.31)	2688.27 (2686.02, 2690.51)	#N/A
High SDI	72.49 (71.63, 73.36)	1655.48 (1651.59, 1659.38)	#N/A
Low-middle SDI	333.95 (333.04, 334.86)	20438.70 (20431.50, 20445.90)	#N/A
Low SDI	447.75 (446.62, 448.89)	39506.13 (39495.73, 39516.53)	#N/A
Middle SDI	146.79 (146.12, 147.47)	11433.39 (11427.26, 11439.52)	#N/A
Central Europe, Eastern Europe, and Central Asia	44.37 (43.66, 45.09)	6507.91 (6497.84, 6517.99)	#N/A
High-income	69.76 (68.93, 70.59)	3065.20 (3059.65, 3070.75)	#N/A
Latin America and Caribbean	35.64 (35.29, 36.00)	8028.51 (8023.37, 8033.65)	#N/A
Southeast Asia, East Asia, and Oceania	31.81 (31.64, 31.97)	5114.81 (5112.56, 5117.06)	#N/A
Sub-Saharan Africa	183.33 (182.94, 183.73)	20690.63 (20686.29, 20694.96)	#N/A
North Africa and Middle East	242.93 (241.51, 244.35)	12097.35 (12087.97, 12106.74)	#N/A
South Asia	662.78 (661.23, 664.33)	16138.05 (16131.14, 16144.98)	#N/A
Andean Latin America	13.08 (12.03, 14.19)	11062.89 (11032.54, 11093.30)	#N/A
Australasia	45.85 (42.28, 49.66)	180.72 (173.58, 188.10)	#N/A
Caribbean	34.77 (33.68, 35.89)	7937.05 (7919.74, 7954.40)	#N/A
Central Asia	93.05 (90.96, 95.19)	9216.95 (9197.14, 9236.79)	#N/A
Central Europe	23.45 (22.62, 24.30)	7570.22 (7555.58, 7584.90)	#N/A
Central Latin America	83.15 (82.06, 84.25)	12338.68 (12323.13, 12354.24)	#N/A
Central Sub-Saharan Africa	897.72 (894.95, 900.50)	28403.76 (28388.80, 28418.73)	#N/A
East Asia	211.82 (210.38, 213.26)	8071.31 (8063.08, 8079.56)	#N/A
Eastern Europe	86.80 (84.57, 89.08)	705.21 (698.93, 711.53)	#N/A
Eastern Sub-Saharan Africa	287.71 (286.28, 289.14)	40751.64 (40734.46, 40768.82)	#N/A
High-income Asia Pacific	23.76 (23.00, 24.55)	728.87 (724.64, 733.12)	#N/A
High-income North America	49.91 (48.79, 51.04)	1558.11 (1551.80, 1564.45)	#N/A
North Africa and Middle East	242.93 (241.51, 244.35)	12097.35 (12087.97, 12106.74)	#N/A
Oceania	7.91 (6.58, 9.46)	21337.44 (21267.18, 21407.89)	#N/A
South Asia	662.78 (661.23, 664.33)	16138.05 (16131.14, 16144.98)	#N/A
Southeast Asia	30.76 (30.41, 31.12)	16635.89 (16624.76, 16647.02)	#N/A
Southern Latin America	42.90 (40.50, 45.41)	17081.07 (17034.39, 17127.87)	#N/A
Southern Sub-Saharan Africa	63.46 (62.44, 64.49)	12190.90 (12176.01, 12205.81)	#N/A
Tropical Latin America	12.09 (11.65, 12.55)	17480.99 (17461.69, 17500.31)	#N/A
Western Europe	50.49 (49.88, 51.10)	856.36 (853.80, 858.93)	#N/A
Western Sub-Saharan Africa	94.13 (93.56, 94.69)	32718.28 (32705.87, 32730.70)	#N/A

**Table 2:** Changing trends of age-standardized incidence and DALYs rates of nutritional deficiency in 0–14 years at the global and regional levels from 1990 to 2021.

location	Iodine deficiency	Vitamin A deficiency	Dietary iron deficiency
DALYs (disability-adjusted life years)			
Global	−1.48 (−3.11, 0.18)	−1.66 (−2.68, −0.62)	0.53 (−0.46, 1.52)
High-middle SDI	−5.39 (−7.01, −3.75)	−6.71 (−7.41, −6.00)	−4.81 (−5.49, −4.12)
High SDI	1.11 (−0.46, 2.71)	−5.22 (−5.99, −4.45)	−1.70 (−2.28, −1.11)
Low-middle SDI	−4.47 (−5.75, −3.17)	−5.10 (−6.10, −4.08)	−1.77 (−2.80, −0.72)
Low SDI	−2.13 (−3.52, −0.71)	−2.11 (−2.88, −1.34)	−0.02 (−0.75, 0.72)
Middle SDI	−7.83 (−8.75, −6.89)	−2.68 (−3.49, −1.87)	−0.50 (−1.32, 0.31)
Central Europe, Eastern Europe, and Central Asia	−4.62 (−6.29, −2.92)	−3.48 (−4.24, −2.72)	−2.02 (−2.79, −1.24)
High-income	−3.52 (−5.36, −1.64)	−3.54 (−4.56, −2.50)	−1.47 (−2.51, −0.43)
Latin America and Caribbean	−3.36 (−4.43, −2.27)	−4.36 (−5.29, −3.42)	−2.41 (−3.22, −1.59)
Southeast Asia, East Asia, and Oceania	−3.35 (−5.14, −1.52)	−3.38 (−4.50, −2.24)	−0.74 (−1.84, 0.38)
Sub-Saharan Africa	−0.87 (−1.98, 0.26)	−2.34 (−3.19, −1.48)	0.03 (−0.81, 0.87)
North Africa and Middle East	−1.42 (−3.16, 0.35)	−2.72 (−4.01, −1.42)	−0.78 (−1.99, 0.45)
South Asia	−3.37 (−5.13, −1.56)	−2.75 (−4.09, −1.40)	0.24 (−1.07, 1.57)
Andean Latin America	−1.63 (−3.04, −0.21)	−3.23 (−4.07, −2.38)	−1.89 (−2.60, −1.17)
Australasia	1.13 (−0.52, 2.80)	−2.25 (−3.61, −0.89)	−0.55 (−1.54, 0.45)
Caribbean	−4.65 (−5.95, −3.34)	−2.00 (−2.63, −1.37)	−1.15 (−1.72, −0.57)
Central Asia	−1.23 (−2.55, 0.11)	−1.69 (−2.97, −0.39)	−0.30 (−1.41, 0.83)
Central Europe	−4.82 (−6.00, −3.63)	−6.73 (−7.45, −6.00)	−4.11 (−4.67, −3.54)
Central Latin America	−1.09 (−2.27, 0.12)	−2.95 (−3.81, −2.09)	−0.94 (−1.55, −0.33)
Central Sub-Saharan Africa	−1.13 (−2.23, −0.03)	−1.86 (−2.87, −0.84)	−1.63 (−2.32, −0.93)
East Asia	−4.50 (−6.80, −2.15)	−5.89 (−7.16, −4.61)	−4.69 (−5.78, −3.59)
Eastern Europe	−4.32 (−6.54, −2.04)	−6.96 (−8.08, −5.83)	−4.17 (−5.43, −2.89)
Eastern Sub-Saharan Africa	0.00 (−1.30, 1.32)	−2.22 (−2.92, −1.51)	−0.33 (−1.01, 0.35)
High-income Asia Pacific	−3.25 (−5.46, −1.00)	−5.85 (−7.01, −4.67)	−2.75 (−3.90, −1.60)
High-income North America	1.23 (−0.40, 2.90)	−2.00 (−2.71, −1.28)	1.21 (0.57, 1.86)
North Africa and Middle East	−1.42 (−3.16, 0.35)	−2.72 (−4.01, −1.42)	−0.78 (−1.99, 0.45)
Oceania	−3.26 (−4.50, −2.01)	−1.33 (−2.39, −0.27)	0.52 (−0.40, 1.45)
South Asia	−3.37 (−5.13, −1.56)	−2.75 (−4.09, −1.40)	0.24 (−1.07, 1.57)
Southeast Asia	−6.63 (−8.52, −4.69)	−4.99 (−5.97, −4.01)	−1.30 (−2.22, −0.37)
Southern Latin America	−1.94 (−3.94, 0.10)	−3.37 (−4.28, −2.46)	−1.53 (−2.20, −0.85)
Southern Sub-Saharan Africa	−3.43 (−5.12, −1.71)	−2.23 (−3.47, −0.97)	−0.03 (−1.19, 1.14)
Tropical Latin America	0.33 (−0.84, 1.52)	−2.56 (−3.71, −1.40)	−0.39 (−1.54, 0.78)
Western Europe	−5.65 (−6.88, −4.40)	−5.89 (−6.82, −4.96)	−3.35 (−4.22, −2.47)
Western Sub-Saharan Africa	−3.12 (−4.14, −2.08)	−2.60 (−3.57, −1.61)	0.34 (−0.66, 1.36)
Incidence			
Global	−0.04 (−1.36, 1.29)	−2.02 (−3.04, −0.98)	#N/A
High-middle SDI	−4.70 (−6.11, −3.27)	−6.85 (−7.67, −6.02)	#N/A
High SDI	0.86 (−0.14, 1.87)	−3.96 (−4.38, −3.55)	#N/A
Low-middle SDI	−3.01 (−4.38, −1.63)	−5.15 (−6.12, −4.17)	#N/A
Low SDI	−1.26 (−2.39, −0.11)	−1.97 (−2.68, −1.25)	#N/A
Middle SDI	−4.13 (−5.09, −3.15)	−4.13 (−4.90, −3.36)	#N/A
Central Europe, Eastern Europe, and Central Asia	−3.40 (−4.66, −2.12)	−3.95 (−4.68, −3.21)	#N/A
High-income	−2.82 (−4.38, −1.23)	−3.67 (−4.68, −2.65)	#N/A
Latin America and Caribbean	−2.52 (−3.23, −1.80)	−4.50 (−5.34, −3.65)	#N/A
Southeast Asia, East Asia, and Oceania	−1.01 (−2.51, 0.51)	−3.09 (−4.21, −1.96)	#N/A
Sub-Saharan Africa	−0.47 (−1.53, 0.60)	−2.44 (−3.24, −1.64)	#N/A
North Africa and Middle East	−1.29 (−3.07, 0.51)	−2.65 (−3.93, −1.35)	#N/A
South Asia	−1.35 (−2.84, 0.16)	−3.29 (−4.64, −1.92)	#N/A
Andean Latin America	0.56 (−0.41, 1.54)	−2.48 (−3.24, −1.70)	#N/A
Australasia	1.13 (−0.41, 2.70)	−0.67 (−2.01, 0.69)	#N/A
Caribbean	−3.94 (−4.87, −3.01)	−3.38 (−3.98, −2.77)	#N/A
Central Asia	−1.11 (−2.35, 0.14)	−0.98 (−2.20, 0.26)	#N/A
Central Europe	−3.88 (−4.83, −2.92)	−5.48 (−6.13, −4.82)	#N/A
Central Latin America	−0.24 (−1.00, 0.54)	−2.87 (−3.45, −2.29)	#N/A
Central Sub-Saharan Africa	−1.08 (−2.22, 0.07)	−1.39 (−2.41, −0.37)	#N/A
East Asia	−2.42 (−4.39, −0.40)	−6.12 (−7.37, −4.86)	#N/A
Eastern Europe	−3.93 (−5.73, −2.09)	−6.41 (−7.63, −5.18)	#N/A
Eastern Sub-Saharan Africa	0.53 (−0.77, 1.85)	−1.89 (−2.39, −1.39)	#N/A
High-income Asia Pacific	−2.64 (−4.46, −0.79)	−4.89 (−6.38, −3.37)	#N/A
High-income North America	1.30 (0.24, 2.38)	−2.34 (−3.08, −1.59)	#N/A
North Africa and Middle East	−1.29 (−3.07, 0.51)	−2.65 (−3.93, −1.35)	#N/A
Oceania	−3.13 (−4.23, −2.02)	−1.50 (−2.51, −0.48)	#N/A
South Asia	−1.35 (−2.84, 0.16)	−3.29 (−4.64, −1.92)	#N/A
Southeast Asia	−4.50 (−5.80, −3.18)	−3.82 (−4.81, −2.82)	#N/A
Southern Latin America	−1.86 (−3.31, −0.38)	−2.43 (−3.53, −1.32)	#N/A
Southern Sub-Saharan Africa	−2.30 (−3.84, −0.73)	−2.82 (−3.91, −1.72)	#N/A
Tropical Latin America	0.67 (−0.69, 2.05)	−2.50 (−3.53, −1.47)	#N/A
Western Europe	−5.22 (−6.25, −4.19)	−5.78 (−6.37, −5.20)	#N/A
Western Sub-Saharan Africa	−2.73 (−3.77, −1.69)	−2.77 (−3.71, −1.84)	#N/A

### 3.2 Age distribution

According to the 2021 Global Burden of Disease data, the age-specific disease burden for nutritional deficiencies among children aged 0–14 exhibited pronounced regional heterogeneity. A temporal trend analysis revealed the most substantial average annual decline in DALY rates for iodine deficiency in the 5–9 years age group (EAPC = −1.89). Vitamin A deficiency demonstrated a more pronounced EAPC decrease (−1.92) in the 1–4 year-old group compared to other age groups. Notably, the age-specific burden for dietary iron deficiency stabilized in prepubertal children aged 10–14 (EAPC = 0.12). The GBD 2021 data indicated that the age-specific disease burden for nutritional deficiencies in children aged 0–14 exhibited a bimodal distribution: children aged 5–9 contributed the most to the DALY rate (35–45% of the total burden), followed by the 1–4 year-old group (30–38%). This suggests that the preschool to school age bracket is a critical phase for the onset of nutritional deficiencies and a pivotal window for intervention (Supplementary Tables 1, 2 and Supplementary Figures 1–5). Further examination of the age-specific incidence rates (Supplementary Table 2) revealed that the burden of both iodine and vitamin A deficiencies was lowest among infants (<1 year), rapidly increased during early childhood (2–4 years), and peaked in middle childhood (5–9 years). For instance, in several low- and middle-SDI countries such as Angola, Burundi, and India, the incidence of iodine deficiency rose more than tenfold from infancy to the 5–9 year age group. In contrast, a gradual decline was observed during adolescence (10–14 years), suggesting that improved dietary diversity and school-based nutrition interventions may mitigate risk in this group. This age-related pattern underscores that preschool and early school-aged children represent the most vulnerable stages for micronutrient deficiencies. Therefore, fortification programs and supplementation policies should particularly emphasize children aged 1–9 years, aligning with the WHO and UNICEF recommendations for targeted vitamin A and iodine interventions in this developmental window.

### 3.3 Trends in SDI distribution

According to the 2021 GBD data, there is a marked disparity in the regional distribution of disease burdens for different nutritional deficiencies, as measured by SDI. For iodine deficiency, the age-standardized DALY rates peaked in low SDI regions (34.20/100,000) and dipped to their lowest in high SDI regions (4.82/100,000). The age-standardized DALY rates for vitamin A deficiency were notably elevated in low SDI regions (218.63/100,000), plunging to minimal levels in high SDI regions (1.47/100,000). Meanwhile, the age-standardized DALY rates for dietary iron deficiency were highest in low-to-middle SDI areas (1999.55/100,000), and diminished substantially in high SDI regions (137.88/100,000). When examining age-standardized incidence rates, iodine deficiency was most prevalent in low SDI regions (447.75/100,000) and least common in high SDI regions (72.49/100,000). Conversely, the age-standardized incidence rate of vitamin A deficiency peaked in low SDI regions (39506.13/100,000) and diminished substantially in high SDI regions (1655.48/100,000). An analysis of the EAPC revealed that there was no significant improvement in the global age-standardized DALY rate for iodine deficiency in high SDI regions, with the steepest decline observed in middle SDI regions (EAPC = −7.83). For vitamin A deficiency, the decline in age-standardized DALY rates was more accentuated in high-to-middle SDI regions (EAPC = −6.71), whereas the slowest decrease was noted in low SDI regions (EAPC = −2.11). The EAPC for dietary iron deficiency did not show statistical significance in either low or middle SDI regions, with the most substantial reduction observed in the high-to-middle SDI region (EAPC = −4.81) (Tables 1, 2 and Figures 1, 2).

The correlation analysis conducted on 21 regions and 204 countries revealed that regions with a low SDI typically exhibited a high burden, low improvement pattern. For instance, South Asia, despite a decline in the DALY rate (−3.37), still held the highest absolute value (2041.71/100,000). In contrast, medium and high SDI regions, such as Tropical Latin America, experienced an increase in the DALY rate (0.34). Notably, high SDI regions achieved an annual reduction in vitamin A deficiency incidence rate of −5.22% due to intensive interventions. These regional disparities underscore the necessity for nutritional interventions to be finely tuned to the specific SDI developmental level. For example, regions prone to conflict (such as Afghanistan with an EAPC of −1.17) should prioritize fundamental nutritional support, whereas areas undergoing dietary transitions (e.g., Latin America) must remain vigilant about concealed risks (Tables 1, 2).

### 3.4 Area

According to the GBD 2021 data, notable regional variations exist in the subtypes of nutritional deficiencies among children aged 0–14 years. For iodine deficiency, the three regions with the highest age-standardized DALY rates are South Asia (59.36/100,000), Central Africa (61.86/100,000), and West Africa (63.46/100,000). Conversely, the lowest rates are observed in Tropical Latin America (0.60/100,000), Oceania (0.73/100,000), and Andean Latin America (0.73/100,000). The most significant EAPC declines were noted in Southeast Asia (−6.63), Western Europe (−5.65), and Central Europe (−4.82), whereas a minor upward trend was identified in Tropical Latin America (0.33). Age-standardized DALY rates for vitamin A deficiency peaked in East Africa (203.01/100,000), West Africa (1688.03/100,000), and Central Africa (114.63/100,000) but were lowest in Australasia (0.11/100,000), high-income Asia and the Pacific (0.27/100,000), and high-income North America (0.52/100,000). The most pronounced decreases in EAPC were recorded in Eastern Europe (−6.96), Central Europe (−6.73), and East Asia (−5.89). Dietary iron deficiency’s age-standardized DALY rates were elevated in East Africa (1347.85/100,000), West Africa (1688.03/100,000), and South Asia (2041.71/100,000). The lowest rates were documented in high-income Asia-Pacific (40.85/100,000), Australasia (78.75/100,000), and high-income North America (83.34/100,000). Notable reductions in EAPC were from East Asia (−4.69), Eastern Europe (−4.17), and Central Europe (−4.11), whereas the situation in West Africa (0.34) and tropical Latin America (0.39) remained stagnant or worsened slightly. In terms of age-standardized incidence rate, the three most significant reductions in iodine deficiency EAPCs were observed in Western Europe (−5.22), Southeast Asia (−4.50), and Eastern Europe (−3.93). Additionally, notable decreases in vitamin A deficiency EAPCs were found in Eastern Europe (−6.41), East Asia (−6.12) and Western Europe (−5.78) (Tables 1, 2; Supplementary Table 3; Figures 1, 2).

### 3.5 National trends

According to the 2021 GBD data, significant country-level disparities exist in the subtypes of nutritional deficiencies among children aged 0–14 years. In the case of iodine deficiency, the three countries with the highest age-standardized DALY rates are Burkina Faso (84.32/100,000), Niger (79.87/100,000), and Mali (75.14/100,000). Conversely, the countries with the lowest rates are Paraguay (0.23/100,000), Uruguay (0.31/100,000), and Chile (0.38/100,000). The countries that have experienced the most significant reductions in the EAPC include the Maldives (−7.86), Cambodia (−7.24), and Rwanda (−6.98). Meanwhile, an upward trend is observed in Ecuador (0.71) and Venezuela (0.45). Vitamin A deficiency DALY rates peaked in Somalia (690.83/100,000), South Sudan (645.19/100,000), and the Central African Republic (587.34/100,000), while the lowest rates were recorded in Singapore (0.07/100,000), Qatar (0.15/100,000), and the UAE (0.21/100,000). The most significant declines in EAPC were observed in Thailand (−9.01), Maldives (−8.34), and Taiwan, China (−7.50), although Benin (0.57) and Zambia (0.96) exhibited increasing DALY rates. The highest age-standardized DALY rates for dietary iron deficiency were found in Burkina Faso (2325.45/100,000), Mali (2218.73/100,000), and Niger (2109.84/100,000), whereas the lowest rates were in Japan (37.56/100,000), Singapore (43.18/100,000), and South Korea (48.92/100,000). Notable reductions in EAPC were seen in Mongolia (−6.81), Bulgaria (−5.63), and Ukraine (−5.27), but the burden persisted in Libya (1.02) and Iraq (0.73). In terms of age-standardized incidence rate, the greatest reductions in iodine deficiency EAPC were in Sri Lanka (−8.14), Maldives (−7.86), and Cambodia (−7.24). Significant reductions in vitamin A deficiency EAPC were noted in Thailand (−9.01), Maldives (−8.34), and Taiwan, China (−7.50) (Supplementary Tables 4–6 and Supplementary Figures 6, 7).

### 3.6 Trends in projected burdens

**Figure 3:**
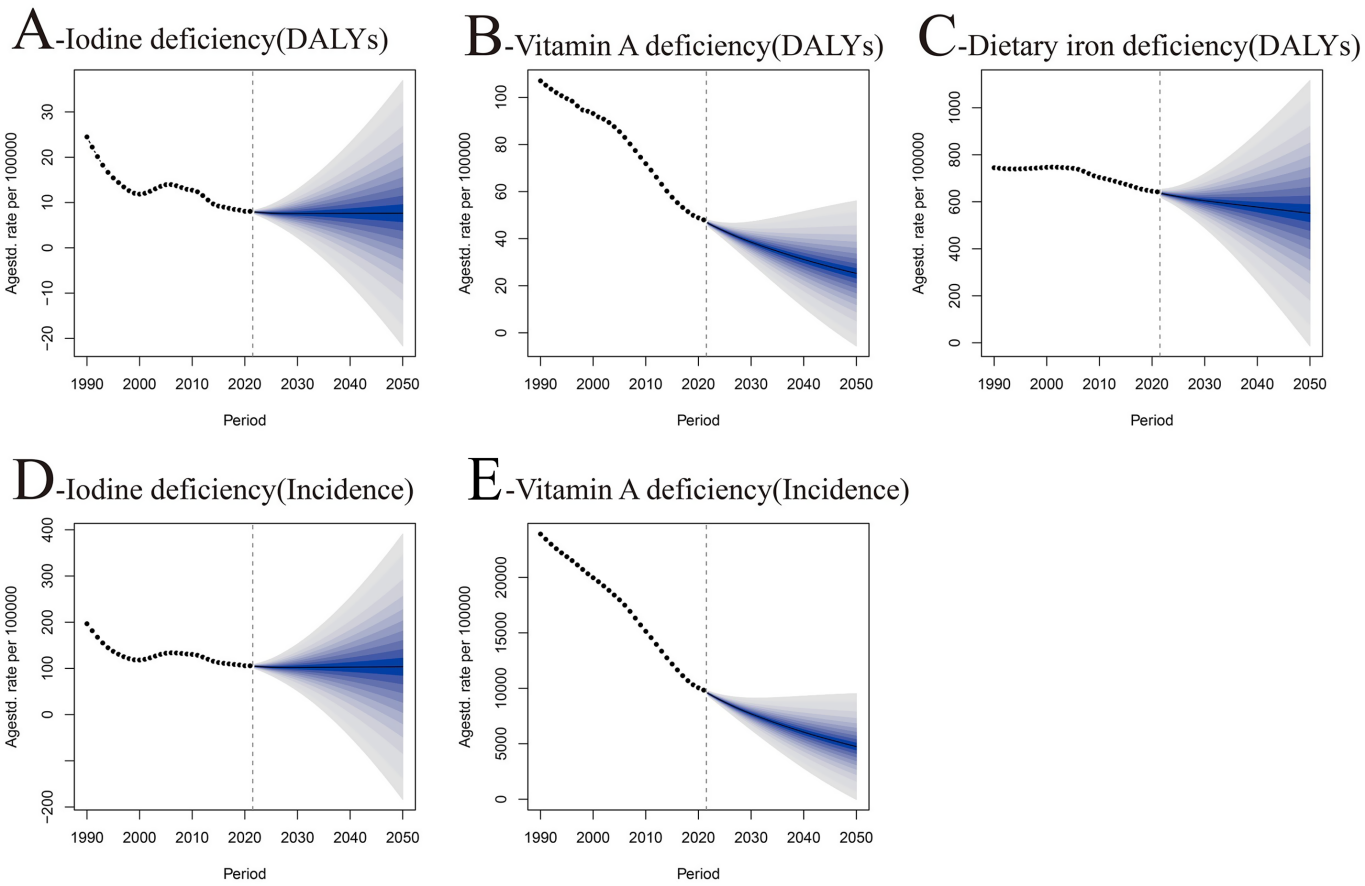
Projected trends of age-standardized incidence and DALY rates of nutritional deficiencies among children aged 0–14 years at the global and regional levels from 2022 to 2050. **(A)** Age-standardized DALY rate of iodine deficiency (per 100,000 population). **(B)** Age-standardized DALY rate of vitamin A deficiency (per 100,000 population). **(C)** Age-standardized DALY rate of dietary iron deficiency (per 100,000 population). **(D)** Age-standardized incidence rate of iodine deficiency (per 100,000 population). **(E)** Age-standardized incidence rate of vitamin A deficiency (per 100,000 population). *X*-axis = year (2022–2050); *Y*-axis = rate (per 100,000 population). All projected trends were estimated using Bayesian age-period-cohort models with 95% uncertainty intervals. SDI, sociodemographic index; DALY, disability-adjusted life year.

Based on data projections spanning from 2022 to 2050, Figures 3A–E depict the trends in DALYs and incidence rates for iodine deficiency, vitamin A deficiency, and dietary iron deficiency. Both the DALYs (Figure 3A) and incidence rates (Figure 3D) for iodine deficiency exhibit a decreasing trajectory and are anticipated to reduce markedly by 2050. Conversely, the DALYs for vitamin A deficiency (Figure 3B) and its incidence (Figure 3E) demonstrate a significant decline and are projected to approach zero by 2050. The DALY for dietary iron deficiency (Figure 3C) commences a notable decline post-2020 and is expected to diminish substantially by 2050. However, the extensive projection interval indicates a degree of uncertainty.

## 4 Discussion

This study, utilizing data from GBD 2021, revealed a substantial decline in the burden of disease associated with iodine deficiency and vitamin A deficiency among children aged 0–14 between 1990 and 2021. Specifically, age-standardized incidence rates diminished by 92.9 and 94.9%, while DALY rates reduced by 98.7 and 92.8%, respectively. These significant reductions can largely be attributed to the widespread implementation of salt iodization policies (17, 18) and the promotion of vitamin A supplementation programs (19). These findings align with UNICEF’s State of the World’s Children 2023 and WHO’s Global Nutrition Monitoring Report 2024, both emphasizing the need to strengthen micronutrient fortification and supplementation systems in low- and middle-SDI regions. Conversely, there was no notable improvement in the DALY rate for dietary iron deficiency. This stagnation may be linked to the issue of hidden hunger, which is prevalent due to the refined dietary structure in middle and high SDI regions and the inadequate supply of essential nutrients in low SDI regions (10). Analysis of regional heterogeneity indicated that South Asia and West Africa remain areas with a high burden (for instance, the highest DALY rate for vitamin A deficiency was recorded at 690.83/100,000), whereas high SDI areas have achieved an average annual reduction exceeding 5% through rigorous intervention measures. Moreover, an increasing trend of EAPC in conflict-affected countries (such as Libya) and regions undergoing dietary transition (such as tropical Latin America) suggests that political stability and shifts in dietary patterns have emerged as critical factors impacting the effectiveness of nutrition interventions (20).

Based on data from GBD 2021, the disease burden linked to nutritional deficiencies in children aged 0–14 exhibits a pronounced age-specific distribution. This trend is shaped by various factors, including regional development levels, public health interventions, and physiological requirements (18). With respect to iodine deficiency, the most notable decline in DALY rates was noted in the 5–9-year-old cohort. This decline can largely be attributed to the global promotion of iodized salt fortification. However, progress was compromised due to disruptions in the iodized salt supply chain in West Africa, exacerbated by war conditions and a predominant reliance on unfortified cassava as a staple food (21). Recent regional evaluations in South and Southeast Asia have demonstrated that strengthening monitoring systems for universal salt iodization programs and enhancing local production capacity can significantly improve program sustainability and population coverage (22). Likewise, community-based iron supplementation and food fortification initiatives in countries such as India and those in Sub-Saharan Africa have reported measurable improvements in hemoglobin levels and reductions in anemia prevalence among children and adolescents (23, 24). The incidence rate of Vitamin A deficiency decreased most markedly among children aged 1–4. Yet, the reduction in disease burden plateaued in East Africa, attributed to suboptimal measles vaccination rates (only 68%), inadequate integration with the vitamin A supplementation program, and rapid vitamin A depletion spurred by prevalent infectious diseases within this age group (25). It’s also worth noting that dietary iron deficiency appeared to stabilize in prepubertal children (aged 10–14), a trend closely tied to the sudden surge in iron demand during puberty and an increase in plant-based diets (which offer lower bioavailability of non-heme iron) in mid- to high SDI regions (26). Conversely, the pronounced disease burden in South Asia (with a DALY rate of 2041.71 per 100,000) is linked to maternal iron deficiency, delayed introduction of complementary foods, and a high incidence of hookworm infections, forming a detrimental cycle (27, 28).

The disease burden associated with nutritional deficiencies exhibited pronounced gradient variations across different SDI regions. In particular, the DALY rates for iodine deficiency (34.20/100,000) and vitamin A deficiency (218.63/100,000) were 7.3 and 151 times higher, respectively, in low SDI regions compared to high SDI regions. These disparities can largely be ascribed to the paucity of health resources, such as the limited coverage of iodized salt (less than 60%), inadequate nutrient density in complementary foods for infants and young children, and suboptimal vaccination coverage (20, 29). Interestingly, the DALY rate for dietary iron deficiency was unusually elevated in medium and high SDI regions, as evidenced by a rate of 17480.99/100,000 in tropical Latin America. This phenomenon might be linked to the reduced absorption of non-heme iron due to a higher reliance on plant-based diets (26, 30). EAPC analysis revealed that the annual decline in the DALY rate for iodine deficiency in Southeast Asia was −6.63, a figure attributable to the comprehensive enforcement of mandatory salt iodization policies (31). Conversely, in tropical Latin America, the DALY rate increased by 0.33, potentially due to a surge in the consumption of ultra-processed foods, which may have mitigated the iron intake from traditional diets (32).

Geographically, age-standardized DALY rates were notably elevated in South Asia and West Africa. For instance, the vitamin A deficiency DALY rate in Somalia reached 690.83/100,000. In contrast, high-income Asia-Pacific and North America generally exhibited rates below 1.0/100,000. This disparity is largely attributed to inadequate complementary infant and young child food supplementation, prevalent infectious diseases, and limited health resource accessibility in regions with low SDI (18, 25). Among the 21 regions studied, South Asia, West Africa, and Central Africa consistently ranked within the top three in terms of DALY rates. These regions shared challenges such as disrupted nutrition interventions due to conflicts, vulnerable agricultural systems (e.g., Burkina Faso’s DALY rate of 2325.45/100,000), and low levels of female education (with average female education in South Asia being only 4.2 years) (25, 33). East Asia, which recorded the most significant decline in EAPC (iodine deficiency EAPC = −6.81), benefited from enhancements in nutritional surveillance systems and expanded school feeding program coverage during the EIT (31).

At the national level, Somalia holds the highest global rate of vitamin A deficiency DALY (690.83/100,000). The ongoing conflict in the country has led to an immunization coverage of less than 20%, further exacerbated by a drought-induced collapse in dietary diversity (Development Initiatives, 2023) (34, 35). In contrast, Thailand has seen a decrease in its vitamin A deficiency EAPC by 9.01, a figure closely tied to the country’s implementation of vitamin A supplementation in maternal and child health kits (with 92% coverage), and mandatory palm oil fortification policies (36). The 6.81% annual decrease in the DALY rate for dietary iron deficiency in Mongolia can be primarily attributed to the increased community penetration of the national iron fortification flour program, which covers 78% of the rural population (37, 38). However, Libya’s DALY rate, which saw an increase of 1.02%, is contrary to the trend and may be linked to post-war disruptions in the food supply chain, leading to a shortage of food of animal origin (37, 39).

The projections for 2022–2050 indicate that the public health consequences of these deficiencies are gradually diminishing. Notably, vitamin A deficiency and dietary iron deficiency exhibit the most substantial improvements. These advancements may be attributed to the global nutrition improvement programs and various public health interventions. Nevertheless, due to uncertainties in the prediction intervals for future dietary iron deficiency trends, additional observations and research are warranted. Importantly, our findings provide evidence to inform WHO and UNICEF micronutrient intervention priorities beyond 2025. By identifying regions and age groups with the highest burden of iodine, vitamin A, and dietary iron deficiencies, policymakers can prioritize interventions to maximize health impact. For example, low-SDI regions with persistently high DALY rates should continue to receive targeted nutrient supplementation, whereas medium- and high-SDI regions should focus on addressing hidden hunger through dietary diversification and fortification programs. These targeted strategies are in alignment with the Global Nutrition Targets 2030, particularly goals to reduce stunting, anemia, and micronutrient deficiencies among children under 14 years. Additionally, the age-specific burden data underscore the importance of prioritizing interventions for children aged 1–9 years, a critical window for effective supplementation and fortification programs.

This study utilizes data from the GBD to conduct a comprehensive analysis of the disease burden associated with nutritional deficiencies—specifically iodine, vitamin A, and dietary iron deficiencies—among children aged 0–14 years in 2021. The analysis spans a 31-year period from 1990–2021 and is characterized by several strengths. These include global representativeness, as the study incorporates data from multiple countries and regions with a particular focus on areas with low SDI to highlight disparities in developmental levels. Additionally, the study employs a comprehensive set of assessment indicators such as morbidity and DALY rates, which amalgamate mortality and health loss data. The study’s approach also involves a segmented analysis by age, sex, and SDI to identify populations at heightened risk, for instance, areas with low SDI suffering from acute vitamin A deficiency. However, the study is not without significant limitations. The first of these is a potential bias in data quality, which could have influenced the results. For instance, the study’s reliance on model estimation for incidence rate and DALY data in low-income regions such as Sub-Saharan Africa may have skewed the results. Secondly, the study’s control for confounding variables is insufficient. Trend analyses did not adequately account for disturbances from supplementation programs (such as vitamin A fortification policies) or geopolitical events (for example, the conflict between Russia and Ukraine). Thirdly, the study’s indicators and narrow disease coverage could limit its findings. The DALY calculation depends on disability weighting assumptions, which might underestimate the impact of mild nutritional deficiencies. Furthermore, the study does not cover key nutrients such as zinc and vitamin D, thereby potentially overlooking the cumulative effects of compound malnutrition. Finally, the study’s data only goes up to 2021, and therefore does not reflect the impact of recent global events such as the COVID-19 pandemic or climate change on nutrition services. In conclusion, while this study provides an important foundation for developing intervention strategies, it should be supplemented with dynamic monitoring and local data to optimize policy formulation. Additionally, as this study relies on secondary modeling data from the GBD database, it may not fully capture underreporting or data gaps in specific regions, particularly in low-income countries where surveillance systems are less developed. Moreover, the GBD framework partially relies on proxy data (such as food balance sheets and household dietary surveys) to estimate micronutrient intake levels, especially for iron and vitamin A. This indirect estimation approach may not accurately reflect true population-level deficiencies, particularly subclinical forms that are not captured by clinical or biomarker surveillance. Consequently, the GBD methodology may underestimate the actual burden of hidden or mild nutritional deficiencies. Furthermore, the GBD classification framework defines nutritional deficiencies mainly based on dietary intake, excluding deficiencies resulting from malabsorption, chronic illness, or other etiologies. This restriction may lead to an underestimation of the overall disease burden and a partial understanding of real-world nutritional challenges. While the temporal trends are comprehensively presented, this study does not conduct a mechanistic or causal analysis of the pathways underlying these trends, nor does it quantitatively evaluate the direct effects of interventions such as salt iodization, food fortification, or vitamin supplementation programs. Future research integrating country-specific policy timelines and intervention data is warranted to better elucidate these causal mechanisms. Although the Global Burden of Disease database is publicly accessible, replicating the complex modeling and projection processes employed in this study requires advanced statistical and programming expertise, which may limit full reproducibility for general researchers.

## 5 Conclusion

This study, utilizing data from the GBD, systematically uncovers significant regional disparities in childhood nutritional deficiencies (iodine, vitamin A, and dietary iron deficiencies) in 2021. Particularly burdened are low SDI regions, with the rate of DALY for vitamin A deficiency being 147 times higher compared to high-SDI regions. Similarly, countries like Somalia and Syria exhibit some of the highest global rates of DALY for iodine deficiency. Long-term trends indicate a yearly decline in the global DALY rate for vitamin A deficiency; however, this decline is notably limited in low SDI regions. Furthermore, improvements in dietary iron deficiency appear to have stagnated, suggesting a need for targeted and intensified interventions. Large disparities exist between countries such as Mali, where the DALY rate for vitamin A deficiency is 3,261 times higher than in Switzerland, and Zambia, where the DALY rate for iron deficiency is 128 times higher than in Singapore. These disparities underscore gaps in resource allocation and policy effectiveness.

The findings of this study furnish a critical foundation for precise interventions, such as prioritizing vitamin A supplementation and salt iodization in areas with low SDI, and coupling agriculture-nutrition policies to mitigate iron deficiency. Future research should expand to encompass zinc, vitamin D, and other nutrients, reinforce data monitoring in low-income countries to diminish modeling bias, and advocate for multisectoral collaborative interventions to tackle complex malnutrition issues. Although the current study has limitations concerning data quality and coverage, the quantitative evidence it presents underscores the imperative to prioritize global child nutrition policies. This emphasis highlights the necessity of transitioning from a disease treatment approach to a risk prevention strategy, leveraging dynamic data-driven and cross-sectoral collaboration to optimize nutritional health equity.

## Data Availability

Publicly available datasets were analyzed in this study. This data can be found here: Global Burden of Disease Study 2021 (https://www.healthdata.org/).

## References

[1] Pearce EN. Iodine deficiency in children. Endocr Dev. (2014) 26:130–8. doi: 10.1159/000363160, 25231449

[2] Imdad A Yakoob MY Sudfeld C Haider BA Black RE Bhutta ZA. Impact of vitamin A supplementation on infant and childhood mortality. BMC Public Health. (2011) 11:S20. doi: 10.1186/1471-2458-11-S3-S20 21501438 PMC3231894

[3] Wiseman EM Bar-El Dadon S Reifen R. The vicious cycle of vitamin A deficiency: a review. Crit Rev Food Sci Nutr. (2017) 57:3703–14. doi: 10.1080/10408398.2016.1160362, 27128154

[4] Bathla S Arora S. Prevalence and approaches to manage iron deficiency anemia (IDA). Crit Rev Food Sci Nutr. (2022) 62:8815–28. doi: 10.1080/10408398.2021.1935442, 34096415

[5] Tam E Keats EC Rind F das JK Bhutta ZA. Micronutrient supplementation and fortification interventions on health and development outcomes among children under-five in low- and middle-income countries: a systematic review and meta-analysis. Nutrients. (2020) 12:289. doi: 10.3390/nu12020289, 31973225 PMC7071447

[6] Moore Heslin A McNulty B. Adolescent nutrition and health: characteristics, risk factors and opportunities of an overlooked life stage. Proc Nutr Soc. (2023) 82:142–56. doi: 10.1017/S0029665123002689, 36924388

[7] Norris SA Frongillo EA Black MM Dong Y Fall C Lampl M . Nutrition in adolescent growth and development. Lancet. (2022) 399:172–84. doi: 10.1016/S0140-6736(21)01590-7, 34856190

[8] Ali F Mouzaki M. Nutritional deficiencies in children. Curr Opin Gastroenterol. (2024) 40:106–11. doi: 10.1097/MOG.0000000000000998, 38190349

[9] Yue T Zhang Q Li G Qin H. Global burden of nutritional deficiencies among children under 5 years of age from 2010 to 2019. Nutrients. (2022) 14:2685. doi: 10.3390/nu14132685, 35807863 PMC9268233

[10] Liang S Xi SZ Liu JY Tang GC Zhang WG Guo XR . Global burden and cross-country inequalities of nutritional deficiencies in adults aged 65 years and older, 1990–2021: population-based study using the GBD 2021. BMC Geriatr. (2025) 25:74. doi: 10.1186/s12877-025-05728-9, 39893435 PMC11786432

[11] Bayati M Arkia E Emadi M. Socio-economic inequality in the nutritional deficiencies among the world countries: evidence from Global Burden of Disease Study 2019. J Health Popul Nutr. (2025) 44:8. doi: 10.1186/s41043-025-00739-z, 39806471 PMC11731139

[12] Requejo J Strong K Agweyu A Billah SM Boschi-Pinto C Horiuchi S . Measuring and monitoring child health and wellbeing: recommendations for tracking progress with a core set of indicators in the Sustainable Development Goals era. Lancet Child Adolesc Health. (2022) 6:345–52. doi: 10.1016/S2352-4642(22)00039-6, 35429452 PMC9764429

[13] GBD 2021 Low Back Pain Collaborators. Global, regional, and national burden of low back pain, 1990–2020, its attributable risk factors, and projections to 2050: a systematic analysis of the Global Burden of Disease Study 2021. Lancet Rheumatol. (2023) 5:e316–29. doi: 10.1016/S2665-9913(23)00098-X 37273833 PMC10234592

[14] Han L Zhao T Zhang R Hao Y Jiao M Wu Q . Burden of nutritional deficiencies in China: findings from the Global Burden of Disease Study 2019. Nutrients. (2022) 14:3919. doi: 10.3390/nu14193919, 36235572 PMC9570758

[15] Zhang Y Feng L Zhu Z He Y Li X. Global burden of myocarditis in youth and middle age (1990–2019): a systematic analysis of the disease burden and thirty-year forecast. Curr Probl Cardiol. (2024) 49:102735. doi: 10.1016/j.cpcardiol.2024.102735, 38950720

[16] Riebler A Held L. Projecting the future burden of cancer: Bayesian age-period-cohort analysis with integrated nested Laplace approximations. Biom J. (2017) 59:531–49. doi: 10.1002/bimj.201500263, 28139001

[17] Delange F Bürgi H Chen ZP Dunn JT. World status of monitoring iodine deficiency disorders control programs. Thyroid. (2002) 12:915–24. doi: 10.1089/105072502761016557, 12494927

[18] Jiang W Li X Wang R du Y Zhou W. Cross-country health inequalities of four common nutritional deficiencies among children, 1990 to 2019: data from the Global Burden of Disease Study 2019. BMC Public Health. (2024) 24:486. doi: 10.1186/s12889-024-17942-y, 38360585 PMC10870451

[19] Miglietta A Imohe A Hasman A. Methodologies to measure the coverage of vitamin A supplementation: a systematic review. J Nutr Sci. (2021) 10:e68. doi: 10.1017/jns.2021.65, 34527226 PMC8411257

[20] Sayegh H Harden C Khan H Pai M Eichbaum QG Ibingira C . Global health education in high-income countries: confronting coloniality and power asymmetry. BMJ Glob Health. (2022) 7:e008501. doi: 10.1136/bmjgh-2022-008501, 35589151 PMC9121410

[21] Miller V Webb P Cudhea F Shi P Zhang J Reedy J . Global dietary quality in 185 countries from 1990 to 2018 show wide differences by nation, age, education, and urbanicity. Nat Food. (2022) 3:694–702. doi: 10.1038/s43016-022-00594-9, 37118151 PMC10277807

[22] Kuang Kuay L Ahmad NA Beng Chin T Ying Ying C Mahjom M Awaluddin SM . A 10-year impact evaluation of the Universal Salt Iodization (USI) intervention in Sarawak, Malaysia, 2008–2018. Nutrients. (2022) 14:1585. doi: 10.3390/nu14081585, 35458147 PMC9031048

[23] Kataria S Kataria S Chougule D Bharti B Rastogi A. Evaluating the impact of an Iron supplementation program for combating anemia in school-age and adolescent females by a grassroots organization in India. Cureus. (2024) 16:e75608. doi: 10.7759/cureus.75608, 39803128 PMC11724716

[24] Cozer AWD Souza FCV Santiago LD Lima MR Pimenta SJ Fernandes BL . Effects of Iron-fortified foods on the nutritional status of children residing in regions vulnerable to parasitic diseases: a systematic review. Prev Nutr Food Sci. (2024) 29:8–17. doi: 10.3746/pnf.2024.29.1.8, 38576884 PMC10987379

[25] Stevens GA Bennett JE Hennocq Q Lu Y de-Regil LM Rogers L . Trends and mortality effects of vitamin A deficiency in children in 138 low-income and middle-income countries between 1991 and 2013: a pooled analysis of population-based surveys. Lancet Glob Health. (2015) 3:e528–36. doi: 10.1016/S2214-109X(15)00039-X, 26275329

[26] Pasricha SR Tye-Din J Muckenthaler MU Swinkels DW. Iron deficiency. Lancet. (2021) 397:233–48. doi: 10.1016/S0140-6736(20)32594-0, 33285139

[27] Pradhan J Pai M Dwivedi R Mishra B Behera S Bera T . Burden of non-communicable diseases in South Asia: a decomposition analysis. J Health Popul Nutr. (2025) 44:124. doi: 10.1186/s41043-025-00827-0, 40251654 PMC12008954

[28] Ruiz de Viñaspre-Hernández R Juárez-Vela R Garcia-Erce JA Nanwani-Nanwani K González-Fernández S Gea-Caballero V . Iron deficiency anemia during pregnancy and maternal and neonatal health outcomes: a prospective study, Spain, 2021–2022. Heliyon. (2025) 11:e41565. doi: 10.1016/j.heliyon.2024.e41565, 39866440 PMC11760830

[29] Kassebaum N Kyu HH Zoeckler L Olsen HE Thomas K Pinho C . Child and adolescent health from 1990 to 2015: findings from the global burden of diseases, injuries, and risk factors 2015 study. JAMA Pediatr. (2017) 171:573–92. doi: 10.1001/jamapediatrics.2017.0250, 28384795 PMC5540012

[30] Han X Ding S Lu J Li Y. Global, regional, and national burdens of common micronutrient deficiencies from 1990 to 2019: a secondary trend analysis based on the Global Burden of Disease 2019 study. EClinicalMedicine. (2022) 44:101299. doi: 10.1016/j.eclinm.2022.101299, 35198923 PMC8850322

[31] Wei R Wang Z Zhang X Wang X Xu Y Li Q. Burden and trends of iodine deficiency in Asia from 1990 to 2019. Public Health. (2023) 222:75–84. doi: 10.1016/j.puhe.2023.06.034, 37531713

[32] Barco Leme AC Fisberg RM Veroneze de Mello A Sales CH Ferrari G Haines J . Food sources of shortfall nutrients among Latin Americans: results from the Latin American Study of Health and Nutrition (ELANS). Int J Environ Res Public Health. (2021) 18:4967. doi: 10.3390/ijerph18094967, 34067018 PMC8125540

[33] Al Daccache M Zeid BA Hojeij L Baliki G Brück T Ghattas H. Systematic review on the impacts of agricultural interventions on food security and nutrition in complex humanitarian emergency settings. BMC Nutr. (2024) 10:60. doi: 10.1186/s40795-024-00864-8, 38641632 PMC11027246

[34] Zhao T Liu S Zhang R Zhao Z Yu H Pu L . Global burden of vitamin A deficiency in 204 countries and territories from 1990–2019. Nutrients. (2022) 14:950. doi: 10.3390/nu14050950, 35267925 PMC8912822

[35] Martin-Canavate R Custodio E Yusuf A Molla D Fasbender D Kayitakire F. Malnutrition and morbidity trends in Somalia between 2007 and 2016: results from 291 cross-sectional surveys. BMJ Open. (2020) 10:e033148. doi: 10.1136/bmjopen-2019-033148, 32071180 PMC7045078

[36] Wirth JP Petry N Tanumihardjo SA Rogers LM McLean E Greig A . Vitamin A supplementation programs and country-level evidence of vitamin A deficiency. Nutrients. (2017) 9:190. doi: 10.3390/nu9030190, 28245571 PMC5372853

[37] Bromage S Daria T Lander RL Tsolmon S Houghton LA Tserennadmid E . Diet and nutrition status of Mongolian adults. Nutrients. (2020) 12:1514. doi: 10.3390/nu12051514, 32456038 PMC7284332

[38] Bromage S Ganmaa D Rich-Edwards JW Rosner B Bater J Fawzi WW. Projected effectiveness of mandatory industrial fortification of wheat flour, milk, and edible oil with multiple micronutrients among Mongolian adults. PLoS One. (2018) 13:e0201230. doi: 10.1371/journal.pone.0201230, 30070992 PMC6071971

[39] Adel ET Marie-Françoise RC Salaheddin MM Najeeb E Monem Ahmed A Ibrahim B . Nutritional status of under-five children in Libya; a national population-based survey. Libyan J Med. (2008) 3:13–9. doi: 10.3402/ljm.v3i1.4745, 21499476 PMC3074324

