# Supplementary material for: Epidemiological trends and disparities in iodine, vitamin A, and iron deficiencies among children aged 0–14 years globally, 1990–2021
Source: Front Nutr. 2025 Dec 10;12:1622945. doi: 10.3389/fnut.2025.1622945 (PMC12728350; doi:10.3389/fnut.2025.1622945)
Supplement: Supplementary file 1 [file Table_1.DOC]

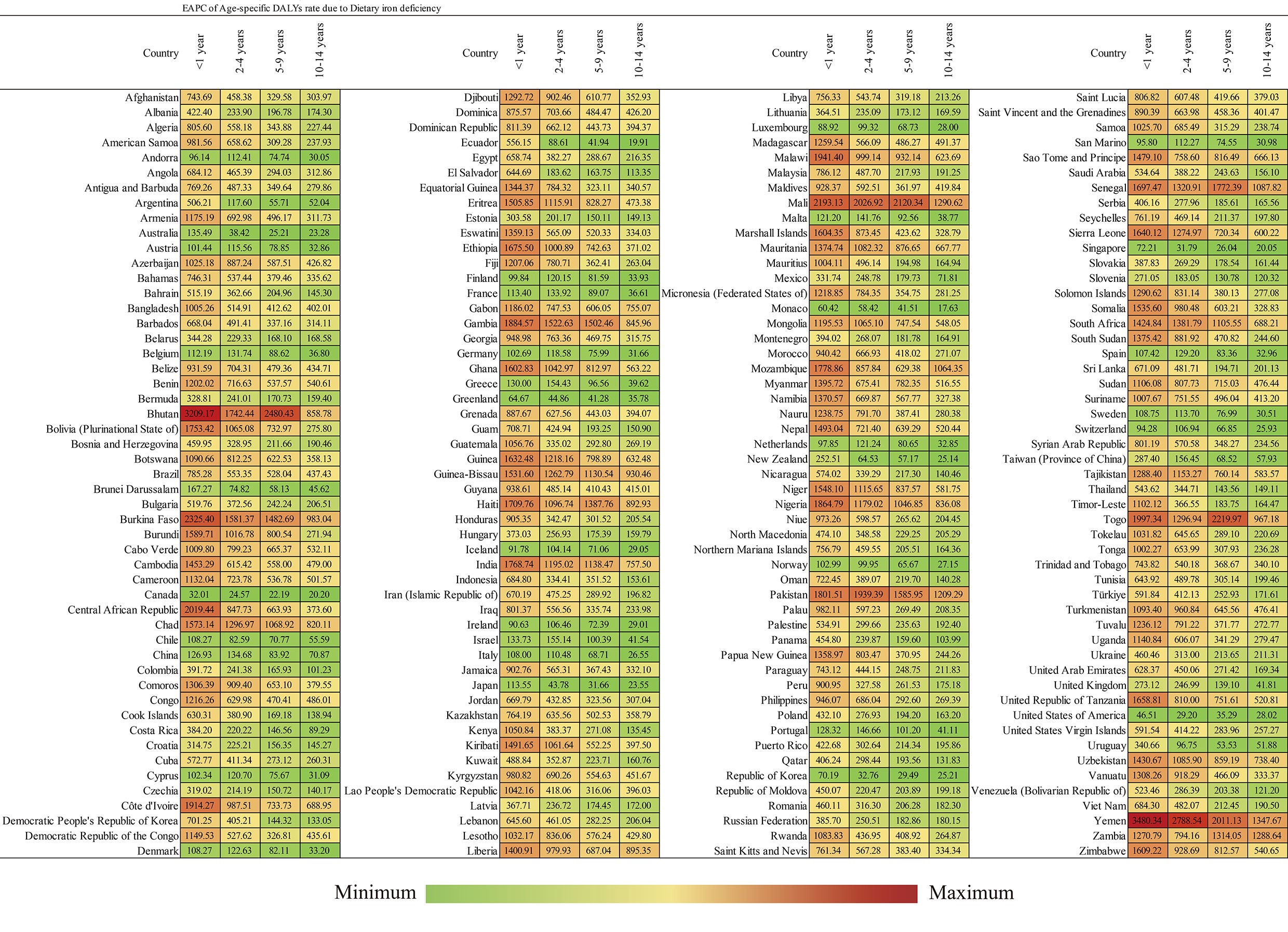


SFigure 1. Age-specific DALY rate of dietary iron deficiency in 0-14 years at the national level in 2021. DALY: disability adjusted life year


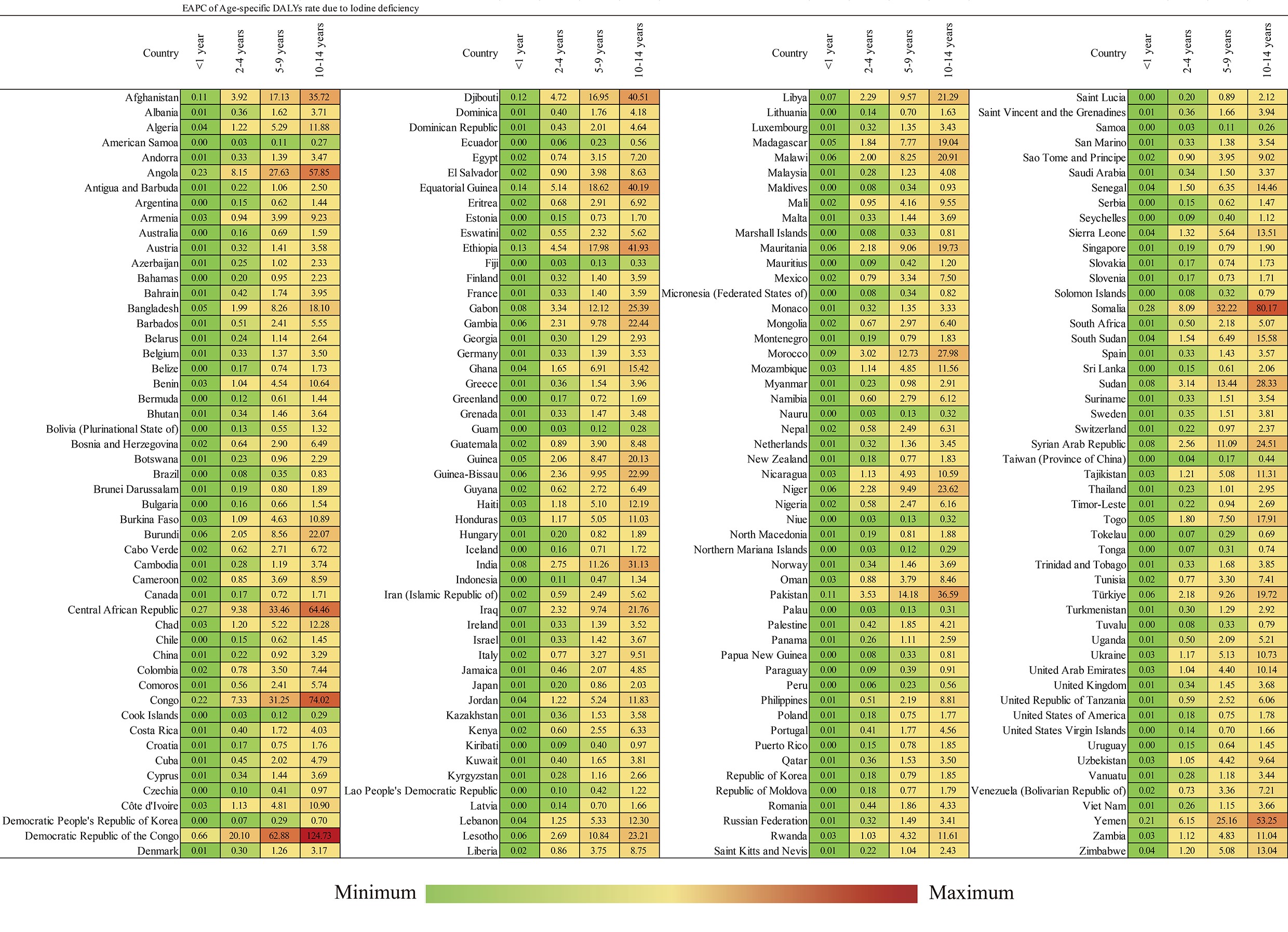


SFigure 2. Age-specific DALY rate of iodine deficiency in 0-14 years at the national level in 2021. DALY: disability adjusted life year


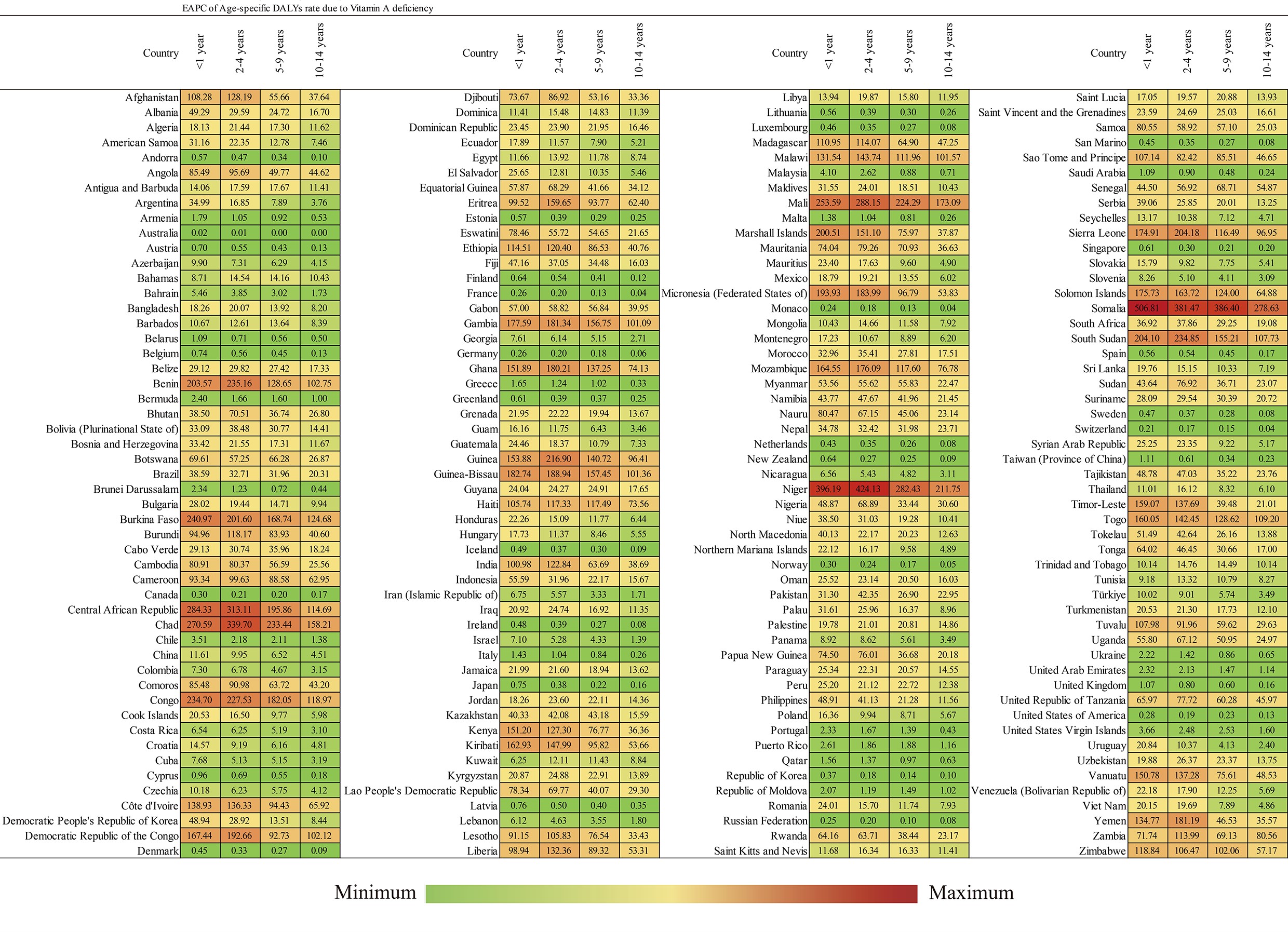


SFigure 3. Age-specific DALY rate of Vitamin A deficiency in 0-14 years at the national level in 2021. DALY: disability adjusted life year


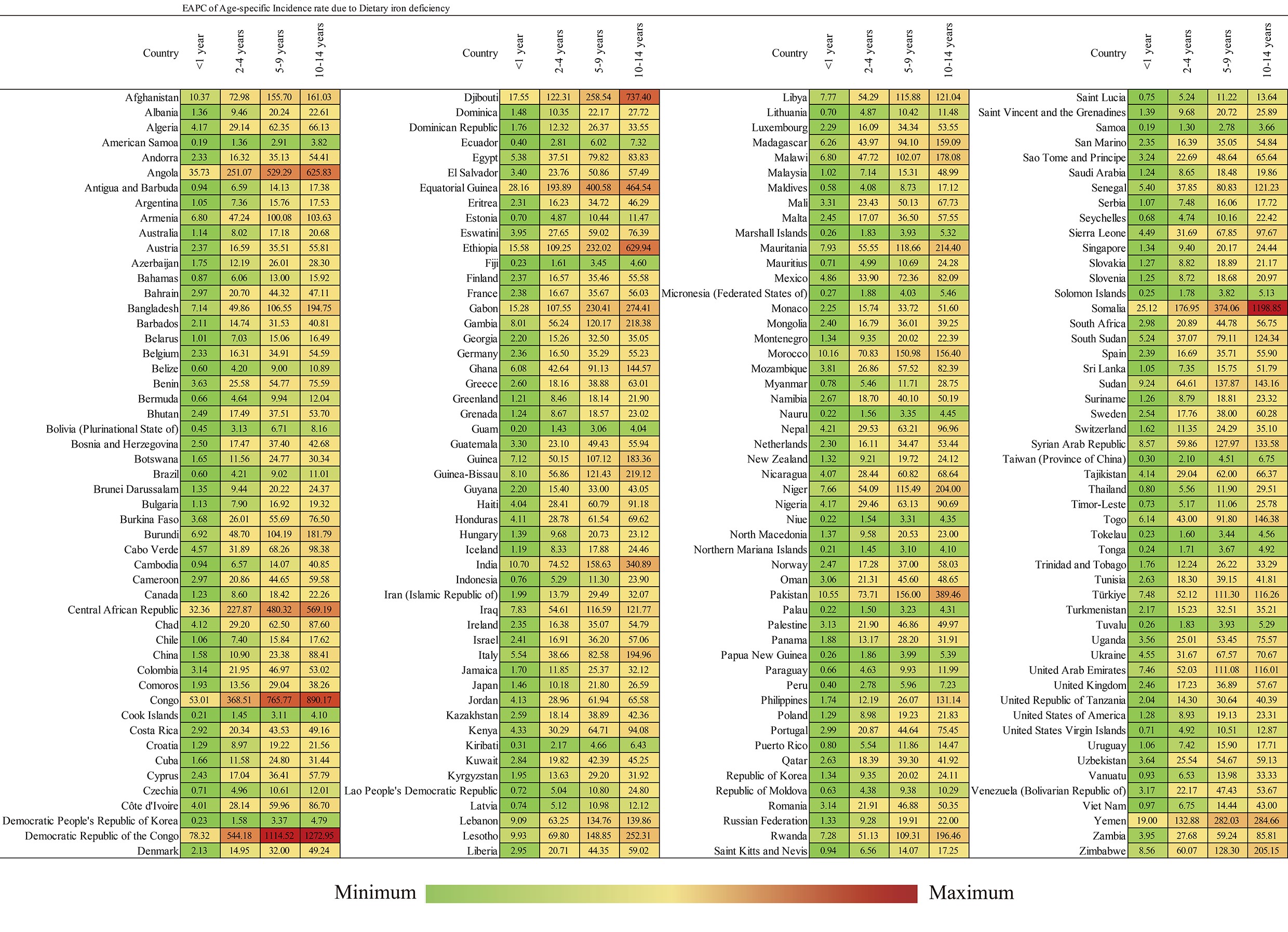


SFigure 4. Age-specific incidence rate of iodine deficiency in 0-14 years at the national level in 2021. DALY: disability adjusted life year


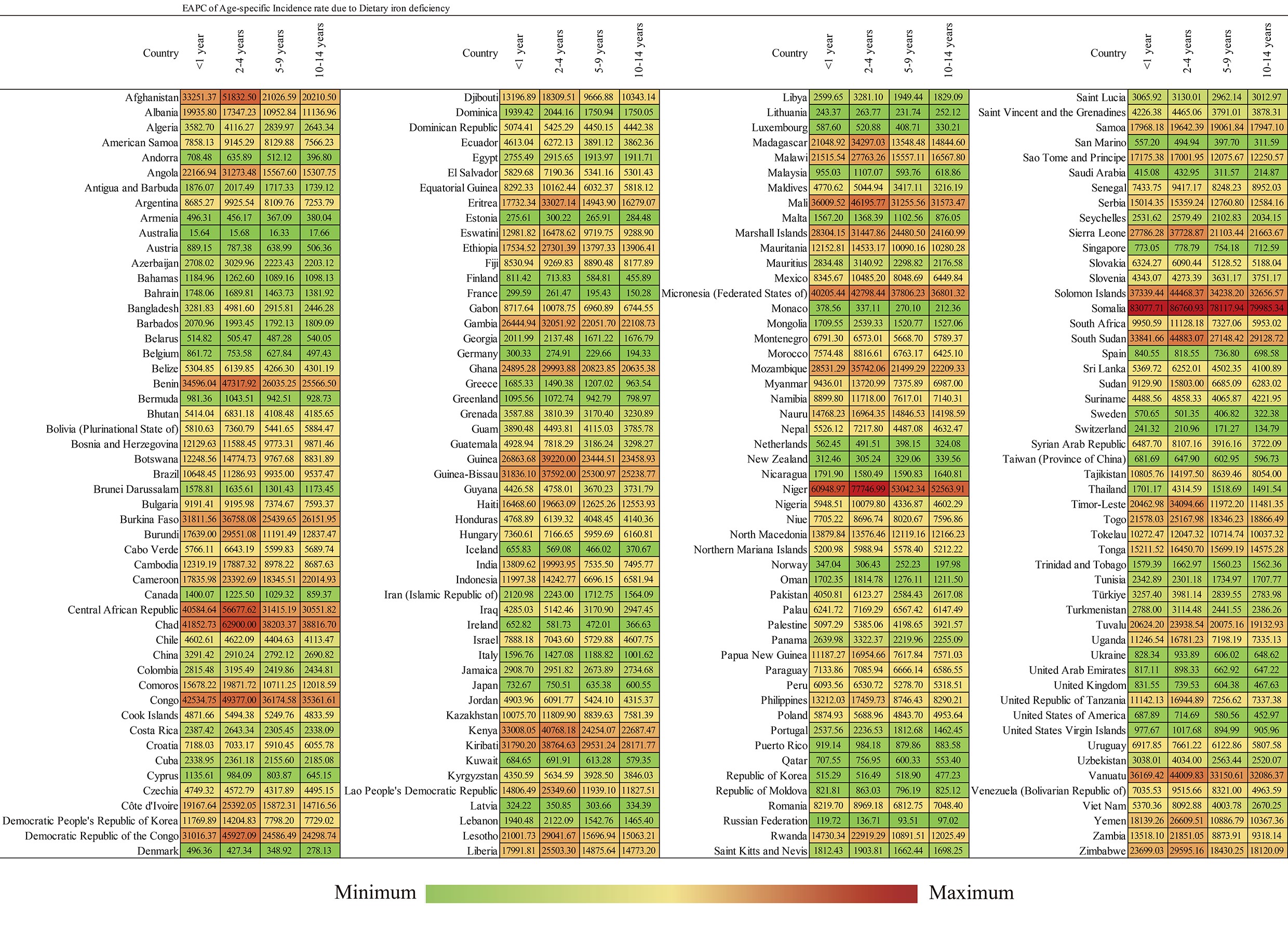


SFigure 5. Age-specific incidence rate of Vitamin A deficiency in 0-14 years at the national level in 2021. DALY: disability adjusted life year


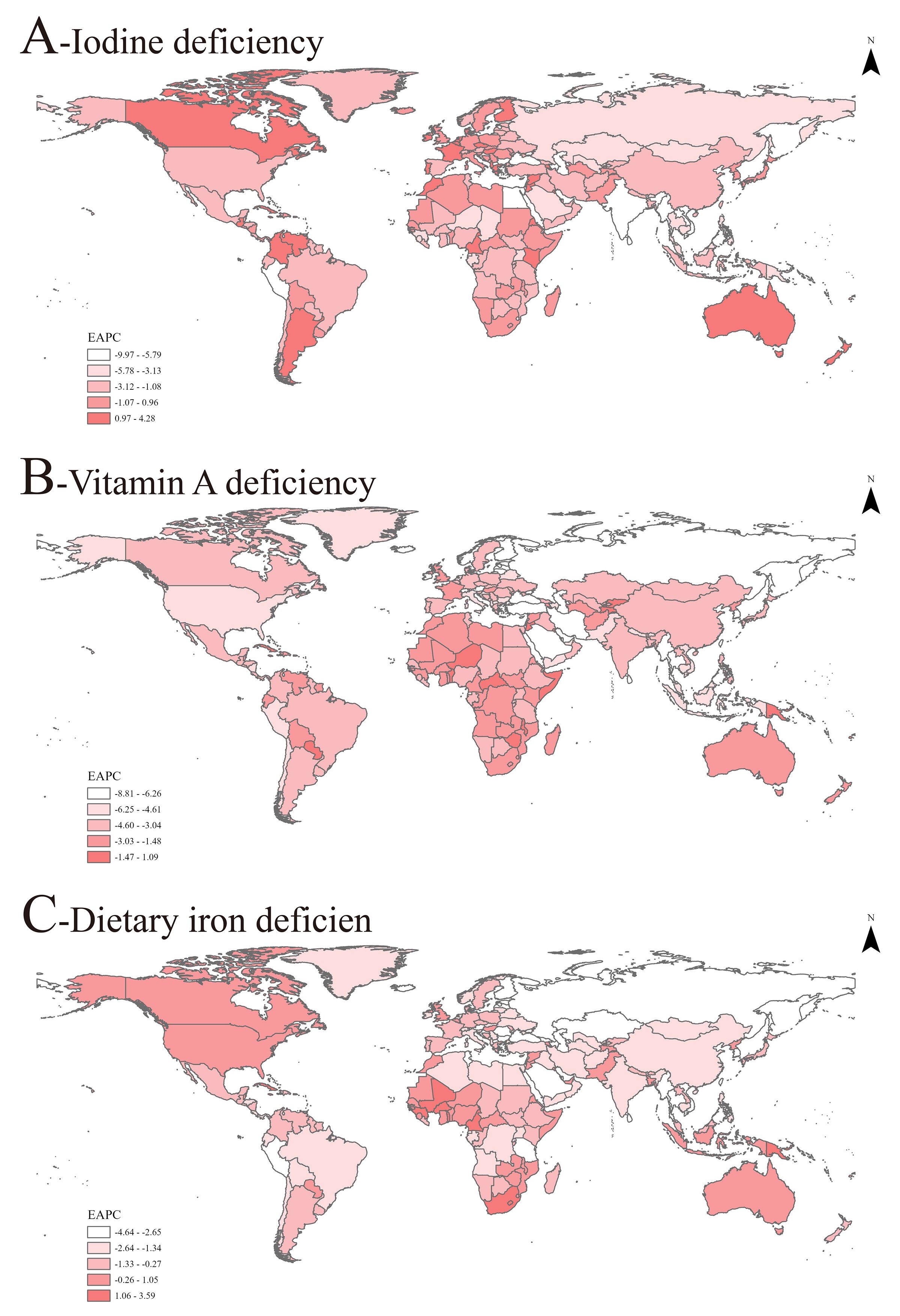


SFigure 6. Age-standardized DALYs rate of nutritional deficiency in 0-14 years at the national level and their changing trends from 1990 to 2021.

A iodine deficiency

B vitamin A deficiency

C dietary iron deficiency


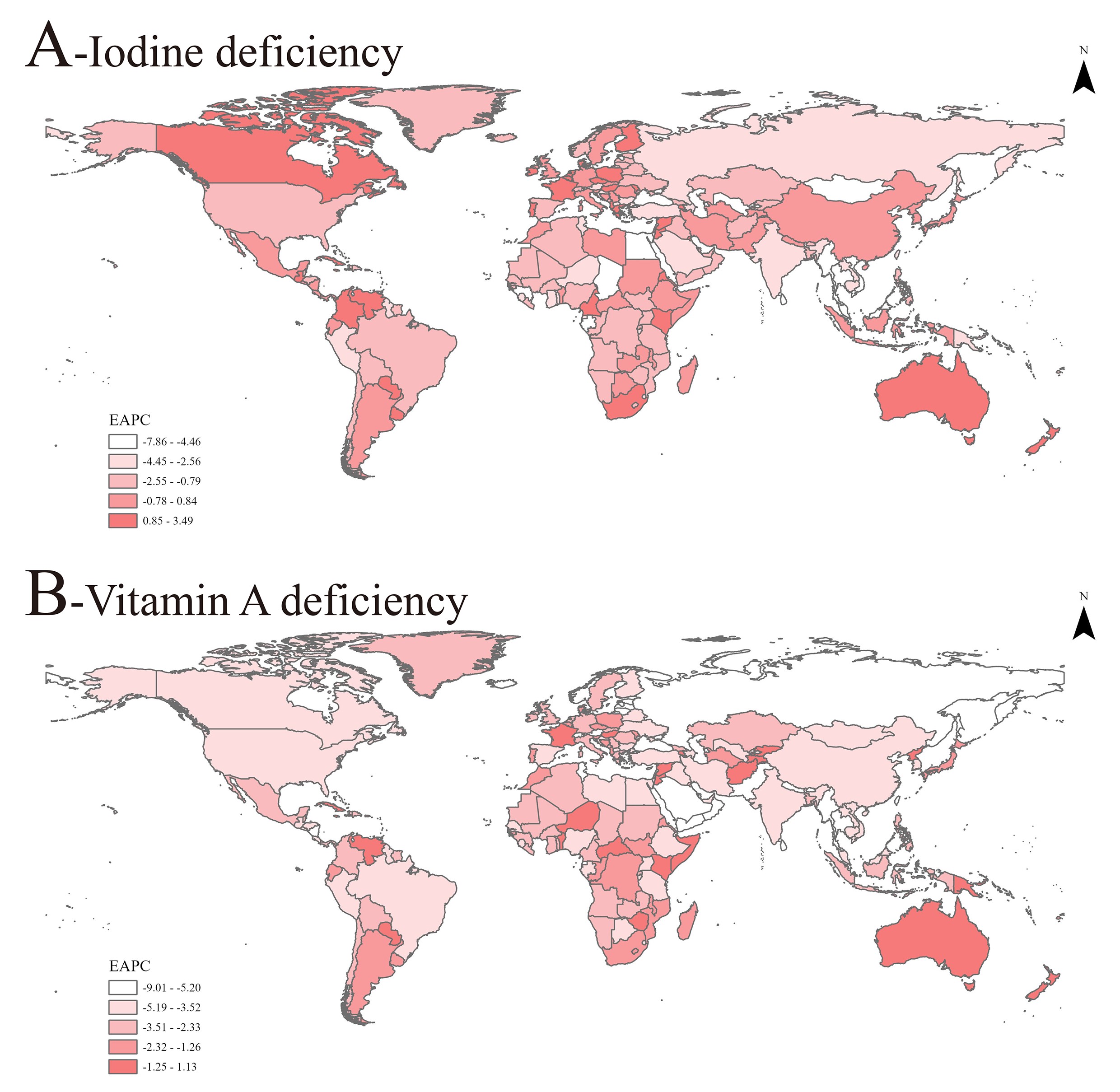


SFigure 7. Age-standardized incidence rate of nutritional deficiency in 0-14 years at the national level and their changing trends from 1990 to 2021.

A iodine deficiency

B vitamin A deficiency
