# Supplementary material for: Epidemiological trends and disparities in iodine, vitamin A, and iron deficiencies among children aged 0–14 years globally, 1990–2021
Source: Front Nutr. 2025 Dec 10;12:1622945. doi: 10.3389/fnut.2025.1622945 (PMC12728350; doi:10.3389/fnut.2025.1622945)
Supplement: Supplementary file 2 [file Table_2.DOCX]

STable 1. Age-specific DALYs rate of nutritional deficiency in 0-14 years at the national level in 2021.

|  | Iodine deficiency | Iodine deficiency | Iodine deficiency | Iodine deficiency | Vitamin A deficiency | Vitamin A deficiency | Vitamin A deficiency | Vitamin A deficiency | Dietary iron deficiency | Dietary iron deficiency | Dietary iron deficiency | Dietary iron deficiency |
| --- | --- | --- | --- | --- | --- | --- | --- | --- | --- | --- | --- | --- |
| DALYs (Disability-Adjusted Life Years) | <1 year | 2-4 years | 5-9 years | 10-14 years | <1 year | 2-4 years | 5-9 years | 10-14 years | <1 year | 2-4 years | 5-9 years | 10-14 years |
| Afghanistan | 0.11 | 3.92 | 17.13 | 35.72 | 108.28 | 128.19 | 55.66 | 37.64 | 743.69 | 458.38 | 329.58 | 303.97 |
| Albania | 0.01 | 0.36 | 1.62 | 3.71 | 49.29 | 29.59 | 24.72 | 16.70 | 422.40 | 233.90 | 196.78 | 174.30 |
| Algeria | 0.04 | 1.22 | 5.29 | 11.88 | 18.13 | 21.44 | 17.30 | 11.62 | 805.60 | 558.18 | 343.88 | 227.44 |
| American Samoa | 0.00 | 0.03 | 0.11 | 0.27 | 31.16 | 22.35 | 12.78 | 7.46 | 981.56 | 658.62 | 309.28 | 237.93 |
| Andorra | 0.01 | 0.33 | 1.39 | 3.47 | 0.57 | 0.47 | 0.34 | 0.10 | 96.14 | 112.41 | 74.74 | 30.05 |
| Angola | 0.23 | 8.15 | 27.63 | 57.85 | 85.49 | 95.69 | 49.77 | 44.62 | 684.12 | 465.39 | 294.03 | 312.86 |
| Antigua and Barbuda | 0.01 | 0.22 | 1.06 | 2.50 | 14.06 | 17.59 | 17.67 | 11.41 | 769.26 | 487.33 | 349.64 | 279.86 |
| Argentina | 0.00 | 0.15 | 0.62 | 1.44 | 34.99 | 16.85 | 7.89 | 3.76 | 506.21 | 117.60 | 55.71 | 52.04 |
| Armenia | 0.03 | 0.94 | 3.99 | 9.23 | 1.79 | 1.05 | 0.92 | 0.53 | 1175.19 | 692.98 | 496.17 | 311.73 |
| Australia | 0.00 | 0.16 | 0.69 | 1.59 | 0.02 | 0.01 | 0.00 | 0.00 | 135.49 | 38.42 | 25.21 | 23.28 |
| Austria | 0.01 | 0.32 | 1.41 | 3.58 | 0.70 | 0.55 | 0.43 | 0.13 | 101.44 | 115.56 | 78.85 | 32.86 |
| Azerbaijan | 0.01 | 0.25 | 1.02 | 2.33 | 9.90 | 7.31 | 6.29 | 4.15 | 1025.18 | 887.24 | 587.51 | 426.82 |
| Bahamas | 0.00 | 0.20 | 0.95 | 2.23 | 8.71 | 14.54 | 14.16 | 10.43 | 746.31 | 537.44 | 379.46 | 335.62 |
| Bahrain | 0.01 | 0.42 | 1.74 | 3.95 | 5.46 | 3.85 | 3.02 | 1.73 | 515.19 | 362.66 | 204.96 | 145.30 |
| Bangladesh | 0.05 | 1.99 | 8.26 | 18.10 | 18.26 | 20.07 | 13.92 | 8.20 | 1005.26 | 514.91 | 412.62 | 402.01 |
| Barbados | 0.01 | 0.51 | 2.41 | 5.55 | 10.67 | 12.61 | 13.64 | 8.39 | 668.04 | 491.41 | 337.16 | 314.11 |
| Belarus | 0.01 | 0.24 | 1.14 | 2.64 | 1.09 | 0.71 | 0.56 | 0.50 | 344.28 | 229.33 | 168.10 | 168.58 |
| Belgium | 0.01 | 0.33 | 1.37 | 3.50 | 0.74 | 0.56 | 0.45 | 0.13 | 112.19 | 131.74 | 88.62 | 36.80 |
| Belize | 0.00 | 0.17 | 0.74 | 1.73 | 29.12 | 29.82 | 27.42 | 17.33 | 931.59 | 704.31 | 479.36 | 434.71 |
| Benin | 0.03 | 1.04 | 4.54 | 10.64 | 203.57 | 235.16 | 128.65 | 102.75 | 1202.02 | 716.63 | 537.57 | 540.61 |
| Bermuda | 0.00 | 0.12 | 0.61 | 1.44 | 2.40 | 1.66 | 1.60 | 1.00 | 328.81 | 241.01 | 170.73 | 159.40 |
| Bhutan | 0.01 | 0.34 | 1.46 | 3.64 | 38.50 | 70.51 | 36.74 | 26.80 | 3209.17 | 1742.44 | 2480.43 | 858.78 |
| Bolivia (Plurinational State of) | 0.00 | 0.13 | 0.55 | 1.32 | 33.09 | 38.48 | 30.77 | 14.41 | 1753.42 | 1065.08 | 732.97 | 275.80 |
| Bosnia and Herzegovina | 0.02 | 0.64 | 2.90 | 6.49 | 33.42 | 21.55 | 17.31 | 11.67 | 459.95 | 328.95 | 211.66 | 190.46 |
| Botswana | 0.01 | 0.23 | 0.96 | 2.29 | 69.61 | 57.25 | 66.28 | 26.87 | 1090.66 | 812.25 | 622.53 | 358.13 |
| Brazil | 0.00 | 0.08 | 0.35 | 0.83 | 38.59 | 32.71 | 31.96 | 20.31 | 785.28 | 553.35 | 528.04 | 437.43 |
| Brunei Darussalam | 0.01 | 0.19 | 0.80 | 1.89 | 2.34 | 1.23 | 0.72 | 0.44 | 167.27 | 74.82 | 58.13 | 45.62 |
| Bulgaria | 0.00 | 0.16 | 0.66 | 1.54 | 28.02 | 19.44 | 14.71 | 9.94 | 519.76 | 372.56 | 242.24 | 206.51 |
| Burkina Faso | 0.03 | 1.09 | 4.63 | 10.89 | 240.97 | 201.60 | 168.74 | 124.68 | 2325.40 | 1581.37 | 1482.69 | 983.04 |
| Burundi | 0.06 | 2.05 | 8.56 | 22.07 | 94.96 | 118.17 | 83.93 | 40.60 | 1589.71 | 1016.78 | 800.54 | 271.94 |
| Cabo Verde | 0.02 | 0.62 | 2.71 | 6.72 | 29.13 | 30.74 | 35.96 | 18.24 | 1009.80 | 799.23 | 665.37 | 532.11 |
| Cambodia | 0.01 | 0.28 | 1.19 | 3.74 | 80.91 | 80.37 | 56.59 | 25.56 | 1453.29 | 615.42 | 558.00 | 479.00 |
| Cameroon | 0.02 | 0.85 | 3.69 | 8.59 | 93.34 | 99.63 | 88.58 | 62.95 | 1132.04 | 723.78 | 536.78 | 501.57 |
| Canada | 0.01 | 0.17 | 0.72 | 1.71 | 0.30 | 0.21 | 0.20 | 0.17 | 32.01 | 24.57 | 22.19 | 20.20 |
| Central African Republic | 0.27 | 9.38 | 33.46 | 64.46 | 284.33 | 313.11 | 195.86 | 114.69 | 2019.44 | 847.73 | 663.93 | 373.60 |
| Chad | 0.03 | 1.20 | 5.22 | 12.28 | 270.59 | 339.70 | 233.44 | 158.21 | 1573.14 | 1296.97 | 1068.92 | 820.11 |
| Chile | 0.00 | 0.15 | 0.62 | 1.45 | 3.51 | 2.18 | 2.11 | 1.38 | 108.27 | 82.59 | 70.77 | 55.59 |
| China | 0.01 | 0.22 | 0.92 | 3.29 | 11.61 | 9.95 | 6.52 | 4.51 | 126.93 | 134.68 | 83.92 | 70.87 |
| Colombia | 0.02 | 0.78 | 3.50 | 7.44 | 7.30 | 6.78 | 4.67 | 3.15 | 391.72 | 241.38 | 165.93 | 101.23 |
| Comoros | 0.01 | 0.56 | 2.41 | 5.74 | 85.48 | 90.98 | 63.72 | 43.20 | 1306.39 | 909.40 | 653.10 | 379.55 |
| Congo | 0.22 | 7.33 | 31.25 | 74.02 | 234.70 | 227.53 | 182.05 | 118.97 | 1216.26 | 629.98 | 470.41 | 486.01 |
| Cook Islands | 0.00 | 0.03 | 0.12 | 0.29 | 20.53 | 16.50 | 9.77 | 5.98 | 630.31 | 380.90 | 169.18 | 138.94 |
| Costa Rica | 0.01 | 0.40 | 1.72 | 4.03 | 6.54 | 6.25 | 5.19 | 3.10 | 384.20 | 220.22 | 146.56 | 89.29 |
| Croatia | 0.01 | 0.17 | 0.75 | 1.76 | 14.57 | 9.19 | 6.16 | 4.81 | 314.75 | 225.21 | 156.35 | 145.27 |
| Cuba | 0.01 | 0.45 | 2.02 | 4.79 | 7.68 | 5.13 | 5.15 | 3.19 | 572.77 | 411.34 | 273.12 | 260.31 |
| Cyprus | 0.01 | 0.34 | 1.44 | 3.69 | 0.96 | 0.69 | 0.55 | 0.18 | 102.34 | 120.70 | 75.67 | 31.09 |
| Czechia | 0.00 | 0.10 | 0.41 | 0.97 | 10.18 | 6.23 | 5.75 | 4.12 | 319.02 | 214.19 | 150.72 | 140.17 |
| Côte d'Ivoire | 0.03 | 1.13 | 4.81 | 10.90 | 138.93 | 136.33 | 94.43 | 65.92 | 1914.27 | 987.51 | 733.73 | 688.95 |
| Democratic People's Republic of Korea | 0.00 | 0.07 | 0.29 | 0.70 | 48.94 | 28.92 | 13.51 | 8.44 | 701.25 | 405.21 | 144.32 | 133.05 |
| Democratic Republic of the Congo | 0.66 | 20.10 | 62.88 | 124.73 | 167.44 | 192.66 | 92.73 | 102.12 | 1149.53 | 527.62 | 326.81 | 435.61 |
| Denmark | 0.01 | 0.30 | 1.26 | 3.17 | 0.45 | 0.33 | 0.27 | 0.09 | 108.27 | 122.63 | 82.11 | 33.20 |
| Djibouti | 0.12 | 4.72 | 16.95 | 40.51 | 73.67 | 86.92 | 53.16 | 33.36 | 1292.72 | 902.46 | 610.77 | 352.93 |
| Dominica | 0.01 | 0.40 | 1.76 | 4.18 | 11.41 | 15.48 | 14.83 | 11.39 | 875.57 | 703.66 | 484.47 | 426.20 |
| Dominican Republic | 0.01 | 0.43 | 2.01 | 4.64 | 23.45 | 23.90 | 21.95 | 16.46 | 811.39 | 662.12 | 443.73 | 394.37 |
| Ecuador | 0.00 | 0.06 | 0.23 | 0.56 | 17.89 | 11.57 | 7.90 | 5.21 | 556.15 | 88.61 | 41.94 | 19.91 |
| Egypt | 0.02 | 0.74 | 3.15 | 7.20 | 11.66 | 13.92 | 11.78 | 8.74 | 658.74 | 382.27 | 288.67 | 216.35 |
| El Salvador | 0.02 | 0.90 | 3.98 | 8.63 | 25.65 | 12.81 | 10.35 | 5.46 | 644.69 | 183.62 | 163.75 | 113.35 |
| Equatorial Guinea | 0.14 | 5.14 | 18.62 | 40.19 | 57.87 | 68.29 | 41.66 | 34.12 | 1344.37 | 784.32 | 323.11 | 340.57 |
| Eritrea | 0.02 | 0.68 | 2.91 | 6.92 | 99.52 | 159.65 | 93.77 | 62.40 | 1505.85 | 1115.91 | 828.27 | 473.38 |
| Estonia | 0.00 | 0.15 | 0.73 | 1.70 | 0.57 | 0.39 | 0.29 | 0.25 | 303.58 | 201.17 | 150.11 | 149.13 |
| Eswatini | 0.02 | 0.55 | 2.32 | 5.62 | 78.46 | 55.72 | 54.65 | 21.65 | 1359.13 | 565.09 | 520.33 | 334.03 |
| Ethiopia | 0.13 | 4.54 | 17.98 | 41.93 | 114.51 | 120.40 | 86.53 | 40.76 | 1675.50 | 1000.89 | 742.63 | 371.02 |
| Fiji | 0.00 | 0.03 | 0.13 | 0.33 | 47.16 | 37.05 | 34.48 | 16.03 | 1207.06 | 780.71 | 362.41 | 263.04 |
| Finland | 0.01 | 0.32 | 1.40 | 3.59 | 0.64 | 0.54 | 0.41 | 0.12 | 99.84 | 120.15 | 81.59 | 33.93 |
| France | 0.01 | 0.33 | 1.40 | 3.59 | 0.26 | 0.20 | 0.13 | 0.04 | 113.40 | 133.92 | 89.07 | 36.61 |
| Gabon | 0.08 | 3.34 | 12.12 | 25.39 | 57.00 | 58.82 | 56.84 | 39.95 | 1186.02 | 747.53 | 606.05 | 755.07 |
| Gambia | 0.06 | 2.31 | 9.78 | 22.44 | 177.59 | 181.34 | 156.75 | 101.09 | 1884.57 | 1522.63 | 1502.46 | 845.96 |
| Georgia | 0.01 | 0.30 | 1.29 | 2.93 | 7.61 | 6.14 | 5.15 | 2.71 | 948.98 | 763.36 | 469.75 | 315.75 |
| Germany | 0.01 | 0.33 | 1.39 | 3.53 | 0.26 | 0.20 | 0.18 | 0.06 | 102.69 | 118.58 | 75.99 | 31.66 |
| Ghana | 0.04 | 1.65 | 6.91 | 15.42 | 151.89 | 180.21 | 137.25 | 74.13 | 1602.83 | 1042.97 | 812.97 | 563.22 |
| Greece | 0.01 | 0.36 | 1.54 | 3.96 | 1.65 | 1.24 | 1.02 | 0.33 | 130.00 | 154.43 | 96.56 | 39.62 |
| Greenland | 0.00 | 0.17 | 0.72 | 1.69 | 0.61 | 0.39 | 0.37 | 0.25 | 64.67 | 44.86 | 41.28 | 35.78 |
| Grenada | 0.01 | 0.33 | 1.47 | 3.48 | 21.95 | 22.22 | 19.94 | 13.67 | 887.67 | 627.56 | 443.03 | 394.07 |
| Guam | 0.00 | 0.03 | 0.12 | 0.28 | 16.16 | 11.75 | 6.43 | 3.46 | 708.71 | 424.94 | 193.25 | 150.90 |
| Guatemala | 0.02 | 0.89 | 3.90 | 8.48 | 24.46 | 18.37 | 10.79 | 7.33 | 1056.76 | 335.02 | 292.80 | 269.19 |
| Guinea | 0.05 | 2.06 | 8.47 | 20.13 | 153.88 | 216.90 | 140.72 | 96.41 | 1632.48 | 1218.16 | 798.89 | 632.48 |
| Guinea-Bissau | 0.06 | 2.36 | 9.95 | 22.99 | 182.74 | 188.94 | 157.45 | 101.36 | 1531.60 | 1262.79 | 1130.54 | 930.46 |
| Guyana | 0.02 | 0.62 | 2.72 | 6.49 | 24.04 | 24.27 | 24.91 | 17.65 | 938.61 | 485.14 | 410.43 | 415.01 |
| Haiti | 0.03 | 1.18 | 5.10 | 12.19 | 105.74 | 117.33 | 117.49 | 73.56 | 1709.76 | 1096.74 | 1387.76 | 892.93 |
| Honduras | 0.03 | 1.17 | 5.05 | 11.03 | 22.26 | 15.09 | 11.77 | 6.44 | 905.35 | 342.47 | 301.52 | 205.54 |
| Hungary | 0.01 | 0.20 | 0.82 | 1.89 | 17.73 | 11.37 | 8.46 | 5.55 | 373.03 | 256.93 | 175.39 | 159.79 |
| Iceland | 0.00 | 0.16 | 0.71 | 1.72 | 0.49 | 0.37 | 0.30 | 0.09 | 91.78 | 104.14 | 71.06 | 29.05 |
| India | 0.08 | 2.75 | 11.26 | 31.13 | 100.98 | 122.84 | 63.69 | 38.69 | 1768.74 | 1195.02 | 1138.47 | 757.50 |
| Indonesia | 0.00 | 0.11 | 0.47 | 1.34 | 55.59 | 31.96 | 22.17 | 15.67 | 684.80 | 334.41 | 351.52 | 153.61 |
| Iran (Islamic Republic of) | 0.02 | 0.59 | 2.49 | 5.62 | 6.75 | 5.57 | 3.33 | 1.71 | 670.19 | 475.25 | 289.92 | 196.82 |
| Iraq | 0.07 | 2.32 | 9.74 | 21.76 | 20.92 | 24.74 | 16.92 | 11.35 | 801.37 | 556.56 | 335.74 | 233.98 |
| Ireland | 0.01 | 0.33 | 1.39 | 3.52 | 0.48 | 0.39 | 0.27 | 0.08 | 90.63 | 106.46 | 72.39 | 29.01 |
| Israel | 0.01 | 0.33 | 1.42 | 3.67 | 7.10 | 5.28 | 4.33 | 1.39 | 133.73 | 155.14 | 100.39 | 41.54 |
| Italy | 0.02 | 0.77 | 3.27 | 9.51 | 1.43 | 1.04 | 0.84 | 0.26 | 108.00 | 110.48 | 68.71 | 26.55 |
| Jamaica | 0.01 | 0.46 | 2.07 | 4.85 | 21.99 | 21.60 | 18.94 | 13.62 | 902.76 | 565.31 | 367.43 | 332.10 |
| Japan | 0.01 | 0.20 | 0.86 | 2.03 | 0.75 | 0.38 | 0.22 | 0.16 | 113.55 | 43.78 | 31.66 | 23.55 |
| Jordan | 0.04 | 1.22 | 5.24 | 11.83 | 18.26 | 23.60 | 22.11 | 14.36 | 669.79 | 432.85 | 323.56 | 307.04 |
| Kazakhstan | 0.01 | 0.36 | 1.53 | 3.58 | 40.33 | 42.08 | 43.18 | 15.59 | 764.19 | 635.56 | 502.53 | 358.79 |
| Kenya | 0.02 | 0.60 | 2.55 | 6.33 | 151.20 | 127.30 | 76.77 | 36.36 | 1050.84 | 383.37 | 271.08 | 135.45 |
| Kiribati | 0.00 | 0.09 | 0.40 | 0.97 | 162.93 | 147.99 | 95.82 | 53.66 | 1491.65 | 1061.64 | 552.25 | 397.50 |
| Kuwait | 0.01 | 0.40 | 1.65 | 3.81 | 6.25 | 12.11 | 11.43 | 8.84 | 488.84 | 352.87 | 223.71 | 160.76 |
| Kyrgyzstan | 0.01 | 0.28 | 1.16 | 2.66 | 20.87 | 24.88 | 22.91 | 13.89 | 980.82 | 690.26 | 554.63 | 451.67 |
| Lao People's Democratic Republic | 0.00 | 0.10 | 0.42 | 1.22 | 78.34 | 69.77 | 40.07 | 29.30 | 1042.16 | 418.06 | 316.06 | 396.03 |
| Latvia | 0.00 | 0.14 | 0.70 | 1.66 | 0.76 | 0.50 | 0.40 | 0.35 | 367.71 | 236.72 | 174.45 | 172.00 |
| Lebanon | 0.04 | 1.25 | 5.33 | 12.30 | 6.12 | 4.63 | 3.55 | 1.80 | 645.60 | 461.05 | 282.25 | 206.04 |
| Lesotho | 0.06 | 2.69 | 10.84 | 23.21 | 91.15 | 105.83 | 76.54 | 33.43 | 1032.17 | 836.06 | 576.24 | 429.80 |
| Liberia | 0.02 | 0.86 | 3.75 | 8.75 | 98.94 | 132.36 | 89.32 | 53.31 | 1400.91 | 979.93 | 687.04 | 895.35 |
| Libya | 0.07 | 2.29 | 9.57 | 21.29 | 13.94 | 19.87 | 15.80 | 11.95 | 756.33 | 543.74 | 319.18 | 213.26 |
| Lithuania | 0.00 | 0.14 | 0.70 | 1.63 | 0.56 | 0.39 | 0.30 | 0.26 | 364.51 | 235.09 | 173.12 | 169.59 |
| Luxembourg | 0.01 | 0.32 | 1.35 | 3.43 | 0.46 | 0.35 | 0.27 | 0.08 | 88.92 | 99.32 | 68.73 | 28.00 |
| Madagascar | 0.05 | 1.84 | 7.77 | 19.04 | 110.95 | 114.07 | 64.90 | 47.25 | 1259.54 | 566.09 | 486.27 | 491.37 |
| Malawi | 0.06 | 2.00 | 8.25 | 20.91 | 131.54 | 143.74 | 111.96 | 101.57 | 1941.40 | 999.14 | 932.14 | 623.69 |
| Malaysia | 0.01 | 0.28 | 1.23 | 4.08 | 4.10 | 2.62 | 0.88 | 0.71 | 786.12 | 487.70 | 217.93 | 191.25 |
| Maldives | 0.00 | 0.08 | 0.34 | 0.93 | 31.55 | 24.01 | 18.51 | 10.43 | 928.37 | 592.51 | 361.97 | 419.84 |
| Mali | 0.02 | 0.95 | 4.16 | 9.55 | 253.59 | 288.15 | 224.29 | 173.09 | 2193.13 | 2026.92 | 2120.34 | 1290.62 |
| Malta | 0.01 | 0.33 | 1.44 | 3.69 | 1.38 | 1.04 | 0.81 | 0.26 | 121.20 | 141.76 | 92.56 | 38.77 |
| Marshall Islands | 0.00 | 0.08 | 0.33 | 0.81 | 200.51 | 151.10 | 75.97 | 37.87 | 1604.35 | 873.45 | 423.62 | 328.79 |
| Mauritania | 0.06 | 2.18 | 9.06 | 19.73 | 74.04 | 79.26 | 70.93 | 36.63 | 1374.74 | 1082.32 | 876.65 | 667.77 |
| Mauritius | 0.00 | 0.09 | 0.42 | 1.20 | 23.40 | 17.63 | 9.60 | 4.90 | 1004.11 | 496.14 | 194.98 | 164.94 |
| Mexico | 0.02 | 0.79 | 3.34 | 7.50 | 18.79 | 19.21 | 13.55 | 6.02 | 331.74 | 248.78 | 179.73 | 71.81 |
| Micronesia (Federated States of) | 0.00 | 0.08 | 0.34 | 0.82 | 193.93 | 183.99 | 96.79 | 53.83 | 1218.85 | 784.35 | 354.75 | 281.25 |
| Monaco | 0.01 | 0.32 | 1.35 | 3.33 | 0.24 | 0.18 | 0.13 | 0.04 | 60.42 | 58.42 | 41.51 | 17.63 |
| Mongolia | 0.02 | 0.67 | 2.97 | 6.40 | 10.43 | 14.66 | 11.58 | 7.92 | 1195.53 | 1065.10 | 747.54 | 548.05 |
| Montenegro | 0.01 | 0.19 | 0.79 | 1.83 | 17.23 | 10.67 | 8.89 | 6.20 | 394.02 | 268.07 | 181.78 | 164.91 |
| Morocco | 0.09 | 3.02 | 12.73 | 27.98 | 32.96 | 35.41 | 27.81 | 17.51 | 940.42 | 666.93 | 418.02 | 271.07 |
| Mozambique | 0.03 | 1.14 | 4.85 | 11.56 | 164.55 | 176.09 | 117.60 | 76.78 | 1778.86 | 857.84 | 629.38 | 1064.35 |
| Myanmar | 0.01 | 0.23 | 0.98 | 2.91 | 53.56 | 55.62 | 55.83 | 22.47 | 1395.72 | 675.41 | 782.35 | 516.55 |
| Namibia | 0.01 | 0.60 | 2.79 | 6.12 | 43.77 | 47.67 | 41.96 | 21.45 | 1370.57 | 669.87 | 567.77 | 327.38 |
| Nauru | 0.00 | 0.03 | 0.13 | 0.32 | 80.47 | 67.15 | 45.06 | 23.14 | 1238.75 | 791.70 | 387.41 | 280.38 |
| Nepal | 0.02 | 0.58 | 2.49 | 6.31 | 34.78 | 32.42 | 31.98 | 23.71 | 1493.04 | 721.40 | 639.29 | 520.44 |
| Netherlands | 0.01 | 0.32 | 1.36 | 3.45 | 0.43 | 0.35 | 0.26 | 0.08 | 97.85 | 121.24 | 80.65 | 32.85 |
| New Zealand | 0.01 | 0.18 | 0.77 | 1.83 | 0.64 | 0.27 | 0.25 | 0.09 | 252.51 | 64.53 | 57.17 | 25.14 |
| Nicaragua | 0.03 | 1.13 | 4.93 | 10.59 | 6.56 | 5.43 | 4.82 | 3.11 | 574.02 | 339.29 | 217.30 | 140.46 |
| Niger | 0.06 | 2.28 | 9.49 | 23.62 | 396.19 | 424.13 | 282.43 | 211.75 | 1548.10 | 1115.65 | 837.57 | 581.75 |
| Nigeria | 0.02 | 0.58 | 2.47 | 6.16 | 48.87 | 68.89 | 33.44 | 30.60 | 1864.79 | 1179.02 | 1046.85 | 836.08 |
| Niue | 0.00 | 0.03 | 0.13 | 0.32 | 38.50 | 31.03 | 19.28 | 10.41 | 973.26 | 598.57 | 265.62 | 204.45 |
| North Macedonia | 0.01 | 0.19 | 0.81 | 1.88 | 40.13 | 22.17 | 20.23 | 12.63 | 474.10 | 348.58 | 229.25 | 205.29 |
| Northern Mariana Islands | 0.00 | 0.03 | 0.12 | 0.29 | 22.12 | 16.17 | 9.58 | 4.89 | 756.79 | 459.55 | 205.51 | 164.36 |
| Norway | 0.01 | 0.34 | 1.46 | 3.69 | 0.30 | 0.24 | 0.17 | 0.05 | 102.99 | 99.95 | 65.67 | 27.15 |
| Oman | 0.03 | 0.88 | 3.79 | 8.46 | 25.52 | 23.14 | 20.50 | 16.03 | 722.45 | 389.07 | 219.70 | 140.28 |
| Pakistan | 0.11 | 3.53 | 14.18 | 36.59 | 31.30 | 42.35 | 26.90 | 22.95 | 1801.51 | 1939.39 | 1585.95 | 1209.29 |
| Palau | 0.00 | 0.03 | 0.13 | 0.31 | 31.61 | 25.96 | 16.37 | 8.96 | 982.11 | 597.23 | 269.49 | 208.35 |
| Palestine | 0.01 | 0.42 | 1.85 | 4.21 | 19.78 | 21.01 | 20.81 | 14.86 | 534.91 | 299.66 | 235.63 | 192.40 |
| Panama | 0.01 | 0.26 | 1.11 | 2.59 | 8.92 | 8.62 | 5.61 | 3.49 | 454.80 | 239.87 | 159.60 | 103.99 |
| Papua New Guinea | 0.00 | 0.08 | 0.33 | 0.81 | 74.50 | 76.01 | 36.68 | 20.18 | 1358.97 | 803.47 | 370.95 | 244.26 |
| Paraguay | 0.00 | 0.09 | 0.39 | 0.91 | 25.34 | 22.31 | 20.57 | 14.55 | 743.12 | 444.15 | 248.75 | 211.83 |
| Peru | 0.00 | 0.06 | 0.23 | 0.56 | 25.20 | 21.12 | 22.72 | 12.38 | 900.95 | 327.58 | 261.53 | 175.18 |
| Philippines | 0.01 | 0.51 | 2.19 | 8.81 | 48.91 | 41.13 | 21.28 | 11.56 | 946.07 | 686.04 | 292.60 | 269.39 |
| Poland | 0.01 | 0.18 | 0.75 | 1.77 | 16.36 | 9.94 | 8.71 | 5.67 | 432.10 | 276.93 | 194.20 | 163.20 |
| Portugal | 0.01 | 0.41 | 1.77 | 4.56 | 2.33 | 1.67 | 1.39 | 0.43 | 128.32 | 146.66 | 101.20 | 41.11 |
| Puerto Rico | 0.00 | 0.15 | 0.78 | 1.85 | 2.61 | 1.86 | 1.88 | 1.16 | 422.68 | 302.64 | 214.34 | 195.86 |
| Qatar | 0.01 | 0.36 | 1.53 | 3.50 | 1.56 | 1.37 | 0.97 | 0.63 | 406.24 | 298.44 | 193.56 | 131.83 |
| Republic of Korea | 0.01 | 0.18 | 0.79 | 1.85 | 0.37 | 0.18 | 0.14 | 0.10 | 70.19 | 32.76 | 29.49 | 25.21 |
| Republic of Moldova | 0.00 | 0.18 | 0.77 | 1.79 | 2.07 | 1.19 | 1.49 | 1.02 | 450.07 | 220.47 | 203.89 | 199.18 |
| Romania | 0.01 | 0.44 | 1.86 | 4.33 | 24.01 | 15.70 | 11.74 | 7.93 | 460.11 | 316.30 | 206.28 | 182.30 |
| Russian Federation | 0.01 | 0.32 | 1.49 | 3.41 | 0.25 | 0.20 | 0.10 | 0.08 | 385.70 | 250.51 | 182.86 | 180.15 |
| Rwanda | 0.03 | 1.03 | 4.32 | 11.61 | 64.16 | 63.71 | 38.44 | 23.17 | 1083.83 | 436.95 | 408.92 | 264.87 |
| Saint Kitts and Nevis | 0.01 | 0.22 | 1.04 | 2.43 | 11.68 | 16.34 | 16.33 | 11.41 | 761.34 | 567.28 | 383.40 | 334.34 |
| Saint Lucia | 0.00 | 0.20 | 0.89 | 2.12 | 17.05 | 19.57 | 20.88 | 13.93 | 806.82 | 607.48 | 419.66 | 379.03 |
| Saint Vincent and the Grenadines | 0.01 | 0.36 | 1.66 | 3.94 | 23.59 | 24.69 | 25.03 | 16.61 | 890.39 | 663.98 | 458.36 | 401.47 |
| Samoa | 0.00 | 0.03 | 0.11 | 0.26 | 80.55 | 58.92 | 57.10 | 25.03 | 1025.70 | 685.49 | 315.29 | 238.74 |
| San Marino | 0.01 | 0.33 | 1.38 | 3.54 | 0.45 | 0.35 | 0.27 | 0.08 | 95.80 | 112.27 | 74.55 | 30.98 |
| Sao Tome and Principe | 0.02 | 0.90 | 3.95 | 9.02 | 107.14 | 82.42 | 85.51 | 46.65 | 1479.10 | 758.60 | 816.49 | 666.13 |
| Saudi Arabia | 0.01 | 0.34 | 1.50 | 3.37 | 1.09 | 0.90 | 0.48 | 0.24 | 534.64 | 388.22 | 243.63 | 156.10 |
| Senegal | 0.04 | 1.50 | 6.35 | 14.46 | 44.50 | 56.92 | 68.71 | 54.87 | 1697.47 | 1320.91 | 1772.39 | 1087.82 |
| Serbia | 0.00 | 0.15 | 0.62 | 1.47 | 39.06 | 25.85 | 20.01 | 13.25 | 406.16 | 277.96 | 185.61 | 165.56 |
| Seychelles | 0.00 | 0.09 | 0.40 | 1.12 | 13.17 | 10.38 | 7.12 | 4.71 | 761.19 | 469.14 | 211.37 | 197.80 |
| Sierra Leone | 0.04 | 1.32 | 5.64 | 13.51 | 174.91 | 204.18 | 116.49 | 96.95 | 1640.12 | 1274.97 | 720.34 | 600.22 |
| Singapore | 0.01 | 0.19 | 0.79 | 1.90 | 0.61 | 0.30 | 0.21 | 0.20 | 72.21 | 31.79 | 26.04 | 20.05 |
| Slovakia | 0.01 | 0.17 | 0.74 | 1.73 | 15.79 | 9.82 | 7.75 | 5.41 | 387.83 | 269.29 | 178.54 | 161.44 |
| Slovenia | 0.01 | 0.17 | 0.73 | 1.71 | 8.26 | 5.10 | 4.11 | 3.09 | 271.05 | 183.05 | 130.78 | 120.32 |
| Solomon Islands | 0.00 | 0.08 | 0.32 | 0.79 | 175.73 | 163.72 | 124.00 | 64.88 | 1290.62 | 831.14 | 380.13 | 277.08 |
| Somalia | 0.28 | 8.09 | 32.22 | 80.17 | 506.81 | 381.47 | 386.40 | 278.63 | 1535.60 | 980.48 | 603.21 | 328.83 |
| South Africa | 0.01 | 0.50 | 2.18 | 5.07 | 36.92 | 37.86 | 29.25 | 19.08 | 1424.84 | 1381.79 | 1105.55 | 688.21 |
| South Sudan | 0.04 | 1.54 | 6.49 | 15.58 | 204.10 | 234.85 | 155.21 | 107.73 | 1375.42 | 881.92 | 470.82 | 244.60 |
| Spain | 0.01 | 0.33 | 1.43 | 3.57 | 0.56 | 0.54 | 0.45 | 0.17 | 107.42 | 129.20 | 83.36 | 32.96 |
| Sri Lanka | 0.00 | 0.15 | 0.61 | 2.06 | 19.76 | 15.15 | 10.33 | 7.19 | 671.09 | 481.71 | 194.71 | 201.13 |
| Sudan | 0.08 | 3.14 | 13.44 | 28.33 | 43.64 | 76.92 | 36.71 | 23.07 | 1106.08 | 807.73 | 715.03 | 476.44 |
| Suriname | 0.01 | 0.33 | 1.51 | 3.54 | 28.09 | 29.54 | 30.39 | 20.72 | 1007.67 | 751.55 | 496.04 | 413.20 |
| Sweden | 0.01 | 0.35 | 1.51 | 3.81 | 0.47 | 0.37 | 0.28 | 0.08 | 108.75 | 113.70 | 76.99 | 30.51 |
| Switzerland | 0.01 | 0.22 | 0.97 | 2.37 | 0.21 | 0.17 | 0.15 | 0.04 | 94.28 | 106.94 | 66.85 | 25.93 |
| Syrian Arab Republic | 0.08 | 2.56 | 11.09 | 24.51 | 25.25 | 23.35 | 9.22 | 5.17 | 801.19 | 570.58 | 348.27 | 234.56 |
| Taiwan (Province of China) | 0.00 | 0.04 | 0.17 | 0.44 | 1.11 | 0.61 | 0.34 | 0.23 | 287.40 | 156.45 | 68.52 | 57.93 |
| Tajikistan | 0.03 | 1.21 | 5.08 | 11.31 | 48.78 | 47.03 | 35.22 | 23.76 | 1288.40 | 1153.27 | 760.14 | 583.57 |
| Thailand | 0.01 | 0.23 | 1.01 | 2.95 | 11.01 | 16.12 | 8.32 | 6.10 | 543.62 | 344.71 | 143.56 | 149.11 |
| Timor-Leste | 0.01 | 0.22 | 0.94 | 2.69 | 159.07 | 137.69 | 39.48 | 21.01 | 1102.12 | 366.55 | 183.75 | 164.47 |
| Togo | 0.05 | 1.80 | 7.50 | 17.91 | 160.05 | 142.45 | 128.62 | 109.20 | 1997.34 | 1296.94 | 2219.97 | 967.18 |
| Tokelau | 0.00 | 0.07 | 0.29 | 0.69 | 51.49 | 42.64 | 26.16 | 13.88 | 1031.82 | 645.65 | 289.10 | 220.69 |
| Tonga | 0.00 | 0.07 | 0.31 | 0.74 | 64.02 | 46.45 | 30.66 | 17.00 | 1002.27 | 653.99 | 307.93 | 236.28 |
| Trinidad and Tobago | 0.01 | 0.33 | 1.68 | 3.85 | 10.14 | 14.76 | 14.49 | 10.14 | 743.82 | 540.18 | 368.67 | 340.10 |
| Tunisia | 0.02 | 0.77 | 3.30 | 7.41 | 9.18 | 13.32 | 10.79 | 8.27 | 643.92 | 489.78 | 305.14 | 199.46 |
| Türkiye | 0.06 | 2.18 | 9.26 | 19.72 | 10.02 | 9.01 | 5.74 | 3.49 | 591.84 | 412.13 | 252.93 | 171.61 |
| Turkmenistan | 0.01 | 0.30 | 1.29 | 2.92 | 20.53 | 21.30 | 17.73 | 12.10 | 1093.40 | 960.84 | 645.56 | 476.41 |
| Tuvalu | 0.00 | 0.08 | 0.33 | 0.79 | 107.98 | 91.96 | 59.62 | 29.63 | 1236.12 | 791.22 | 371.77 | 272.77 |
| Uganda | 0.01 | 0.50 | 2.09 | 5.21 | 55.80 | 67.12 | 50.95 | 24.97 | 1140.84 | 606.07 | 341.29 | 279.47 |
| Ukraine | 0.03 | 1.17 | 5.13 | 10.73 | 2.22 | 1.42 | 0.86 | 0.65 | 460.46 | 313.00 | 213.65 | 211.31 |
| United Arab Emirates | 0.03 | 1.04 | 4.40 | 10.14 | 2.32 | 2.13 | 1.47 | 1.14 | 628.37 | 450.06 | 271.42 | 169.34 |
| United Kingdom | 0.01 | 0.34 | 1.45 | 3.68 | 1.07 | 0.80 | 0.60 | 0.16 | 273.12 | 246.99 | 139.10 | 41.81 |
| United Republic of Tanzania | 0.01 | 0.59 | 2.52 | 6.06 | 65.97 | 77.72 | 60.28 | 45.97 | 1658.81 | 810.00 | 751.61 | 520.81 |
| United States of America | 0.01 | 0.18 | 0.75 | 1.78 | 0.28 | 0.19 | 0.23 | 0.13 | 46.51 | 29.20 | 35.29 | 28.02 |
| United States Virgin Islands | 0.00 | 0.14 | 0.70 | 1.66 | 3.66 | 2.48 | 2.53 | 1.60 | 591.54 | 414.22 | 283.96 | 257.27 |
| Uruguay | 0.00 | 0.15 | 0.64 | 1.45 | 20.84 | 10.37 | 4.13 | 2.40 | 340.66 | 96.75 | 53.53 | 51.88 |
| Uzbekistan | 0.03 | 1.05 | 4.42 | 9.64 | 19.88 | 26.37 | 23.37 | 13.75 | 1430.67 | 1085.90 | 859.19 | 738.40 |
| Vanuatu | 0.01 | 0.28 | 1.18 | 3.44 | 150.78 | 137.28 | 75.61 | 48.53 | 1308.26 | 918.29 | 466.09 | 333.37 |
| Venezuela (Bolivarian Republic of) | 0.02 | 0.73 | 3.36 | 7.21 | 22.18 | 17.90 | 12.25 | 5.69 | 523.46 | 286.39 | 203.38 | 121.20 |
| Viet Nam | 0.01 | 0.26 | 1.15 | 3.66 | 20.15 | 19.69 | 7.89 | 4.86 | 684.30 | 482.07 | 212.45 | 190.50 |
| Yemen | 0.21 | 6.15 | 25.16 | 53.25 | 134.77 | 181.19 | 46.53 | 35.57 | 3480.34 | 2788.54 | 2011.13 | 1347.67 |
| Zambia | 0.03 | 1.12 | 4.83 | 11.04 | 71.74 | 113.99 | 69.13 | 80.56 | 1270.79 | 794.16 | 1314.05 | 1288.64 |
| Zimbabwe | 0.04 | 1.20 | 5.08 | 13.04 | 118.84 | 106.47 | 102.06 | 57.17 | 1609.22 | 928.69 | 812.57 | 540.65 |

STable 2. Age-specific incidence rate of nutritional deficiency in 0-14 years at the national level in 2021.

|  | Iodine deficiency | Iodine deficiency | Iodine deficiency | Iodine deficiency | Vitamin A deficiency | Vitamin A deficiency | Vitamin A deficiency | Vitamin A deficiency |
| --- | --- | --- | --- | --- | --- | --- | --- | --- |
| incidence | <1 year | 2-4 years | 5-9 years | 10-14 years | <1 year | 2-4 years | 5-9 years | 10-14 years |
| Afghanistan | 10.37 | 72.98 | 155.70 | 161.03 | 33251.37 | 51832.50 | 21026.59 | 20210.50 |
| Albania | 1.36 | 9.46 | 20.24 | 22.61 | 19935.80 | 17347.23 | 10952.84 | 11136.96 |
| Algeria | 4.17 | 29.14 | 62.35 | 66.13 | 3582.70 | 4116.27 | 2839.97 | 2643.34 |
| American Samoa | 0.19 | 1.36 | 2.91 | 3.82 | 7858.13 | 9145.29 | 8129.88 | 7566.23 |
| Andorra | 2.33 | 16.32 | 35.13 | 54.41 | 708.48 | 635.89 | 512.12 | 396.80 |
| Angola | 35.73 | 251.07 | 529.29 | 625.83 | 22166.94 | 31273.48 | 15567.60 | 15307.75 |
| Antigua and Barbuda | 0.94 | 6.59 | 14.13 | 17.38 | 1876.07 | 2017.49 | 1717.33 | 1739.12 |
| Argentina | 1.05 | 7.36 | 15.76 | 17.53 | 8685.27 | 9925.54 | 8109.76 | 7253.79 |
| Armenia | 6.80 | 47.24 | 100.08 | 103.63 | 496.31 | 456.17 | 367.09 | 380.04 |
| Australia | 1.14 | 8.02 | 17.18 | 20.68 | 15.64 | 15.68 | 16.33 | 17.66 |
| Austria | 2.37 | 16.59 | 35.51 | 55.81 | 889.15 | 787.38 | 638.99 | 506.36 |
| Azerbaijan | 1.75 | 12.19 | 26.01 | 28.30 | 2708.02 | 3029.96 | 2223.43 | 2203.12 |
| Bahamas | 0.87 | 6.06 | 13.00 | 15.92 | 1184.96 | 1262.60 | 1089.16 | 1098.13 |
| Bahrain | 2.97 | 20.70 | 44.32 | 47.11 | 1748.06 | 1689.81 | 1463.73 | 1381.92 |
| Bangladesh | 7.14 | 49.86 | 106.55 | 194.75 | 3281.83 | 4981.60 | 2915.81 | 2446.28 |
| Barbados | 2.11 | 14.74 | 31.53 | 40.81 | 2070.96 | 1993.45 | 1792.13 | 1809.09 |
| Belarus | 1.01 | 7.03 | 15.06 | 16.49 | 514.82 | 505.47 | 487.28 | 540.05 |
| Belgium | 2.33 | 16.31 | 34.91 | 54.59 | 861.72 | 753.58 | 627.84 | 497.43 |
| Belize | 0.60 | 4.20 | 9.00 | 10.89 | 5304.85 | 6139.85 | 4266.30 | 4301.19 |
| Benin | 3.63 | 25.58 | 54.77 | 75.59 | 34596.04 | 47317.92 | 26035.25 | 25566.50 |
| Bermuda | 0.66 | 4.64 | 9.94 | 12.04 | 981.36 | 1043.51 | 942.51 | 928.73 |
| Bhutan | 2.49 | 17.49 | 37.51 | 53.70 | 5414.04 | 6831.18 | 4108.48 | 4185.65 |
| Bolivia (Plurinational State of) | 0.45 | 3.13 | 6.71 | 8.16 | 5810.63 | 7360.79 | 5441.65 | 5884.47 |
| Bosnia and Herzegovina | 2.50 | 17.47 | 37.40 | 42.68 | 12129.63 | 11588.45 | 9773.31 | 9871.46 |
| Botswana | 1.65 | 11.56 | 24.77 | 30.34 | 12248.56 | 14774.73 | 9767.68 | 8831.89 |
| Brazil | 0.60 | 4.21 | 9.02 | 11.01 | 10648.45 | 11286.93 | 9935.00 | 9537.47 |
| Brunei Darussalam | 1.35 | 9.44 | 20.22 | 24.37 | 1578.81 | 1635.61 | 1301.43 | 1173.45 |
| Bulgaria | 1.13 | 7.90 | 16.92 | 19.32 | 9191.41 | 9195.98 | 7374.67 | 7593.37 |
| Burkina Faso | 3.68 | 26.01 | 55.69 | 76.50 | 31811.56 | 36758.08 | 25439.65 | 26151.95 |
| Burundi | 6.92 | 48.70 | 104.19 | 181.79 | 17639.00 | 29551.08 | 11191.49 | 12837.47 |
| Cabo Verde | 4.57 | 31.89 | 68.26 | 98.38 | 5766.11 | 6643.19 | 5599.83 | 5689.74 |
| Cambodia | 0.94 | 6.57 | 14.07 | 40.85 | 12319.19 | 17887.32 | 8978.22 | 8687.63 |
| Cameroon | 2.97 | 20.86 | 44.65 | 59.58 | 17835.98 | 23392.69 | 18345.51 | 22014.93 |
| Canada | 1.23 | 8.60 | 18.42 | 22.26 | 1400.07 | 1225.50 | 1029.32 | 859.37 |
| Central African Republic | 32.36 | 227.87 | 480.32 | 569.19 | 40584.64 | 56677.62 | 31415.19 | 30551.82 |
| Chad | 4.12 | 29.20 | 62.50 | 87.60 | 41852.73 | 62900.00 | 38203.37 | 38816.70 |
| Chile | 1.06 | 7.40 | 15.84 | 17.62 | 4602.61 | 4622.09 | 4404.63 | 4113.47 |
| China | 1.58 | 10.90 | 23.38 | 88.41 | 3291.42 | 2910.24 | 2792.12 | 2690.82 |
| Colombia | 3.14 | 21.95 | 46.97 | 53.02 | 2815.48 | 3195.49 | 2419.86 | 2434.81 |
| Comoros | 1.93 | 13.56 | 29.04 | 38.26 | 15678.22 | 19871.72 | 10711.25 | 12018.59 |
| Congo | 53.01 | 368.51 | 765.77 | 890.17 | 42534.75 | 49377.00 | 36174.58 | 35361.61 |
| Cook Islands | 0.21 | 1.45 | 3.11 | 4.10 | 4871.66 | 5494.38 | 5249.76 | 4833.59 |
| Costa Rica | 2.92 | 20.34 | 43.53 | 49.16 | 2387.42 | 2643.34 | 2305.45 | 2338.09 |
| Croatia | 1.29 | 8.97 | 19.22 | 21.56 | 7188.03 | 7033.17 | 5910.45 | 6055.78 |
| Cuba | 1.66 | 11.58 | 24.80 | 31.44 | 2338.95 | 2361.18 | 2155.60 | 2185.08 |
| Cyprus | 2.43 | 17.04 | 36.41 | 57.79 | 1135.61 | 984.09 | 803.87 | 645.15 |
| Czechia | 0.71 | 4.96 | 10.61 | 12.01 | 4749.32 | 4572.79 | 4317.89 | 4495.15 |
| Côte d'Ivoire | 4.01 | 28.14 | 59.96 | 86.70 | 19167.64 | 25392.05 | 15872.31 | 14716.56 |
| Democratic People's Republic of Korea | 0.23 | 1.58 | 3.37 | 4.79 | 11769.89 | 14204.83 | 7798.20 | 7729.02 |
| Democratic Republic of the Congo | 78.32 | 544.18 | 1114.52 | 1272.95 | 31016.37 | 45927.09 | 24586.49 | 24298.74 |
| Denmark | 2.13 | 14.95 | 32.00 | 49.24 | 496.36 | 427.34 | 348.92 | 278.13 |
| Djibouti | 17.55 | 122.31 | 258.54 | 737.40 | 13196.89 | 18309.51 | 9666.88 | 10343.14 |
| Dominica | 1.48 | 10.35 | 22.17 | 27.72 | 1939.42 | 2044.16 | 1750.94 | 1750.05 |
| Dominican Republic | 1.76 | 12.32 | 26.37 | 33.55 | 5074.41 | 5425.29 | 4450.15 | 4442.38 |
| Ecuador | 0.40 | 2.81 | 6.02 | 7.32 | 4613.04 | 6272.13 | 3891.12 | 3862.36 |
| Egypt | 5.38 | 37.51 | 79.82 | 83.83 | 2755.49 | 2915.65 | 1913.97 | 1911.71 |
| El Salvador | 3.40 | 23.76 | 50.86 | 57.49 | 5829.68 | 7190.36 | 5341.16 | 5301.43 |
| Equatorial Guinea | 28.16 | 193.89 | 400.58 | 464.54 | 8292.33 | 10162.44 | 6032.37 | 5818.12 |
| Eritrea | 2.31 | 16.23 | 34.72 | 46.29 | 17732.34 | 33027.14 | 14943.90 | 16279.07 |
| Estonia | 0.70 | 4.87 | 10.44 | 11.47 | 275.61 | 300.22 | 265.91 | 284.48 |
| Eswatini | 3.95 | 27.65 | 59.02 | 76.39 | 12981.82 | 16478.62 | 9719.75 | 9288.90 |
| Ethiopia | 15.58 | 109.25 | 232.02 | 629.94 | 17534.52 | 27301.39 | 13797.33 | 13906.41 |
| Fiji | 0.23 | 1.61 | 3.45 | 4.60 | 8530.94 | 9269.83 | 8890.48 | 8177.89 |
| Finland | 2.37 | 16.57 | 35.46 | 55.58 | 811.42 | 713.83 | 584.81 | 455.89 |
| France | 2.38 | 16.67 | 35.67 | 56.03 | 299.59 | 261.47 | 195.43 | 150.28 |
| Gabon | 15.28 | 107.55 | 230.41 | 274.41 | 8717.64 | 10078.75 | 6960.89 | 6744.55 |
| Gambia | 8.01 | 56.24 | 120.17 | 218.38 | 26444.94 | 32051.92 | 22051.70 | 22108.73 |
| Georgia | 2.20 | 15.26 | 32.50 | 35.05 | 2011.99 | 2137.48 | 1671.22 | 1676.79 |
| Germany | 2.36 | 16.50 | 35.29 | 55.23 | 300.33 | 274.91 | 229.66 | 194.33 |
| Ghana | 6.08 | 42.64 | 91.13 | 144.57 | 24895.28 | 29993.88 | 20823.85 | 20635.38 |
| Greece | 2.60 | 18.16 | 38.88 | 63.01 | 1685.33 | 1490.38 | 1207.02 | 963.54 |
| Greenland | 1.21 | 8.46 | 18.14 | 21.90 | 1095.56 | 1072.74 | 942.79 | 798.97 |
| Grenada | 1.24 | 8.67 | 18.57 | 23.02 | 3587.88 | 3810.39 | 3170.40 | 3230.89 |
| Guam | 0.20 | 1.43 | 3.06 | 4.04 | 3890.48 | 4493.81 | 4115.03 | 3785.78 |
| Guatemala | 3.30 | 23.10 | 49.43 | 55.94 | 4928.94 | 7818.29 | 3186.24 | 3298.27 |
| Guinea | 7.12 | 50.15 | 107.12 | 183.36 | 26863.68 | 39220.00 | 23444.51 | 23458.93 |
| Guinea-Bissau | 8.10 | 56.86 | 121.43 | 219.12 | 31836.10 | 37592.00 | 25300.97 | 25238.77 |
| Guyana | 2.20 | 15.40 | 33.00 | 43.05 | 4426.58 | 4758.01 | 3670.23 | 3731.79 |
| Haiti | 4.04 | 28.41 | 60.79 | 91.18 | 16468.60 | 19663.09 | 12625.26 | 12553.93 |
| Honduras | 4.11 | 28.78 | 61.54 | 69.62 | 4768.89 | 6139.32 | 4048.45 | 4140.36 |
| Hungary | 1.39 | 9.68 | 20.73 | 23.12 | 7360.61 | 7166.65 | 5959.69 | 6160.81 |
| Iceland | 1.19 | 8.33 | 17.88 | 24.46 | 655.83 | 569.08 | 466.02 | 370.67 |
| India | 10.70 | 74.52 | 158.63 | 340.89 | 13809.62 | 19993.95 | 7535.50 | 7495.77 |
| Indonesia | 0.76 | 5.29 | 11.30 | 23.90 | 11997.38 | 14242.77 | 6696.15 | 6581.94 |
| Iran (Islamic Republic of) | 1.99 | 13.79 | 29.49 | 32.07 | 2120.98 | 2243.00 | 1712.75 | 1564.09 |
| Iraq | 7.83 | 54.61 | 116.59 | 121.77 | 4285.03 | 5142.46 | 3170.90 | 2947.45 |
| Ireland | 2.35 | 16.38 | 35.07 | 54.79 | 652.82 | 581.73 | 472.01 | 366.63 |
| Israel | 2.41 | 16.91 | 36.20 | 57.06 | 7888.18 | 7043.60 | 5729.88 | 4607.75 |
| Italy | 5.54 | 38.66 | 82.58 | 194.96 | 1596.76 | 1427.08 | 1188.82 | 1001.62 |
| Jamaica | 1.70 | 11.85 | 25.37 | 32.12 | 2908.70 | 2951.82 | 2673.89 | 2734.68 |
| Japan | 1.46 | 10.18 | 21.80 | 26.59 | 732.67 | 750.51 | 635.38 | 600.55 |
| Jordan | 4.13 | 28.96 | 61.94 | 65.58 | 4903.96 | 6091.77 | 5424.10 | 4315.37 |
| Kazakhstan | 2.59 | 18.14 | 38.89 | 42.36 | 10075.70 | 11809.90 | 8839.63 | 7581.39 |
| Kenya | 4.33 | 30.29 | 64.71 | 94.08 | 33008.05 | 40768.18 | 24254.07 | 22687.47 |
| Kiribati | 0.31 | 2.17 | 4.66 | 6.43 | 31790.20 | 38764.63 | 29531.24 | 28171.77 |
| Kuwait | 2.84 | 19.82 | 42.39 | 45.25 | 684.65 | 691.91 | 613.28 | 579.35 |
| Kyrgyzstan | 1.95 | 13.63 | 29.20 | 31.92 | 4350.59 | 5634.59 | 3928.50 | 3846.03 |
| Lao People's Democratic Republic | 0.72 | 5.04 | 10.80 | 24.80 | 14806.49 | 25349.60 | 11939.10 | 11827.51 |
| Latvia | 0.74 | 5.12 | 10.98 | 12.12 | 324.22 | 350.85 | 303.66 | 334.39 |
| Lebanon | 9.09 | 63.25 | 134.76 | 139.86 | 1940.48 | 2122.09 | 1542.76 | 1465.40 |
| Lesotho | 9.93 | 69.80 | 148.85 | 252.31 | 21001.73 | 29041.67 | 15696.94 | 15063.21 |
| Liberia | 2.95 | 20.71 | 44.35 | 59.02 | 17991.81 | 25503.30 | 14875.64 | 14773.20 |
| Libya | 7.77 | 54.29 | 115.88 | 121.04 | 2599.65 | 3281.10 | 1949.44 | 1829.09 |
| Lithuania | 0.70 | 4.87 | 10.42 | 11.48 | 243.37 | 263.77 | 231.74 | 252.12 |
| Luxembourg | 2.29 | 16.09 | 34.34 | 53.55 | 587.60 | 520.88 | 408.71 | 330.21 |
| Madagascar | 6.26 | 43.97 | 94.10 | 159.09 | 21048.92 | 34297.03 | 13548.48 | 14844.60 |
| Malawi | 6.80 | 47.72 | 102.07 | 178.08 | 21515.54 | 27763.26 | 15557.11 | 16567.80 |
| Malaysia | 1.02 | 7.14 | 15.31 | 48.99 | 955.03 | 1107.07 | 593.76 | 618.86 |
| Maldives | 0.58 | 4.08 | 8.73 | 17.12 | 4770.62 | 5044.94 | 3417.11 | 3216.19 |
| Mali | 3.31 | 23.43 | 50.13 | 67.73 | 36009.52 | 46195.77 | 31255.56 | 31573.47 |
| Malta | 2.45 | 17.07 | 36.50 | 57.55 | 1567.20 | 1368.39 | 1102.56 | 876.05 |
| Marshall Islands | 0.26 | 1.83 | 3.93 | 5.32 | 28304.15 | 31447.86 | 24480.50 | 24160.99 |
| Mauritania | 7.93 | 55.55 | 118.66 | 214.40 | 12152.81 | 14533.17 | 10090.16 | 10280.28 |
| Mauritius | 0.71 | 4.99 | 10.69 | 24.28 | 2834.48 | 3140.92 | 2298.82 | 2176.58 |
| Mexico | 4.86 | 33.90 | 72.36 | 82.09 | 8345.67 | 10485.20 | 8048.69 | 6449.84 |
| Micronesia (Federated States of) | 0.27 | 1.88 | 4.03 | 5.46 | 40205.44 | 42798.44 | 37806.23 | 36801.32 |
| Monaco | 2.25 | 15.74 | 33.72 | 51.60 | 378.56 | 337.11 | 270.10 | 212.36 |
| Mongolia | 2.40 | 16.79 | 36.01 | 39.25 | 1709.55 | 2539.33 | 1520.77 | 1527.06 |
| Montenegro | 1.34 | 9.35 | 20.02 | 22.39 | 6791.30 | 6573.01 | 5668.70 | 5789.37 |
| Morocco | 10.16 | 70.83 | 150.98 | 156.40 | 7574.48 | 8816.61 | 6763.17 | 6425.10 |
| Mozambique | 3.81 | 26.86 | 57.52 | 82.39 | 28531.29 | 35742.06 | 21499.29 | 22209.33 |
| Myanmar | 0.78 | 5.46 | 11.71 | 28.75 | 9436.01 | 13720.99 | 7375.89 | 6987.00 |
| Namibia | 2.67 | 18.70 | 40.10 | 50.19 | 8899.80 | 11718.00 | 7617.01 | 7140.31 |
| Nauru | 0.22 | 1.56 | 3.35 | 4.45 | 14768.23 | 16964.35 | 14846.53 | 14198.59 |
| Nepal | 4.21 | 29.53 | 63.21 | 96.96 | 5526.12 | 7217.80 | 4487.08 | 4632.47 |
| Netherlands | 2.30 | 16.11 | 34.47 | 53.44 | 562.45 | 491.51 | 398.15 | 324.08 |
| New Zealand | 1.32 | 9.21 | 19.72 | 24.12 | 312.46 | 305.24 | 329.06 | 339.56 |
| Nicaragua | 4.07 | 28.44 | 60.82 | 68.64 | 1791.90 | 1580.49 | 1590.83 | 1640.81 |
| Niger | 7.66 | 54.09 | 115.49 | 204.00 | 60948.97 | 77746.99 | 53042.34 | 52563.91 |
| Nigeria | 4.17 | 29.46 | 63.13 | 90.69 | 5948.51 | 10079.80 | 4336.87 | 4602.29 |
| Niue | 0.22 | 1.54 | 3.31 | 4.35 | 7705.22 | 8696.74 | 8020.67 | 7596.86 |
| North Macedonia | 1.37 | 9.58 | 20.53 | 23.00 | 13879.84 | 13576.46 | 12119.16 | 12166.23 |
| Northern Mariana Islands | 0.21 | 1.45 | 3.10 | 4.10 | 5200.98 | 5988.94 | 5578.40 | 5212.22 |
| Norway | 2.47 | 17.28 | 37.00 | 58.03 | 347.04 | 306.43 | 252.23 | 197.98 |
| Oman | 3.06 | 21.31 | 45.60 | 48.65 | 1702.35 | 1814.78 | 1276.11 | 1211.50 |
| Pakistan | 10.55 | 73.71 | 156.00 | 389.46 | 4050.81 | 6123.27 | 2584.43 | 2617.08 |
| Palau | 0.22 | 1.50 | 3.23 | 4.31 | 6241.72 | 7169.29 | 6567.42 | 6147.49 |
| Palestine | 3.13 | 21.90 | 46.86 | 49.97 | 5097.29 | 5385.06 | 4198.65 | 3921.57 |
| Panama | 1.88 | 13.17 | 28.20 | 31.91 | 2639.98 | 3322.37 | 2219.96 | 2255.09 |
| Papua New Guinea | 0.26 | 1.86 | 3.99 | 5.39 | 11187.27 | 16954.66 | 7617.84 | 7571.03 |
| Paraguay | 0.66 | 4.63 | 9.93 | 11.99 | 7133.86 | 7085.94 | 6666.14 | 6586.55 |
| Peru | 0.40 | 2.78 | 5.96 | 7.23 | 6093.56 | 6530.72 | 5278.70 | 5318.51 |
| Philippines | 1.74 | 12.19 | 26.07 | 131.14 | 13212.03 | 17459.73 | 8746.43 | 8290.21 |
| Poland | 1.29 | 8.98 | 19.23 | 21.83 | 5874.93 | 5688.96 | 4843.70 | 4953.64 |
| Portugal | 2.99 | 20.87 | 44.64 | 75.45 | 2537.56 | 2236.53 | 1812.68 | 1462.45 |
| Puerto Rico | 0.80 | 5.54 | 11.86 | 14.47 | 919.14 | 984.18 | 879.86 | 883.58 |
| Qatar | 2.63 | 18.39 | 39.30 | 41.92 | 707.55 | 756.95 | 600.33 | 553.40 |
| Republic of Korea | 1.34 | 9.35 | 20.02 | 24.11 | 515.29 | 516.49 | 518.90 | 477.23 |
| Republic of Moldova | 0.63 | 4.38 | 9.38 | 10.29 | 821.81 | 863.03 | 796.19 | 825.12 |
| Romania | 3.14 | 21.91 | 46.88 | 50.35 | 8219.70 | 8969.18 | 6812.75 | 7048.40 |
| Russian Federation | 1.33 | 9.28 | 19.91 | 22.00 | 119.72 | 136.71 | 93.51 | 97.02 |
| Rwanda | 7.28 | 51.13 | 109.31 | 196.46 | 14730.34 | 22919.29 | 10891.51 | 12025.49 |
| Saint Kitts and Nevis | 0.94 | 6.56 | 14.07 | 17.25 | 1812.43 | 1903.81 | 1662.44 | 1698.25 |
| Saint Lucia | 0.75 | 5.24 | 11.22 | 13.64 | 3065.92 | 3130.01 | 2962.14 | 3012.97 |
| Saint Vincent and the Grenadines | 1.39 | 9.68 | 20.72 | 25.89 | 4226.38 | 4465.06 | 3791.01 | 3878.31 |
| Samoa | 0.19 | 1.30 | 2.78 | 3.66 | 17968.18 | 19642.39 | 19061.84 | 17947.10 |
| San Marino | 2.35 | 16.39 | 35.05 | 54.84 | 557.20 | 494.94 | 397.70 | 311.59 |
| Sao Tome and Principe | 3.24 | 22.69 | 48.64 | 65.64 | 17175.38 | 17001.95 | 12075.67 | 12250.57 |
| Saudi Arabia | 1.24 | 8.65 | 18.48 | 19.86 | 415.08 | 432.95 | 311.57 | 214.87 |
| Senegal | 5.40 | 37.85 | 80.83 | 121.23 | 7433.75 | 9417.17 | 8248.23 | 8952.03 |
| Serbia | 1.07 | 7.48 | 16.06 | 17.72 | 15014.35 | 15359.24 | 12760.80 | 12584.16 |
| Seychelles | 0.68 | 4.74 | 10.16 | 22.42 | 2531.62 | 2579.49 | 2102.83 | 2034.15 |
| Sierra Leone | 4.49 | 31.69 | 67.85 | 97.67 | 27786.28 | 37728.87 | 21103.44 | 21663.67 |
| Singapore | 1.34 | 9.40 | 20.17 | 24.44 | 773.05 | 778.79 | 754.18 | 712.59 |
| Slovakia | 1.27 | 8.82 | 18.89 | 21.17 | 6324.27 | 6090.44 | 5128.52 | 5188.04 |
| Slovenia | 1.25 | 8.72 | 18.68 | 20.97 | 4343.07 | 4273.39 | 3631.17 | 3751.17 |
| Solomon Islands | 0.25 | 1.78 | 3.82 | 5.13 | 37339.44 | 44468.37 | 34238.20 | 32656.57 |
| Somalia | 25.12 | 176.95 | 374.06 | 1198.85 | 83077.71 | 86760.93 | 78117.94 | 79985.34 |
| South Africa | 2.98 | 20.89 | 44.78 | 56.75 | 9950.59 | 11128.18 | 7327.06 | 5953.02 |
| South Sudan | 5.24 | 37.07 | 79.11 | 124.34 | 33841.66 | 44883.07 | 27148.42 | 29128.72 |
| Spain | 2.39 | 16.69 | 35.71 | 55.90 | 840.55 | 818.55 | 736.80 | 698.58 |
| Sri Lanka | 1.05 | 7.35 | 15.75 | 51.79 | 5369.72 | 6252.01 | 4502.35 | 4100.89 |
| Sudan | 9.24 | 64.61 | 137.87 | 143.16 | 9129.90 | 15803.00 | 6685.09 | 6283.02 |
| Suriname | 1.26 | 8.79 | 18.81 | 23.32 | 4488.56 | 4858.33 | 4065.87 | 4221.95 |
| Sweden | 2.54 | 17.76 | 38.00 | 60.28 | 570.65 | 501.35 | 406.82 | 322.38 |
| Switzerland | 1.62 | 11.35 | 24.29 | 35.10 | 241.32 | 210.96 | 171.27 | 134.79 |
| Syrian Arab Republic | 8.57 | 59.86 | 127.97 | 133.58 | 6487.70 | 8107.16 | 3916.16 | 3722.09 |
| Taiwan (Province of China) | 0.30 | 2.10 | 4.51 | 6.75 | 681.69 | 647.90 | 602.95 | 596.73 |
| Tajikistan | 4.14 | 29.04 | 62.00 | 66.37 | 10805.76 | 14197.50 | 8639.46 | 8054.00 |
| Thailand | 0.80 | 5.56 | 11.90 | 29.51 | 1701.17 | 4314.59 | 1518.69 | 1491.54 |
| Timor-Leste | 0.73 | 5.17 | 11.06 | 25.78 | 20462.98 | 34094.66 | 11972.20 | 11481.35 |
| Togo | 6.14 | 43.00 | 91.80 | 146.38 | 21578.03 | 25167.98 | 18346.23 | 18866.49 |
| Tokelau | 0.23 | 1.60 | 3.44 | 4.56 | 10272.47 | 12047.32 | 10714.74 | 10037.32 |
| Tonga | 0.24 | 1.71 | 3.67 | 4.92 | 15211.52 | 16450.70 | 15699.19 | 14575.28 |
| Trinidad and Tobago | 1.76 | 12.24 | 26.22 | 33.29 | 1579.39 | 1662.97 | 1560.23 | 1562.36 |
| Tunisia | 2.63 | 18.30 | 39.15 | 41.81 | 2342.89 | 2301.18 | 1734.97 | 1707.77 |
| Türkiye | 7.48 | 52.12 | 111.30 | 116.26 | 3257.40 | 3981.14 | 2839.55 | 2783.98 |
| Turkmenistan | 2.17 | 15.23 | 32.51 | 35.21 | 2788.00 | 3114.48 | 2441.55 | 2386.26 |
| Tuvalu | 0.26 | 1.83 | 3.93 | 5.29 | 20624.20 | 23938.54 | 20075.16 | 19132.93 |
| Uganda | 3.56 | 25.01 | 53.45 | 75.57 | 11246.54 | 16781.23 | 7198.19 | 7335.13 |
| Ukraine | 4.55 | 31.67 | 67.57 | 70.67 | 828.34 | 933.89 | 606.02 | 648.62 |
| United Arab Emirates | 7.46 | 52.03 | 111.08 | 116.01 | 817.11 | 898.33 | 662.92 | 647.22 |
| United Kingdom | 2.46 | 17.23 | 36.89 | 57.67 | 831.55 | 739.53 | 604.38 | 467.63 |
| United Republic of Tanzania | 2.04 | 14.30 | 30.64 | 40.39 | 11142.13 | 16944.89 | 7256.62 | 7337.38 |
| United States of America | 1.28 | 8.93 | 19.13 | 23.31 | 687.89 | 714.69 | 580.56 | 452.97 |
| United States Virgin Islands | 0.71 | 4.92 | 10.51 | 12.87 | 977.67 | 1017.68 | 894.99 | 905.96 |
| Uruguay | 1.06 | 7.42 | 15.90 | 17.71 | 6917.85 | 7661.22 | 6122.86 | 5807.58 |
| Uzbekistan | 3.64 | 25.54 | 54.67 | 59.13 | 3038.01 | 4034.00 | 2563.44 | 2520.07 |
| Vanuatu | 0.93 | 6.53 | 13.98 | 33.33 | 36169.42 | 44009.83 | 33150.61 | 32086.37 |
| Venezuela (Bolivarian Republic of) | 3.17 | 22.17 | 47.43 | 53.67 | 7035.53 | 9515.66 | 8321.00 | 4963.59 |
| Viet Nam | 0.97 | 6.75 | 14.44 | 43.00 | 5370.36 | 8092.88 | 4003.78 | 2670.25 |
| Yemen | 19.00 | 132.88 | 282.03 | 284.66 | 18139.26 | 26609.51 | 10886.79 | 10367.36 |
| Zambia | 3.95 | 27.68 | 59.24 | 85.81 | 13518.10 | 21851.05 | 8873.91 | 9318.14 |
| Zimbabwe | 8.56 | 60.07 | 128.30 | 205.15 | 23699.03 | 29595.16 | 18430.25 | 18120.09 |

STable 3. Top three and bottom three regions of nutritional deficiency in 0-14 years.

| Measure | sex | Top three regions | | | Bottom three regions | | |
| --- | --- | --- | --- | --- | --- | --- | --- |
| 2021 ASR (per 100,000 people) | | | | | | | |
| Age-standardized DALY rate | | | | | | | |
|  | Iodine deficiency | North Africa and Middle East(34.89) | South Asia(59.36) | Central sub-Saharan Africa(61.86) | Tropical Latin America(0.60) | Oceania(0.73) | Andean Latin America(0.73) |
|  | Vitamin A deficiency | Central sub-Saharan Africa(114.63) | Western sub-Saharan Africa(183.15) | Eastern Sub-Saharan Africa(203.01) | Australasia(0.11) | High-income Asia Pacific(0.27) | High-income North America(0.52) |
|  | Dietary iron deficiency | Eastern Sub-Saharan Africa(1347.85) | Western sub-Saharan Africa(1688.03) | South Asia(2041.71) | High-income Asia Pacific(40.85) | Australasia(78.75) | High-income North America(83.34) |
| ASIR |  |  |  |  |  |  |  |
|  | Iodine deficiency | Eastern Sub-Saharan Africa(287.71) | South Asia(662.78) | Central sub-Saharan Africa(897.72) | Oceania(7.91) | Tropical Latin America(12.09) | Andean Latin America(13.08) |
|  | Vitamin A deficiency | Central sub-Saharan Africa(28403.76) | Western sub-Saharan Africa(32718.28) | Eastern Sub-Saharan Africa(40751.64) | Australasia(180.72) | Eastern Europe(705.21) | High-income Asia Pacific(728.87) |
| EAPC |  |  |  |  |  |  |  |
| DALY |  |  |  |  |  |  |  |
|  | Iodine deficiency | Tropical Latin America(0.33) | Australasia(1.13) | High-income North America(1.23) | Southeast Asia(-6.63) | Western Europe(-5.65) | Central Europe(-4.82) |
|  | Vitamin A deficiency | Central sub-Saharan Africa(-1.86) | Central Asia(-1.69) | Oceania(-1.33) | Eastern Europe(-6.96) | Central Europe(-6.73) | East Asia(-5.89) |
|  | Dietary iron deficiency | Western sub-Saharan Africa(0.34) | Oceania(0.52) | High-income North America(1.21) | East Asia(-4.69) | Eastern Europe(-4.17) | Central Europe(-4.11) |
| Incidence |  |  |  |  |  |  |  |
|  | Iodine deficiency | Tropical Latin America(0.67) | Australasia(1.13) | High-income North America(1.30) | Western Europe(-5.22) | Southeast Asia(-4.50) | Caribbean(-3.94) |
|  | Vitamin A deficiency | Central sub-Saharan Africa(-1.39) | Central Asia(-0.98) | Australasia(-0.67) | Eastern Europe(-6.41) | East Asia(-6.12) | Western Europe(-5.78) |

STable 4. Age-standardized DALYs rate of nutritional deficiency in 0-14 years at the national level and their changing trends from 1990 to 2021.

|  | 1990 | 2021 |  | 1990 | 2021 |  | 1990 | 2021 |  |
| --- | --- | --- | --- | --- | --- | --- | --- | --- | --- |
|  | Iodine deficiency | Iodine deficiency | Iodine deficiency | Vitamin A deficiency | Vitamin A deficiency | Vitamin A deficiency | Dietary iron deficiency | Dietary iron deficiency | Dietary iron deficiency |
| countries | 1990 No.(95%UI) | 2021 No.(95%UI) | 1990-2021 EAPC No.(95%CI) | 1990 No.(95%UI) | 2021 No.(95%UI) | 1990-2021 EAPC No.(95%CI) | 1990 No.(95%UI) | 2021 No.(95%UI) | 1990-2021 EAPC No.(95%CI) |
| Afghanistan | 29.52(26.97,32.28) | 23.91(22.90,24.95) | -1.10(-2.96,0.80) | 242.66(235.14,250.39) | 136.32(133.14,139.56) | -1.89(-2.45,-1.33) | 939.94(925.16,954.92) | 612.29(605.92,618.71) | -1.41(-1.74,-1.07) |
| Albania | 4.40(2.96,6.63) | 7.05(2.76,14.92) | 1.56(-0.13,3.27) | 227.31(212.15,243.34) | 45.90(34.71,59.97) | -5.14(-5.97,-4.29) | 820.09(791.67,849.37) | 436.98(399.67,477.14) | -1.58(-2.27,-0.88) |
| Algeria | 8.81(8.13,9.55) | 14.48(13.28,15.78) | -1.04(-2.43,0.38) | 82.92(79.90,86.03) | 38.83(36.73,41.03) | -2.74(-3.84,-1.63) | 1154.37(1143.12,1165.72) | 800.30(790.79,809.90) | -1.39(-2.39,-0.37) |
| American Samoa | 0.20(0.00,24.14) | 0.15(0.00,154.08) | -0.50(-3.06,2.13) | 31.76(11.01,72.74) | 29.18(0.50,202.97) | -1.12(-2.28,0.06) | 487.23(389.02,603.54) | 844.13(548.03,1255.31) | 0.54(-0.50,1.59) |
| Andorra | 1.86(0.00,203.01) | 4.84(0.00,278.15) | 0.01(-2.17,2.24) | 2.64(0.00,205.71) | 0.93(0.00,271.79) | -5.72(-7.37,-4.05) | 204.09(64.24,509.36) | 209.98(46.65,610.92) | -1.96(-3.54,-0.36) |
| Angola | 46.15(44.05,48.32) | 79.97(77.26,82.76) | -1.87(-2.87,-0.86) | 135.58(132.02,139.21) | 92.25(89.72,94.84) | -2.40(-3.53,-1.26) | 595.99(588.54,603.53) | 587.05(580.44,593.73) | -1.52(-2.41,-0.63) |
| Antigua and Barbuda | 3.28(0.00,89.64) | 5.30(0.00,122.06) | 1.08(-0.23,2.41) | 56.40(13.90,169.15) | 34.19(2.57,166.18) | -1.10(-1.90,-0.29) | 978.90(745.61,1271.03) | 803.66(561.05,1126.34) | -0.17(-0.76,0.43) |
| Argentina | 1.24(0.97,1.60) | 3.45(2.65,4.41) | 1.46(-0.51,3.46) | 64.13(61.29,67.08) | 15.86(14.40,17.45) | -3.87(-4.56,-3.18) | 209.53(204.78,214.37) | 168.18(162.93,173.58) | -0.84(-1.73,0.06) |
| Armenia | 6.70(5.07,8.73) | 19.17(12.20,28.81) | 1.68(0.18,3.21) | 2.44(1.51,3.79) | 1.69(0.31,5.87) | -1.48(-2.37,-0.58) | 860.34(840.89,880.15) | 982.72(934.89,1032.67) | -0.03(-1.01,0.96) |
| Australia | 0.83(0.57,1.19) | 2.19(1.44,3.24) | 2.60(0.85,4.38) | 0.03(0.00,0.19) | 0.01(0.00,0.44) | -2.91(-3.80,-2.02) | 55.99(53.48,58.59) | 67.66(62.97,72.63) | -0.03(-0.80,0.74) |
| Austria | 6.33(3.85,9.86) | 1.69(1.06,2.59) | -2.85(-4.19,-1.48) | 2.60(1.42,4.66) | 0.39(0.11,1.00) | -6.76(-7.85,-5.65) | 196.92(184.66,209.93) | 76.30(71.26,81.61) | -3.89(-5.09,-2.67) |
| Azerbaijan | 3.72(2.85,4.92) | 1.15(0.76,1.69) | -6.09(-8.94,-3.16) | 23.67(20.50,27.23) | 6.15(5.13,7.34) | -4.79(-6.40,-3.15) | 1673.66(1646.38,1701.32) | 651.19(640.10,662.43) | -3.20(-4.44,-1.95) |
| Bahamas | 2.37(0.11,11.86) | 6.30(0.08,41.17) | 0.96(-1.57,3.55) | 19.67(10.42,34.67) | 34.07(11.12,81.71) | 0.19(-1.48,1.90) | 559.28(502.96,620.77) | 1096.87(934.56,1280.68) | 0.52(-1.07,2.14) |
| Bahrain | 11.78(4.39,26.77) | 5.62(1.57,15.40) | -3.19(-3.94,-2.44) | 26.86(14.16,47.04) | 7.52(2.35,18.58) | -4.37(-4.66,-4.08) | 816.13(738.87,899.95) | 543.78(489.00,603.46) | -1.79(-2.04,-1.54) |
| Bangladesh | 49.23(48.53,49.95) | 36.01(34.81,37.24) | -2.17(-3.46,-0.86) | 102.90(101.97,103.83) | 25.21(24.35,26.10) | -3.68(-4.52,-2.84) | 946.48(943.60,949.38) | 1018.31(1012.43,1024.22) | 0.33(-0.38,1.05) |
| Barbados | 4.04(0.46,16.99) | 13.73(0.63,69.10) | -0.67(-2.53,1.23) | 24.03(11.52,45.07) | 28.81(6.36,88.79) | -0.82(-2.04,0.42) | 563.68(497.37,637.21) | 977.49(797.69,1188.88) | -0.37(-1.36,0.62) |
| Belarus | 2.12(1.49,2.92) | 6.03(3.56,9.62) | -1.16(-3.75,1.50) | 3.58(2.81,4.51) | 1.49(0.50,3.60) | -6.20(-7.80,-4.57) | 341.87(333.88,350.02) | 485.34(462.40,509.21) | -2.54(-4.23,-0.81) |
| Belgium | 2.32(1.52,3.63) | 3.82(2.24,6.19) | 2.13(0.72,3.56) | 3.86(2.44,5.88) | 1.08(0.31,2.77) | -3.40(-4.85,-1.93) | 274.28(260.90,288.22) | 219.03(204.95,233.87) | -0.23(-1.58,1.13) |
| Belize | 2.83(0.02,24.92) | 1.92(0.00,19.64) | -1.08(-2.29,0.16) | 113.79(74.05,168.77) | 60.95(34.89,99.91) | -1.99(-3.29,-0.66) | 1179.26(1044.55,1327.94) | 1145.46(1023.52,1278.93) | -0.12(-1.24,1.02) |
| Benin | 7.44(6.24,8.81) | 10.60(9.23,12.12) | 0.57(-0.37,1.52) | 268.37(261.34,275.56) | 264.35(257.23,271.64) | -0.43(-1.46,0.62) | 766.99(755.27,778.84) | 1106.35(1091.70,1121.17) | 0.57(-0.35,1.50) |
| Bermuda | 3.19(0.00,146.20) | 2.09(0.00,255.38) | 0.00(-1.49,1.51) | 14.04(0.01,165.53) | 3.95(0.00,259.21) | -4.15(-4.98,-3.31) | 654.79(413.53,999.53) | 465.01(210.68,916.52) | -0.86(-1.43,-0.28) |
| Bhutan | 12.06(5.87,22.74) | 1.96(0.33,10.59) | -4.16(-6.24,-2.03) | 342.32(304.51,384.05) | 80.20(58.41,108.08) | -5.88(-6.97,-4.78) | 3365.86(3234.03,3501.93) | 2601.50(2481.06,2727.10) | -1.26(-2.31,-0.20) |
| Bolivia (Plurinational State of) | 1.75(0.99,2.92) | 2.27(1.36,3.56) | -0.73(-2.47,1.04) | 91.90(85.65,98.53) | 45.50(41.78,49.52) | -2.64(-3.52,-1.76) | 2369.28(2335.40,2403.56) | 1025.09(1007.93,1042.53) | -2.44(-3.33,-1.53) |
| Bosnia and Herzegovina | 9.80(7.52,12.93) | 13.76(7.32,23.66) | -0.41(-1.84,1.04) | 124.83(113.15,137.51) | 32.56(23.08,45.02) | -3.27(-4.30,-2.23) | 859.78(828.25,892.32) | 503.10(462.56,546.51) | -0.42(-1.48,0.66) |
| Botswana | 2.45(1.20,5.28) | 1.69(0.67,4.28) | -1.33(-3.20,0.57) | 223.78(205.49,243.50) | 84.44(73.38,96.91) | -4.08(-5.28,-2.86) | 1078.91(1034.66,1124.72) | 1080.89(1038.98,1124.21) | -0.78(-1.79,0.24) |
| Brazil | 0.66(0.57,0.77) | 1.39(1.18,1.63) | -1.53(-3.33,0.30) | 152.39(150.52,154.28) | 51.07(49.93,52.23) | -4.46(-5.36,-3.56) | 1409.32(1403.59,1415.08) | 1001.93(996.71,1007.17) | -2.16(-3.01,-1.29) |
| Brunei Darussalam | 1.26(0.01,16.41) | 3.60(0.03,25.73) | 1.98(0.46,3.52) | 7.22(1.02,26.56) | 1.38(0.00,21.29) | -4.06(-5.11,-3.00) | 149.95(111.50,198.82) | 123.01(84.57,175.00) | 0.07(-0.95,1.09) |
| Bulgaria | 2.93(1.52,5.26) | 0.75(0.32,1.61) | -6.36(-8.00,-4.70) | 94.46(84.69,105.10) | 15.47(12.92,18.41) | -5.26(-6.24,-4.26) | 861.90(832.54,892.10) | 286.91(275.53,298.66) | -3.44(-4.41,-2.45) |
| Burkina Faso | 10.03(8.95,11.24) | 10.27(9.26,11.36) | -1.15(-2.37,0.09) | 699.38(687.92,711.01) | 305.71(299.88,311.63) | -2.27(-2.84,-1.70) | 1121.95(1108.12,1135.93) | 2610.92(2593.68,2628.26) | 3.59(2.96,4.22) |
| Burundi | 24.48(22.23,26.96) | 9.98(9.18,10.84) | -2.69(-3.69,-1.67) | 323.01(312.58,333.72) | 82.72(80.24,85.26) | -3.84(-4.81,-2.85) | 1283.57(1262.20,1305.25) | 774.90(767.31,782.54) | -0.68(-1.71,0.35) |
| Cabo Verde | 23.63(11.43,43.69) | 4.99(1.54,12.18) | -4.58(-6.54,-2.58) | 241.77(204.57,284.97) | 29.50(20.49,41.58) | -6.44(-7.78,-5.07) | 1498.90(1400.39,1603.44) | 728.91(679.83,780.91) | -1.94(-3.14,-0.72) |
| Cambodia | 21.76(19.38,24.37) | 6.07(4.74,7.65) | -4.72(-7.62,-1.74) | 294.01(286.82,301.37) | 81.32(77.24,85.59) | -5.65(-6.69,-4.59) | 1287.39(1271.20,1303.77) | 1100.53(1083.85,1117.43) | -1.52(-2.57,-0.46) |
| Cameroon | 4.48(3.86,5.17) | 8.29(7.42,9.25) | 2.43(1.52,3.36) | 261.61(256.63,266.67) | 182.42(178.08,186.83) | -2.46(-3.31,-1.62) | 504.75(498.10,511.48) | 1182.44(1171.64,1193.33) | 1.92(1.19,2.66) |
| Canada | 2.27(1.61,3.15) | 2.42(1.71,3.36) | 1.78(0.42,3.17) | 2.16(1.48,3.07) | 0.52(0.21,1.10) | -4.33(-5.83,-2.80) | 65.03(60.99,69.28) | 58.69(54.70,62.91) | -0.12(-1.48,1.25) |
| Central African Republic | 68.61(61.20,76.76) | 48.14(44.51,52.07) | -0.02(-1.14,1.12) | 461.45(441.96,481.66) | 364.85(352.02,378.07) | -0.40(-0.81,0.02) | 1025.19(995.56,1055.54) | 1086.01(1064.43,1107.97) | 0.48(-0.07,1.04) |
| Chad | 24.47(21.72,27.51) | 6.49(5.88,7.16) | -4.51(-5.44,-3.56) | 698.75(683.48,714.32) | 377.81(371.67,384.03) | -3.04(-3.58,-2.50) | 1675.97(1652.49,1699.73) | 1585.27(1572.99,1597.62) | -1.19(-1.66,-0.71) |
| Chile | 3.06(2.16,4.24) | 2.21(1.33,3.54) | -1.31(-3.24,0.65) | 23.71(20.90,26.81) | 5.67(4.06,7.74) | -4.89(-5.98,-3.79) | 281.69(271.95,291.72) | 196.90(186.88,207.34) | -1.49(-2.55,-0.41) |
| China | 6.23(6.12,6.33) | 2.27(2.19,2.36) | -2.79(-4.68,-0.86) | 30.96(30.76,31.17) | 17.34(16.99,17.70) | -3.20(-4.39,-1.98) | 316.36(315.70,317.01) | 238.00(236.69,239.32) | -2.01(-3.14,-0.87) |
| Colombia | 8.56(7.62,9.59) | 5.97(5.36,6.65) | 1.80(-0.21,3.85) | 38.92(36.86,41.08) | 9.26(8.25,10.38) | -3.68(-4.57,-2.78) | 600.36(592.25,608.57) | 325.36(319.19,331.63) | -0.81(-1.67,0.05) |
| Comoros | 6.83(2.29,16.74) | 10.03(3.60,22.48) | 0.29(-1.28,1.89) | 372.84(329.61,420.66) | 116.68(93.66,144.41) | -3.76(-4.51,-3.01) | 1367.84(1282.76,1457.51) | 1103.92(1031.98,1180.25) | -0.42(-1.16,0.33) |
| Congo | 88.60(78.94,99.22) | 55.96(51.60,60.74) | -1.45(-2.70,-0.19) | 469.10(445.92,493.26) | 322.80(308.86,337.28) | -1.67(-2.56,-0.76) | 1104.25(1069.32,1140.13) | 972.07(948.26,996.40) | -0.95(-1.72,-0.18) |
| Cook Islands | 0.70(0.00,237.03) | 0.16(0.00,468.28) | -1.70(-3.55,0.18) | 101.06(12.34,399.00) | 19.96(0.00,502.42) | -5.39(-5.99,-4.78) | 758.83(424.72,1273.34) | 445.69(132.60,1147.05) | -2.03(-2.50,-1.55) |
| Costa Rica | 11.32(7.74,16.05) | 2.97(1.82,4.59) | -1.91(-3.88,0.10) | 21.17(16.92,26.35) | 4.93(3.55,6.74) | -3.56(-4.47,-2.63) | 335.98(318.11,354.75) | 161.21(152.76,170.06) | -1.35(-2.15,-0.55) |
| Croatia | 5.39(2.61,9.90) | 1.35(0.45,3.14) | -4.91(-6.60,-3.19) | 47.03(38.88,56.58) | 7.33(5.09,10.31) | -5.61(-6.64,-4.58) | 659.93(627.59,693.66) | 195.05(182.50,208.32) | -3.52(-4.58,-2.44) |
| Cuba | 3.59(2.88,4.43) | 9.99(6.92,13.99) | 2.11(0.75,3.49) | 13.27(11.81,14.87) | 8.84(6.25,12.25) | -1.31(-2.43,-0.18) | 405.67(397.39,414.09) | 669.16(644.17,694.94) | 1.56(0.42,2.71) |
| Cyprus | 2.98(0.69,11.33) | 3.61(0.51,13.85) | 0.05(-1.61,1.74) | 7.54(2.52,18.60) | 1.16(0.00,10.30) | -5.21(-6.42,-3.99) | 263.15(224.61,306.96) | 168.50(136.17,206.74) | -0.64(-1.84,0.58) |
| Czechia | 1.31(0.73,2.41) | 1.94(0.79,4.00) | 1.84(0.69,3.00) | 43.02(38.18,48.39) | 11.53(8.65,15.15) | -3.51(-4.35,-2.66) | 629.34(610.01,649.19) | 367.17(349.67,385.39) | -1.11(-1.92,-0.29) |
| Côte d'Ivoire | 14.54(12.93,16.32) | 5.94(5.42,6.51) | -2.30(-3.65,-0.93) | 417.54(408.53,426.72) | 162.59(158.82,166.43) | -3.30(-3.58,-3.01) | 935.77(922.67,949.02) | 1284.05(1273.72,1294.46) | 1.10(0.81,1.39) |
| Democratic People's Republic of Korea | 0.53(0.35,0.79) | 1.38(0.75,2.33) | -0.47(-1.84,0.92) | 53.20(51.30,55.16) | 25.28(22.71,28.10) | -1.93(-2.76,-1.10) | 382.06(376.91,387.28) | 366.12(355.83,376.66) | 0.53(-0.20,1.27) |
| Democratic Republic of the Congo | 173.24(169.93,176.59) | 64.50(63.68,65.32) | -1.60(-2.84,-0.35) | 288.76(285.04,292.52) | 128.98(127.77,130.21) | -2.32(-3.36,-1.28) | 1115.98(1108.29,1123.72) | 473.92(471.62,476.22) | -2.36(-2.93,-1.79) |
| Denmark | 2.50(1.38,4.19) | 5.83(3.05,10.14) | 3.55(2.00,5.12) | 1.30(0.64,2.48) | 0.33(0.01,2.32) | -4.38(-5.75,-3.00) | 123.76(116.09,131.86) | 116.77(105.34,129.31) | -0.63(-1.87,0.63) |
| Djibouti | 32.49(22.59,46.74) | 19.79(15.68,24.73) | -1.40(-2.96,0.19) | 257.08(221.13,297.80) | 57.75(50.20,66.16) | -4.40(-5.05,-3.75) | 1217.85(1137.07,1303.37) | 658.97(632.99,685.79) | -1.49(-2.17,-0.81) |
| Dominica | 4.55(0.08,31.97) | 4.21(0.00,165.02) | 0.50(-1.14,2.17) | 29.45(10.37,68.47) | 28.10(0.70,202.94) | -0.14(-1.63,1.38) | 672.89(564.07,798.56) | 1153.27(817.29,1599.91) | 1.36(0.07,2.67) |
| Dominican Republic | 5.74(4.68,7.07) | 3.27(2.48,4.35) | -0.66(-2.11,0.81) | 91.73(86.06,97.71) | 35.47(32.01,39.25) | -2.58(-3.81,-1.33) | 1172.36(1151.74,1193.30) | 897.88(879.67,916.41) | -0.32(-1.43,0.81) |
| Ecuador | 1.58(0.95,2.52) | 1.08(0.56,1.90) | -1.92(-4.00,0.21) | 46.73(42.71,51.04) | 15.41(13.46,17.59) | -3.00(-4.08,-1.90) | 216.73(208.42,225.32) | 79.35(75.30,83.61) | -2.54(-3.17,-1.91) |
| Egypt | 49.47(47.76,51.23) | 4.54(4.29,4.80) | -6.34(-7.97,-4.68) | 49.45(48.02,50.93) | 11.64(11.27,12.03) | -3.44(-4.48,-2.39) | 848.58(842.45,854.75) | 319.87(317.92,321.83) | -2.39(-3.24,-1.53) |
| El Salvador | 6.26(5.09,7.77) | 4.77(3.61,6.41) | -0.09(-1.11,0.95) | 65.40(59.97,71.24) | 17.16(13.93,20.99) | -4.14(-4.99,-3.29) | 419.47(405.91,433.41) | 268.00(255.17,281.39) | -1.02(-1.88,-0.16) |
| Equatorial Guinea | 222.84(188.99,261.25) | 47.63(36.95,60.71) | -3.94(-5.36,-2.50) | 438.46(396.24,484.59) | 99.32(83.21,117.86) | -5.88(-6.55,-5.21) | 1104.63(1035.90,1177.28) | 855.26(808.61,904.15) | -1.80(-2.48,-1.12) |
| Eritrea | 6.34(4.89,8.22) | 4.25(3.30,5.50) | 1.56(0.01,3.14) | 477.21(460.77,494.13) | 197.54(188.34,207.10) | -2.13(-2.81,-1.45) | 1534.83(1504.98,1565.18) | 1495.73(1470.76,1521.07) | 0.73(0.06,1.41) |
| Estonia | 1.61(0.36,6.57) | 1.32(0.11,9.40) | -1.90(-3.05,-0.74) | 4.96(1.66,12.00) | 0.59(0.00,8.79) | -7.47(-8.58,-6.35) | 585.15(540.76,632.59) | 316.84(275.80,363.03) | -3.11(-4.16,-2.05) |
| Eswatini | 15.83(9.27,25.40) | 3.45(1.56,7.87) | -4.57(-5.58,-3.56) | 158.94(140.56,179.57) | 84.00(69.64,100.78) | -2.75(-3.75,-1.74) | 663.72(622.92,706.87) | 876.37(829.13,925.92) | 0.23(-0.59,1.05) |
| Ethiopia | 50.59(49.53,51.68) | 39.68(38.63,40.75) | -0.01(-1.58,1.59) | 400.12(396.36,403.91) | 163.86(161.60,166.14) | -4.03(-5.05,-3.01) | 1469.68(1462.39,1477.00) | 1424.27(1417.62,1430.93) | -0.73(-1.53,0.08) |
| Fiji | 0.66(0.05,3.22) | 0.35(0.00,7.49) | -1.41(-3.06,0.28) | 66.51(56.05,78.50) | 76.22(56.28,101.34) | -1.37(-2.24,-0.49) | 568.35(537.67,600.47) | 949.24(879.17,1023.92) | -0.23(-0.93,0.48) |
| Finland | 1.97(1.07,3.92) | 7.93(4.20,13.67) | 2.19(0.31,4.11) | 3.03(1.41,5.86) | 0.54(0.04,3.20) | -6.33(-7.58,-5.08) | 241.05(223.88,259.30) | 131.43(117.28,147.08) | -3.08(-4.38,-1.77) |
| France | 1.96(1.66,2.32) | 2.91(2.50,3.40) | 2.58(1.05,4.13) | 0.73(0.48,1.07) | 0.24(0.10,0.49) | -2.75(-3.74,-1.75) | 281.15(275.62,286.76) | 168.51(164.04,173.08) | -1.25(-2.14,-0.34) |
| Gabon | 25.05(20.31,30.62) | 17.51(13.88,21.84) | -4.15(-5.93,-2.33) | 129.09(117.62,141.40) | 53.55(47.55,60.16) | -3.57(-4.70,-2.42) | 1048.99(1016.11,1082.70) | 799.66(775.34,824.60) | -2.46(-3.71,-1.19) |
| Gambia | 13.39(10.13,17.42) | 12.18(9.91,14.88) | -0.96(-1.89,-0.02) | 346.18(328.23,364.88) | 159.38(150.66,168.51) | -2.89(-3.68,-2.09) | 1526.24(1488.18,1565.05) | 1463.55(1436.70,1490.81) | -0.83(-1.74,0.09) |
| Georgia | 3.90(2.09,6.79) | 1.44(0.71,2.69) | -1.48(-3.63,0.71) | 18.63(14.09,24.25) | 4.83(3.30,6.87) | -3.71(-5.19,-2.21) | 1441.58(1400.06,1484.10) | 536.79(519.06,555.01) | -3.06(-4.14,-1.97) |
| Germany | 6.18(5.32,7.14) | 4.22(3.55,5.00) | -0.82(-3.00,1.41) | 0.79(0.55,1.11) | 0.38(0.18,0.70) | -3.17(-4.25,-2.09) | 173.98(170.24,177.80) | 169.22(164.45,174.11) | -0.78(-1.83,0.29) |
| Ghana | 34.43(31.94,37.07) | 8.53(7.93,9.18) | -2.45(-3.75,-1.14) | 358.51(351.84,365.31) | 220.26(215.94,224.64) | -2.25(-3.20,-1.30) | 916.29(905.52,927.18) | 1340.88(1330.39,1351.44) | 0.54(-0.41,1.50) |
| Greece | 2.79(1.95,4.22) | 2.46(1.51,4.19) | 2.04(0.35,3.76) | 5.63(3.73,8.24) | 1.79(0.74,3.82) | -4.01(-5.25,-2.74) | 259.19(244.73,274.32) | 203.38(189.41,218.19) | -1.01(-2.22,0.20) |
| Greenland | 1.10(0.00,88.66) | 0.82(0.00,42.79) | -1.48(-3.68,0.78) | 1.69(0.00,90.11) | 0.35(0.00,42.03) | -4.64(-5.61,-3.66) | 120.70(49.08,260.11) | 42.20(12.69,106.55) | -2.28(-3.14,-1.41) |
| Grenada | 4.41(0.12,47.80) | 2.60(0.00,32.89) | -3.94(-5.32,-2.55) | 85.63(41.94,161.41) | 19.62(4.63,58.07) | -4.79(-6.13,-3.44) | 1125.29(941.20,1338.53) | 537.54(433.59,661.10) | -2.31(-3.37,-1.24) |
| Guam | 0.20(0.00,11.58) | 0.14(0.00,13.77) | -0.15(-1.82,1.54) | 22.55(10.01,44.31) | 7.61(1.22,26.02) | -3.13(-4.08,-2.17) | 321.04(266.48,383.96) | 280.18(225.20,345.19) | -0.20(-1.00,0.62) |
| Guatemala | 5.90(5.00,6.97) | 16.62(14.20,19.35) | 2.39(1.37,3.42) | 77.60(73.19,82.22) | 22.16(19.74,24.84) | -3.89(-4.38,-3.41) | 780.65(767.34,794.16) | 685.38(670.79,700.24) | -0.52(-0.83,-0.21) |
| Guinea | 42.05(38.19,46.23) | 9.32(8.55,10.14) | -4.48(-5.16,-3.79) | 492.13(478.80,505.77) | 152.54(149.22,155.91) | -2.47(-3.43,-1.49) | 833.09(816.20,850.28) | 939.26(931.07,947.50) | 1.71(0.75,2.68) |
| Guinea-Bissau | 18.91(15.04,23.50) | 24.83(19.39,31.51) | -0.52(-2.09,1.08) | 343.78(326.63,361.63) | 316.09(294.69,338.74) | -1.95(-2.90,-0.98) | 1135.66(1104.35,1167.66) | 2339.70(2281.34,2399.29) | 0.81(-0.20,1.83) |
| Guyana | 15.77(8.58,26.73) | 4.16(1.59,8.93) | -2.73(-4.28,-1.16) | 98.04(80.91,118.21) | 23.17(16.70,31.53) | -3.85(-4.88,-2.81) | 939.37(882.75,999.04) | 494.25(462.20,528.08) | -0.84(-1.87,0.20) |
| Haiti | 8.62(7.42,9.96) | 11.61(9.86,13.62) | -1.54(-3.08,0.03) | 175.75(170.27,181.37) | 220.71(212.29,229.40) | -1.89(-3.01,-0.77) | 1295.17(1280.30,1310.19) | 2574.95(2545.87,2604.30) | -0.27(-1.37,0.85) |
| Honduras | 12.33(9.85,15.31) | 5.50(4.73,6.38) | -3.01(-4.25,-1.75) | 66.94(60.84,73.53) | 11.76(10.54,13.09) | -3.96(-5.03,-2.89) | 794.40(773.13,816.16) | 325.62(319.12,332.22) | -1.67(-2.63,-0.71) |
| Hungary | 2.89(2.03,3.99) | 3.49(1.72,6.36) | 2.19(1.02,3.38) | 33.61(30.85,36.57) | 14.43(10.94,18.83) | -2.19(-2.94,-1.44) | 422.27(412.15,432.60) | 378.44(358.84,398.91) | 0.53(-0.24,1.30) |
| Iceland | 2.89(0.00,30.93) | 1.25(0.00,11.37) | -0.77(-2.18,0.67) | 1.61(0.00,27.67) | 0.24(0.00,9.41) | -7.24(-8.17,-6.29) | 138.22(95.49,197.68) | 62.96(44.40,87.75) | -3.83(-4.91,-2.74) |
| India | 271.71(270.60,272.82) | 32.99(32.61,33.37) | -6.59(-8.06,-5.10) | 318.61(317.60,319.63) | 170.83(169.94,171.73) | -3.18(-4.25,-2.11) | 2417.05(2414.15,2419.95) | 2797.92(2794.19,2801.66) | -1.34(-2.31,-0.37) |
| Indonesia | 6.45(6.20,6.71) | 2.40(2.15,2.66) | -3.03(-4.93,-1.10) | 256.49(254.29,258.71) | 39.85(38.96,40.75) | -4.92(-5.88,-3.95) | 668.11(664.78,671.45) | 441.99(439.16,444.84) | -0.11(-0.86,0.64) |
| Iran (Islamic Republic of) | 7.53(7.08,8.00) | 3.22(2.90,3.59) | -1.35(-3.10,0.43) | 69.69(67.71,71.71) | 7.61(6.85,8.45) | -6.41(-7.61,-5.20) | 1155.94(1148.03,1163.89) | 669.04(661.76,676.38) | -1.43(-2.49,-0.36) |
| Iraq | 25.69(23.79,27.72) | 25.93(24.20,27.76) | -1.93(-3.70,-0.14) | 97.74(93.98,101.62) | 42.70(40.42,45.10) | -3.29(-4.05,-2.52) | 1180.74(1167.57,1194.03) | 873.10(862.76,883.54) | -1.55(-2.24,-0.85) |
| Ireland | 2.33(1.37,3.79) | 7.58(4.19,12.66) | 4.28(2.68,5.90) | 2.50(1.44,4.08) | 0.35(0.01,2.55) | -6.49(-7.55,-5.41) | 159.92(150.55,169.78) | 112.82(100.89,126.01) | -1.82(-2.88,-0.74) |
| Israel | 6.61(4.20,9.94) | 1.67(1.22,2.26) | -4.06(-5.15,-2.95) | 21.83(18.04,26.33) | 3.96(3.19,4.87) | -5.38(-6.42,-4.34) | 215.65(203.44,228.53) | 101.42(97.31,105.65) | -2.50(-3.55,-1.44) |
| Italy | 12.41(11.44,13.48) | 22.49(20.02,25.17) | 0.66(-1.73,3.10) | 5.30(4.47,6.27) | 1.17(0.75,1.77) | -4.95(-6.51,-3.35) | 211.43(205.78,217.20) | 113.94(109.37,118.69) | -2.02(-3.40,-0.63) |
| Jamaica | 5.46(3.73,7.73) | 10.97(5.68,19.23) | 1.59(-0.37,3.58) | 35.50(31.25,40.21) | 40.62(30.58,53.20) | 1.09(0.18,2.01) | 541.56(524.43,559.16) | 977.50(925.13,1032.29) | 2.05(1.22,2.88) |
| Japan | 5.76(5.06,6.53) | 1.40(1.16,1.70) | -0.31(-2.41,1.83) | 1.67(1.35,2.06) | 0.54(0.34,0.83) | -3.12(-3.82,-2.41) | 106.59(103.84,109.40) | 68.97(66.53,71.49) | -0.84(-1.55,-0.12) |
| Jordan | 7.62(6.33,9.13) | 14.05(11.61,16.89) | 4.28(3.28,5.28) | 52.23(48.59,56.08) | 51.30(46.25,56.77) | -0.92(-1.48,-0.36) | 656.32(643.26,669.59) | 803.57(783.77,823.77) | 0.61(-0.09,1.32) |
| Kazakhstan | 12.67(10.90,14.68) | 5.44(4.31,6.80) | -4.85(-6.32,-3.35) | 260.53(251.73,269.56) | 51.12(48.17,54.24) | -4.06(-5.42,-2.68) | 1991.93(1968.65,2015.44) | 851.11(838.01,864.39) | -2.77(-3.87,-1.65) |
| Kenya | 4.41(3.94,4.94) | 12.64(11.57,13.80) | 2.54(1.07,4.04) | 303.23(298.08,308.45) | 130.62(127.70,133.59) | -1.80(-2.74,-0.85) | 649.07(641.42,656.80) | 469.63(464.04,475.29) | -0.54(-1.38,0.31) |
| Kiribati | 1.96(0.00,48.83) | 0.64(0.00,14.79) | -4.01(-5.11,-2.88) | 206.08(137.57,304.40) | 97.00(68.02,135.57) | -2.61(-3.69,-1.50) | 985.88(824.85,1175.04) | 676.75(595.64,766.94) | -1.46(-2.50,-0.40) |
| Kuwait | 5.92(2.64,11.90) | 7.59(4.03,13.08) | 1.58(-0.24,3.43) | 37.38(27.60,49.74) | 23.33(17.24,31.07) | 0.49(-0.59,1.58) | 801.38(754.42,850.71) | 471.07(443.08,500.56) | -0.02(-1.10,1.08) |
| Kyrgyzstan | 3.00(1.70,5.02) | 1.60(1.00,2.61) | -1.22(-2.27,-0.16) | 62.39(55.54,69.92) | 38.27(34.06,42.91) | -0.56(-1.83,0.73) | 1717.82(1681.45,1754.83) | 1041.34(1018.86,1064.25) | -1.15(-2.26,-0.03) |
| Lao People's Democratic Republic | 5.50(4.26,7.12) | 1.04(0.44,2.21) | -6.62(-8.72,-4.47) | 316.57(303.97,329.60) | 89.73(82.72,97.23) | -4.35(-5.31,-3.38) | 944.28(923.02,965.95) | 729.90(709.92,750.35) | -1.04(-1.90,-0.18) |
| Latvia | 1.39(0.43,4.44) | 3.35(0.39,12.60) | -1.44(-3.83,1.00) | 4.89(2.18,9.76) | 0.92(0.00,8.36) | -6.75(-7.88,-5.60) | 589.15(554.59,625.50) | 447.07(398.89,499.93) | -2.82(-3.98,-1.63) |
| Lebanon | 43.60(36.38,51.89) | 6.02(4.76,7.55) | -5.85(-7.58,-4.09) | 26.51(21.84,32.08) | 3.48(2.47,4.80) | -6.90(-7.34,-6.46) | 861.09(832.53,890.52) | 333.72(322.97,344.75) | -3.41(-4.02,-2.79) |
| Lesotho | 92.94(83.72,103.24) | 18.65(14.37,24.36) | -0.45(-2.74,1.89) | 300.66(279.06,323.64) | 134.24(118.38,151.82) | -1.95(-3.06,-0.82) | 915.54(877.94,954.47) | 1146.16(1099.74,1194.22) | 1.88(0.86,2.90) |
| Liberia | 4.76(3.48,6.35) | 5.46(4.30,6.99) | -2.56(-4.01,-1.08) | 232.39(223.14,241.94) | 172.50(163.21,182.23) | -3.63(-4.65,-2.61) | 861.26(843.39,879.42) | 1459.14(1433.02,1485.67) | -0.66(-1.62,0.30) |
| Libya | 18.28(14.75,22.49) | 17.71(15.09,20.67) | 0.81(-0.56,2.21) | 61.13(54.32,68.61) | 16.72(14.40,19.34) | -2.35(-3.36,-1.34) | 878.10(852.02,904.84) | 373.07(362.01,384.42) | -1.38(-2.33,-0.43) |
| Lithuania | 2.17(0.69,5.45) | 2.24(0.32,8.63) | -2.08(-3.88,-0.25) | 5.28(2.68,9.58) | 0.80(0.01,6.52) | -6.77(-7.47,-6.06) | 636.11(602.99,670.72) | 473.07(429.39,520.32) | -1.90(-2.67,-1.13) |
| Luxembourg | 2.15(0.04,23.47) | 3.49(0.07,24.26) | -0.64(-1.97,0.71) | 2.77(0.01,25.47) | 0.58(0.00,19.70) | -7.37(-8.37,-6.36) | 246.96(186.81,322.02) | 155.61(109.93,215.16) | -3.87(-4.88,-2.85) |
| Madagascar | 15.64(13.81,17.65) | 10.10(9.48,10.76) | 0.00(-1.62,1.64) | 230.01(224.13,236.02) | 78.23(76.47,80.04) | -2.99(-3.92,-2.05) | 1003.62(991.24,1016.13) | 602.73(597.80,607.69) | -1.19(-1.90,-0.48) |
| Malawi | 12.86(11.66,14.18) | 11.02(10.18,11.96) | 0.41(-0.43,1.27) | 463.88(454.83,473.07) | 190.15(185.09,195.33) | -1.12(-1.96,-0.29) | 2131.36(2112.07,2150.78) | 1371.86(1358.41,1385.44) | 0.24(-0.71,1.19) |
| Malaysia | 11.76(10.94,12.63) | 2.68(2.25,3.17) | -5.27(-7.48,-3.00) | 14.78(13.79,15.82) | 1.40(1.13,1.72) | -5.87(-6.98,-4.74) | 562.72(556.63,568.86) | 311.72(307.43,316.06) | -0.84(-1.91,0.25) |
| Maldives | 5.05(0.37,22.41) | 0.42(0.00,5.90) | -8.62(-9.94,-7.29) | 317.68(270.10,373.30) | 18.54(10.60,30.51) | -8.77(-9.58,-7.95) | 1538.42(1426.86,1658.04) | 484.32(439.30,533.00) | -3.74(-4.43,-3.04) |
| Mali | 12.80(11.13,14.68) | 9.19(8.28,10.18) | -3.01(-4.17,-1.83) | 707.38(694.31,720.65) | 423.94(417.46,430.50) | -2.05(-2.91,-1.18) | 1811.58(1790.63,1832.72) | 3712.04(3692.36,3731.80) | 1.99(0.90,3.10) |
| Malta | 2.19(0.12,20.92) | 2.00(0.06,12.61) | -4.18(-5.91,-2.42) | 7.01(0.79,29.54) | 0.80(0.00,10.72) | -5.79(-6.77,-4.80) | 307.97(243.44,385.72) | 95.42(70.25,127.36) | -2.65(-3.67,-1.62) |
| Marshall Islands | 0.79(0.00,28.80) | 0.62(0.00,91.42) | -2.33(-4.34,-0.29) | 189.79(130.06,269.72) | 175.87(80.16,343.41) | -0.67(-1.66,0.34) | 624.16(513.99,753.25) | 1077.75(815.30,1405.06) | 0.93(0.01,1.85) |
| Mauritania | 20.66(17.18,24.87) | 31.90(26.99,37.46) | -0.95(-1.92,0.02) | 391.50(371.80,412.05) | 102.60(94.98,110.76) | -2.87(-3.95,-1.78) | 2146.58(2101.53,2192.44) | 1583.86(1552.16,1616.11) | -0.05(-0.97,0.89) |
| Mauritius | 3.82(1.83,7.31) | 0.95(0.03,9.24) | -7.54(-9.37,-5.68) | 63.39(53.69,74.49) | 21.25(11.06,37.72) | -3.04(-3.66,-2.41) | 517.13(489.24,546.34) | 597.54(535.88,664.86) | 0.49(0.11,0.87) |
| Mexico | 13.77(13.00,14.58) | 5.63(5.31,5.96) | -1.17(-2.69,0.38) | 53.87(52.65,55.11) | 12.29(11.88,12.71) | -3.10(-4.48,-1.71) | 401.42(397.99,404.87) | 163.22(161.72,164.73) | -1.16(-2.52,0.22) |
| Micronesia (Federated States of) | 0.81(0.00,34.11) | 0.53(0.00,56.10) | -3.13(-5.13,-1.09) | 399.31(301.87,520.72) | 236.65(144.74,370.15) | -3.13(-4.34,-1.92) | 1053.07(889.12,1240.63) | 995.70(794.79,1236.04) | -1.70(-2.80,-0.59) |
| Monaco | 2.51(0.00,387.86) | 1.59(0.00,106.91) | -3.89(-5.14,-2.64) | 0.74(0.00,385.98) | 0.12(0.00,104.57) | -6.06(-6.96,-5.15) | 97.00(4.45,542.11) | 40.02(3.71,170.90) | -3.74(-4.53,-2.95) |
| Mongolia | 7.79(6.04,9.92) | 3.73(2.54,5.30) | -4.99(-7.22,-2.70) | 30.67(26.92,34.80) | 11.01(9.03,13.33) | -3.43(-4.53,-2.31) | 1310.25(1285.42,1335.46) | 788.38(770.94,806.14) | -2.10(-3.50,-0.69) |
| Montenegro | 1.96(0.19,12.72) | 3.61(0.06,23.06) | -1.19(-2.55,0.19) | 42.67(26.44,66.40) | 17.61(6.10,42.24) | -4.33(-5.22,-3.44) | 549.73(485.68,620.72) | 433.97(362.49,516.80) | -1.70(-2.27,-1.12) |
| Morocco | 29.08(28.00,30.20) | 52.71(49.74,55.81) | 1.36(0.29,2.45) | 86.61(84.66,88.60) | 50.23(47.69,52.88) | -2.18(-3.20,-1.15) | 721.10(715.49,726.75) | 796.44(786.36,806.64) | -0.13(-1.04,0.78) |
| Mozambique | 32.98(30.64,35.46) | 11.47(10.48,12.54) | -3.01(-4.81,-1.18) | 536.36(526.64,546.22) | 257.81(252.87,262.82) | -1.98(-2.71,-1.25) | 1452.75(1437.62,1468.01) | 1576.28(1564.61,1588.03) | 0.21(-0.36,0.79) |
| Myanmar | 8.06(7.50,8.66) | 3.19(2.70,3.75) | -9.97(-12.27,-7.62) | 396.61(391.13,402.14) | 114.92(111.47,118.47) | -7.08(-7.97,-6.18) | 1699.27(1688.12,1710.49) | 1707.62(1694.46,1720.86) | -3.20(-4.09,-2.30) |
| Namibia | 4.86(3.23,7.08) | 10.98(6.85,16.75) | -0.93(-2.24,0.40) | 112.38(103.54,121.80) | 64.64(55.42,75.19) | -3.27(-4.23,-2.30) | 812.41(788.46,836.92) | 946.77(910.17,984.66) | -0.52(-1.31,0.27) |
| Nauru | 0.87(0.00,361.28) | 0.22(0.00,358.65) | -5.92(-6.76,-5.08) | 139.81(14.21,587.96) | 82.47(2.87,493.59) | -2.37(-3.67,-1.05) | 939.30(499.40,1650.18) | 928.87(482.57,1651.58) | -1.26(-2.22,-0.29) |
| Nepal | 10.32(9.54,11.18) | 3.46(3.05,3.91) | -1.28(-3.13,0.61) | 235.41(230.27,240.65) | 33.01(31.68,34.39) | -5.77(-6.67,-4.87) | 1229.22(1217.88,1240.66) | 743.96(737.66,750.30) | -0.75(-1.62,0.13) |
| Netherlands | 2.24(1.59,3.20) | 3.49(2.26,5.21) | 2.44(0.69,4.22) | 2.26(1.39,3.52) | 0.53(0.12,1.55) | -4.74(-5.98,-3.48) | 254.49(244.16,265.18) | 167.77(157.76,178.29) | -1.59(-2.73,-0.44) |
| New Zealand | 1.21(0.46,3.24) | 3.50(1.44,7.19) | 1.86(0.13,3.62) | 1.24(0.32,3.58) | 0.32(0.01,2.38) | -2.75(-4.07,-1.42) | 124.00(112.21,136.87) | 84.01(74.08,95.13) | -0.93(-1.81,-0.04) |
| Nicaragua | 5.77(4.69,7.04) | 12.36(9.45,15.96) | -0.57(-2.90,1.81) | 27.19(24.69,29.89) | 10.66(7.85,14.20) | -5.48(-6.86,-4.07) | 358.24(349.16,367.50) | 502.04(481.89,522.87) | -0.98(-2.17,0.22) |
| Niger | 43.97(40.69,47.47) | 14.35(13.54,15.20) | -3.93(-5.17,-2.67) | 592.93(582.34,603.70) | 509.76(503.69,515.88) | -0.62(-1.47,0.23) | 1178.13(1163.51,1192.92) | 1398.68(1388.75,1408.68) | 0.29(-0.65,1.24) |
| Nigeria | 11.14(10.58,11.72) | 9.00(8.65,9.36) | -2.64(-4.15,-1.11) | 151.61(149.89,153.35) | 69.54(68.69,70.40) | -3.24(-3.85,-2.62) | 1536.76(1531.00,1542.54) | 1840.30(1835.83,1844.78) | 0.41(-0.22,1.05) |
| Niue | 2.04(0.00,2512.55) | 0.45(0.00,5475.32) | -6.57(-8.17,-4.95) | 87.58(0.00,2639.01) | 48.66(0.00,5562.72) | -2.09(-3.32,-0.85) | 811.85(85.03,3723.10) | 737.50(3.32,6707.27) | -0.49(-1.69,0.72) |
| North Macedonia | 3.02(0.82,8.30) | 1.12(0.22,3.71) | -6.10(-7.92,-4.25) | 158.27(136.23,183.03) | 22.60(16.91,29.73) | -6.03(-7.11,-4.95) | 938.20(885.12,993.86) | 291.86(270.83,314.23) | -3.37(-4.36,-2.38) |
| Northern Mariana Islands | 0.39(0.00,108.64) | 0.76(0.00,197.87) | 0.76(-1.13,2.69) | 27.85(1.42,152.71) | 19.59(0.03,227.23) | 0.28(-0.67,1.23) | 467.56(295.74,716.27) | 574.50(318.33,977.30) | 1.12(0.40,1.84) |
| Norway | 6.46(3.45,11.10) | 1.75(1.02,2.90) | -1.70(-3.59,0.23) | 1.78(0.66,4.40) | 0.16(0.01,0.86) | -6.87(-7.92,-5.81) | 176.37(161.83,192.11) | 65.69(60.06,71.74) | -2.50(-3.47,-1.52) |
| Oman | 13.89(9.67,19.41) | 5.66(4.04,8.04) | -2.21(-3.53,-0.87) | 95.40(85.60,106.20) | 35.13(29.60,41.51) | -3.18(-4.36,-1.99) | 585.41(561.58,610.20) | 498.51(475.94,521.96) | -1.39(-2.36,-0.41) |
| Pakistan | 25.13(24.62,25.65) | 57.05(56.02,58.09) | -0.93(-2.93,1.10) | 143.59(142.50,144.68) | 49.97(49.12,50.84) | -4.70(-5.73,-3.65) | 1740.64(1736.67,1744.63) | 2639.72(2633.53,2645.93) | 0.03(-1.06,1.13) |
| Palau | 1.83(0.00,389.69) | 0.28(0.00,570.38) | -5.79(-7.43,-4.11) | 55.43(0.75,469.14) | 35.17(0.00,629.45) | -2.04(-3.36,-0.69) | 769.51(383.64,1439.95) | 777.39(298.45,1713.92) | -0.81(-1.98,0.37) |
| Palestine | 18.04(13.58,23.56) | 4.73(2.97,7.26) | -5.08(-6.94,-3.18) | 96.55(87.41,106.55) | 48.19(41.84,55.29) | -3.82(-4.90,-2.73) | 604.17(580.76,628.42) | 574.35(552.30,597.11) | -1.69(-2.65,-0.71) |
| Panama | 4.44(2.81,7.17) | 4.89(2.50,8.65) | -0.48(-1.03,0.08) | 26.14(20.58,32.91) | 10.38(7.20,14.70) | -3.12(-3.66,-2.58) | 542.12(515.11,570.31) | 304.70(286.07,324.37) | -1.84(-2.19,-1.49) |
| Papua New Guinea | 0.94(0.48,1.65) | 0.42(0.22,0.84) | -4.21(-5.31,-3.10) | 70.80(66.71,75.09) | 77.22(72.77,81.89) | -0.06(-0.87,0.76) | 451.56(441.27,462.06) | 835.58(820.93,850.45) | 1.63(0.78,2.49) |
| Paraguay | 1.35(0.80,2.16) | 1.20(0.46,2.70) | -1.70(-2.57,-0.82) | 56.54(52.60,60.72) | 48.56(42.43,55.39) | -0.83(-1.92,0.26) | 471.52(460.30,482.97) | 640.73(618.63,663.48) | 0.71(-0.25,1.67) |
| Peru | 1.35(0.94,1.91) | 0.29(0.18,0.51) | -6.51(-8.39,-4.59) | 135.59(130.74,140.58) | 29.73(27.95,31.61) | -5.90(-6.91,-4.87) | 1275.04(1260.45,1289.77) | 436.25(429.25,443.35) | -4.22(-5.03,-3.42) |
| Philippines | 11.00(10.52,11.50) | 5.51(5.21,5.82) | -4.23(-5.48,-2.96) | 108.81(106.63,111.03) | 23.55(23.01,24.10) | -3.79(-4.67,-2.90) | 905.43(899.23,911.67) | 421.75(419.39,424.12) | -1.67(-2.38,-0.96) |
| Poland | 1.63(1.36,1.96) | 1.25(0.93,1.64) | 0.95(-0.05,1.96) | 51.94(50.23,53.69) | 8.73(7.92,9.60) | -3.70(-4.73,-2.66) | 497.98(492.77,503.24) | 229.01(224.77,233.31) | -0.72(-1.78,0.35) |
| Portugal | 2.04(1.50,2.75) | 3.46(2.35,4.94) | 2.73(1.21,4.28) | 6.88(5.69,8.27) | 1.14(0.61,1.99) | -3.75(-4.64,-2.85) | 184.75(178.24,191.47) | 89.29(83.94,94.92) | -0.29(-1.30,0.72) |
| Puerto Rico | 7.48(4.34,12.07) | 6.21(1.62,16.39) | -0.33(-2.87,2.27) | 17.18(12.77,22.81) | 4.60(1.03,13.58) | -3.24(-4.67,-1.79) | 786.97(753.96,821.18) | 739.03(675.73,806.90) | 0.78(-0.67,2.25) |
| Qatar | 5.43(0.74,19.58) | 3.75(1.36,8.70) | -2.00(-3.27,-0.71) | 10.16(3.55,24.89) | 1.96(0.38,6.31) | -6.83(-7.75,-5.90) | 599.16(537.78,666.88) | 398.62(366.96,432.48) | -3.32(-4.40,-2.24) |
| Republic of Korea | 3.17(2.52,3.96) | 2.80(1.86,4.09) | -0.91(-2.43,0.63) | 5.60(4.69,6.66) | 0.48(0.14,1.20) | -7.59(-8.79,-6.37) | 237.21(231.00,243.56) | 99.06(92.80,105.66) | -2.92(-4.17,-1.65) |
| Republic of Moldova | 1.62(0.54,3.97) | 1.53(0.42,5.44) | 0.72(-0.85,2.31) | 14.51(10.44,19.73) | 2.26(0.57,6.85) | -3.58(-4.75,-2.40) | 797.89(766.56,830.26) | 402.28(371.62,435.17) | -0.73(-1.71,0.26) |
| Romania | 2.79(2.26,3.50) | 2.51(1.91,3.25) | -0.01(-2.27,2.31) | 77.15(72.83,81.68) | 13.51(12.03,15.14) | -4.47(-5.36,-3.56) | 927.74(912.25,943.45) | 260.23(253.67,266.94) | -2.82(-3.58,-2.06) |
| Russian Federation | 4.08(3.66,4.55) | 2.81(2.53,3.13) | -4.82(-6.40,-3.22) | 1.30(1.07,1.58) | 0.28(0.16,0.45) | -7.21(-8.30,-6.12) | 812.08(805.73,818.48) | 414.99(410.26,419.76) | -4.64(-5.82,-3.44) |
| Rwanda | 17.40(15.14,19.95) | 10.36(8.79,12.17) | -2.19(-4.25,-0.08) | 211.84(203.75,220.20) | 79.80(75.10,84.75) | -4.03(-4.71,-3.34) | 1181.64(1161.99,1201.57) | 820.12(804.70,835.79) | -1.65(-2.28,-1.01) |
| Saint Kitts and Nevis | 4.46(0.00,116.89) | 5.04(0.00,211.34) | -0.87(-2.78,1.08) | 58.46(11.42,198.85) | 29.87(0.20,251.63) | -2.67(-3.85,-1.47) | 1348.30(1033.53,1739.90) | 869.17(533.82,1355.08) | -1.72(-2.68,-0.75) |
| Saint Lucia | 2.41(0.02,32.06) | 4.66(0.00,78.53) | -1.83(-3.21,-0.43) | 68.66(36.47,122.10) | 38.32(6.67,130.14) | -1.93(-3.31,-0.52) | 1183.56(1027.71,1358.92) | 1016.34(788.36,1294.51) | -0.17(-1.38,1.07) |
| Saint Vincent and the Grenadines | 17.94(1.42,77.55) | 5.12(0.00,103.87) | -3.27(-5.33,-1.16) | 103.97(53.49,188.82) | 68.91(14.88,205.76) | -2.61(-3.57,-1.65) | 1377.03(1162.84,1622.99) | 1357.15(1047.90,1735.51) | -0.63(-1.32,0.07) |
| Samoa | 0.26(0.00,19.60) | 0.37(0.00,19.89) | 0.17(-1.38,1.73) | 138.74(98.37,192.40) | 70.22(44.29,109.09) | -2.58(-3.61,-1.54) | 977.84(857.51,1111.60) | 622.21(537.22,719.15) | -1.46(-2.41,-0.50) |
| San Marino | 8.04(0.00,501.90) | 3.35(0.00,448.74) | -0.14(-1.65,1.39) | 1.24(0.00,487.53) | 0.47(0.00,445.75) | -3.70(-5.12,-2.27) | 164.41(18.69,730.89) | 149.45(12.69,685.24) | -1.06(-2.40,0.30) |
| Sao Tome and Principe | 11.87(1.54,46.91) | 7.10(1.49,29.72) | -3.19(-4.50,-1.85) | 508.72(405.12,632.66) | 127.16(87.29,181.58) | -5.50(-6.36,-4.62) | 1719.52(1525.36,1933.40) | 1317.72(1184.65,1463.93) | -1.47(-2.54,-0.39) |
| Saudi Arabia | 6.62(5.46,7.95) | 1.65(1.37,1.97) | -3.96(-5.19,-2.71) | 8.04(7.02,9.20) | 0.57(0.40,0.79) | -8.31(-9.63,-6.98) | 799.54(788.54,810.69) | 277.90(273.86,281.99) | -3.63(-4.82,-2.43) |
| Senegal | 11.17(10.06,12.37) | 10.19(9.20,11.29) | -0.41(-1.37,0.56) | 357.53(350.99,364.17) | 96.22(92.48,100.09) | -4.11(-5.03,-3.18) | 1864.81(1849.81,1879.90) | 2220.75(2202.68,2238.95) | 0.17(-0.71,1.07) |
| Serbia | 1.73(1.07,2.87) | 1.82(0.58,4.57) | -0.83(-3.76,2.19) | 140.62(131.88,149.84) | 53.95(45.12,64.12) | -4.39(-5.25,-3.53) | 703.26(683.44,723.57) | 524.94(497.07,554.08) | -1.46(-2.40,-0.51) |
| Seychelles | 3.02(0.00,66.35) | 0.51(0.00,22.46) | -9.42(-11.39,-7.40) | 50.48(13.74,139.16) | 7.70(0.61,34.50) | -5.76(-6.54,-4.97) | 786.92(599.35,1019.59) | 317.79(244.93,406.72) | -2.81(-3.53,-2.09) |
| Sierra Leone | 15.87(12.99,19.22) | 6.52(5.69,7.46) | -4.02(-4.89,-3.13) | 358.60(346.42,371.16) | 138.83(134.77,142.98) | -3.56(-4.51,-2.60) | 773.96(755.70,792.60) | 901.90(891.55,912.34) | -0.10(-1.15,0.96) |
| Singapore | 3.59(1.33,7.89) | 1.10(0.44,2.33) | -2.27(-3.95,-0.56) | 3.34(1.40,7.09) | 0.26(0.03,1.08) | -7.68(-8.27,-7.09) | 88.65(76.68,102.18) | 28.93(25.16,33.17) | -3.06(-3.69,-2.42) |
| Slovakia | 2.11(1.18,3.88) | 1.81(0.50,4.94) | -0.15(-1.41,1.13) | 60.61(53.19,68.90) | 16.75(11.62,23.51) | -4.39(-5.67,-3.09) | 705.88(679.67,732.93) | 405.34(378.79,433.40) | -1.71(-2.89,-0.52) |
| Slovenia | 2.11(0.63,7.09) | 3.57(0.54,12.22) | -0.68(-2.26,0.92) | 32.99(23.26,45.90) | 8.94(3.67,19.05) | -4.04(-5.30,-2.76) | 587.60(543.54,634.65) | 327.75(288.92,370.82) | -1.70(-2.92,-0.47) |
| Solomon Islands | 0.54(0.01,3.94) | 0.83(0.01,7.54) | 0.45(-0.64,1.54) | 194.24(171.88,218.82) | 243.47(209.15,282.20) | 0.35(-0.55,1.26) | 509.50(472.86,548.33) | 858.41(795.89,925.03) | 1.85(1.01,2.69) |
| Somalia | 54.25(51.42,57.24) | 68.19(65.57,70.90) | -0.01(-1.03,1.02) | 636.84(625.35,648.52) | 690.83(682.01,699.74) | -0.53(-1.17,0.11) | 1041.38(1025.87,1057.08) | 1165.93(1154.75,1177.20) | 0.05(-0.63,0.73) |
| South Africa | 4.08(3.75,4.44) | 3.87(3.46,4.33) | -0.09(-1.45,1.29) | 87.50(85.84,89.19) | 53.37(51.33,55.49) | -1.50(-2.63,-0.35) | 1152.31(1146.27,1158.37) | 1957.63(1945.16,1970.18) | 2.10(0.96,3.26) |
| South Sudan | 8.12(6.92,9.48) | 7.51(6.70,8.39) | -1.10(-2.04,-0.14) | 266.34(259.76,273.05) | 165.94(161.84,170.12) | -1.89(-2.83,-0.94) | 628.69(618.94,638.58) | 578.53(570.86,586.29) | -0.30(-1.50,0.91) |
| Spain | 10.20(8.54,12.08) | 4.32(3.28,5.62) | -2.37(-4.41,-0.29) | 3.63(2.78,4.68) | 1.19(0.65,2.02) | -4.42(-5.68,-3.13) | 227.77(220.77,234.96) | 228.44(219.98,237.16) | -1.29(-2.38,-0.18) |
| Sri Lanka | 12.75(11.56,14.08) | 1.84(1.18,2.80) | -9.89(-12.07,-7.66) | 152.13(146.32,158.13) | 24.81(21.94,27.98) | -7.98(-9.05,-6.90) | 925.19(910.39,940.19) | 525.44(512.53,538.63) | -2.82(-3.74,-1.89) |
| Sudan | 30.44(29.26,31.65) | 47.58(45.43,49.79) | -0.39(-1.60,0.84) | 148.95(146.25,151.69) | 61.49(59.44,63.61) | -3.63(-4.48,-2.78) | 1082.99(1075.71,1090.31) | 1123.01(1113.78,1132.31) | -0.68(-1.56,0.20) |
| Suriname | 8.56(1.86,26.91) | 3.99(0.24,20.59) | -2.10(-3.84,-0.34) | 130.44(93.81,177.48) | 68.51(42.35,105.67) | -1.86(-3.42,-0.27) | 1462.35(1336.73,1597.50) | 1195.95(1079.56,1322.29) | -0.39(-1.76,1.00) |
| Sweden | 2.36(1.52,3.73) | 4.03(2.41,6.46) | 0.24(-0.77,1.25) | 2.29(1.22,3.99) | 0.61(0.09,2.10) | -3.83(-5.12,-2.51) | 215.92(203.90,228.51) | 170.41(157.80,183.80) | -0.37(-1.56,0.83) |
| Switzerland | 4.58(2.45,7.87) | 1.12(0.63,1.90) | -2.32(-3.95,-0.67) | 0.92(0.27,2.67) | 0.13(0.01,0.60) | -6.26(-7.60,-4.90) | 144.84(133.99,156.52) | 67.40(62.70,72.36) | -2.59(-3.95,-1.21) |
| Syrian Arab Republic | 15.89(14.86,16.97) | 69.05(62.87,75.68) | 3.16(1.86,4.48) | 45.57(43.76,47.44) | 22.46(19.46,25.85) | -2.98(-3.85,-2.11) | 667.40(660.48,674.38) | 898.23(877.82,919.04) | 0.24(-0.44,0.92) |
| Taiwan (Province of China) | 0.77(0.37,1.46) | 0.20(0.08,0.47) | -2.79(-5.01,-0.52) | 11.17(9.32,13.29) | 0.44(0.22,0.81) | -8.81(-10.01,-7.58) | 348.81(338.42,359.46) | 105.77(101.69,109.97) | -2.80(-4.04,-1.55) |
| Tajikistan | 7.98(6.61,9.63) | 15.64(13.27,18.34) | 0.13(-1.27,1.54) | 102.72(96.28,109.51) | 55.69(51.79,59.85) | -1.21(-2.31,-0.09) | 1766.85(1740.21,1793.83) | 1327.38(1307.91,1347.10) | -0.24(-1.06,0.58) |
| Thailand | 13.24(12.05,14.53) | 2.30(1.93,2.72) | -4.83(-7.54,-2.05) | 89.12(86.60,91.71) | 9.72(9.03,10.45) | -6.83(-8.07,-5.57) | 649.82(642.43,657.29) | 231.96(228.49,235.46) | -3.58(-4.64,-2.51) |
| Timor-Leste | 5.18(2.08,11.24) | 1.67(0.62,3.64) | -3.54(-5.97,-1.05) | 277.48(250.98,306.39) | 59.35(52.82,66.58) | -3.31(-4.19,-2.42) | 391.82(361.17,424.80) | 286.11(271.01,301.92) | -0.02(-0.81,0.77) |
| Togo | 40.36(35.21,46.07) | 11.25(9.95,12.68) | -3.83(-5.11,-2.54) | 450.77(435.64,466.37) | 133.79(129.54,138.16) | -2.45(-3.03,-1.87) | 1338.34(1313.38,1363.74) | 1638.00(1623.20,1652.90) | 2.16(1.26,3.07) |
| Tokelau | 1.88(0.00,3337.44) | 1.31(0.00,7093.12) | 0.20(-1.87,2.31) | 218.33(0.00,3727.75) | 88.33(0.00,7255.98) | -1.29(-2.80,0.24) | 1198.21(106.30,5295.84) | 1050.19(5.61,8904.00) | 1.05(-0.28,2.39) |
| Tonga | 0.73(0.00,32.84) | 1.05(0.00,41.54) | 1.61(0.68,2.55) | 113.31(65.94,184.71) | 42.73(16.51,98.81) | -2.19(-2.89,-1.49) | 816.67(677.38,978.48) | 553.94(440.26,693.07) | -0.60(-1.26,0.07) |
| Trinidad and Tobago | 5.93(3.49,9.48) | 2.95(0.98,6.90) | -5.25(-7.46,-2.98) | 25.28(20.33,31.19) | 13.83(9.29,20.05) | -3.25(-4.32,-2.17) | 661.14(634.35,688.87) | 460.66(432.00,490.88) | -2.48(-3.40,-1.55) |
| Tunisia | 12.96(10.64,15.67) | 3.60(2.93,4.38) | -2.52(-3.83,-1.20) | 53.29(48.41,58.56) | 10.65(9.39,12.04) | -4.26(-4.85,-3.68) | 1203.18(1179.38,1227.38) | 349.72(342.23,357.34) | -3.45(-4.07,-2.84) |
| Türkiye | 69.82(67.51,72.19) | 11.39(10.77,12.06) | -1.94(-3.56,-0.30) | 66.03(64.11,67.99) | 11.83(10.87,12.86) | -6.41(-6.89,-5.94) | 958.84(951.37,966.37) | 544.37(537.75,551.06) | -2.42(-2.72,-2.12) |
| Turkmenistan | 1.95(1.20,3.24) | 1.59(0.97,2.49) | -0.06(-1.63,1.53) | 43.79(38.57,49.58) | 18.50(16.15,21.12) | -1.83(-2.65,-0.99) | 1713.56(1680.07,1747.61) | 743.97(728.83,759.37) | -1.78(-2.52,-1.04) |
| Tuvalu | 1.41(0.00,371.79) | 0.44(0.00,162.80) | -2.62(-4.02,-1.21) | 247.46(55.22,752.04) | 65.48(7.38,267.55) | -2.17(-3.22,-1.11) | 950.80(511.45,1663.34) | 513.68(292.72,854.80) | -0.56(-1.36,0.25) |
| Uganda | 12.23(11.11,13.45) | 2.65(2.41,2.91) | -3.87(-5.27,-2.44) | 284.21(278.57,289.94) | 50.80(49.69,51.92) | -4.13(-5.06,-3.20) | 1158.46(1147.14,1169.88) | 458.26(455.04,461.49) | -1.86(-2.62,-1.10) |
| Ukraine | 7.12(6.47,7.84) | 32.16(28.63,36.02) | -1.74(-4.64,1.25) | 6.45(5.60,7.41) | 2.52(1.68,3.66) | -4.20(-5.55,-2.83) | 633.59(625.07,642.21) | 755.61(739.23,772.28) | -1.69(-3.24,-0.11) |
| United Arab Emirates | 14.02(9.07,20.91) | 7.61(5.72,10.26) | -2.40(-3.96,-0.82) | 17.65(12.06,25.17) | 3.29(1.71,5.88) | -4.61(-5.22,-4.00) | 761.94(722.20,803.46) | 646.50(620.30,673.60) | 0.00(-0.57,0.57) |
| United Kingdom | 5.97(5.09,6.98) | 5.00(4.23,5.89) | -0.27(-1.63,1.11) | 2.61(2.17,3.14) | 1.40(0.96,1.97) | -2.48(-3.91,-1.03) | 209.78(205.66,213.98) | 336.90(329.67,344.26) | 0.77(-0.61,2.18) |
| United Republic of Tanzania | 9.85(8.90,10.89) | 2.83(2.62,3.05) | -1.92(-3.20,-0.62) | 264.21(259.82,268.66) | 61.76(60.72,62.82) | -4.20(-5.11,-3.29) | 1834.04(1823.02,1845.11) | 770.55(766.89,774.23) | -3.27(-4.12,-2.41) |
| United States of America | 1.26(1.14,1.39) | 3.52(3.19,3.86) | -0.07(-1.99,1.90) | 0.73(0.62,0.85) | 0.37(0.28,0.48) | -3.39(-4.65,-2.12) | 54.61(53.65,55.59) | 70.81(69.46,72.18) | -0.32(-1.54,0.91) |
| United States Virgin Islands | 1.96(0.01,18.98) | 3.82(0.00,166.84) | -2.77(-4.59,-0.92) | 11.13(2.42,33.47) | 4.70(0.00,167.37) | -4.62(-5.69,-3.54) | 448.20(373.62,534.21) | 717.84(442.28,1114.03) | -0.04(-1.05,0.98) |
| Uruguay | 1.04(0.41,2.27) | 1.91(0.32,6.54) | 0.69(-0.82,2.22) | 22.47(19.00,26.45) | 12.02(6.85,19.88) | -3.75(-4.56,-2.94) | 132.19(123.63,141.25) | 157.99(137.34,181.09) | -0.86(-1.38,-0.35) |
| Uzbekistan | 22.22(20.40,24.17) | 12.95(11.64,14.36) | -5.22(-6.99,-3.42) | 65.46(62.85,68.16) | 31.49(29.75,33.32) | -4.07(-5.11,-3.02) | 1773.96(1759.36,1788.66) | 1473.58(1461.04,1486.21) | -2.55(-3.56,-1.53) |
| Vanuatu | 6.13(0.30,30.56) | 4.88(0.32,22.20) | -1.55(-2.76,-0.32) | 180.39(136.00,237.52) | 118.72(88.53,157.79) | -1.12(-2.19,-0.04) | 585.23(502.98,679.64) | 797.29(714.99,887.99) | 0.56(-0.51,1.63) |
| Venezuela (Bolivarian Republic of) | 1.95(1.63,2.31) | 13.39(11.64,15.34) | 2.25(0.20,4.35) | 19.99(18.90,21.14) | 19.18(17.42,21.11) | -1.97(-3.52,-0.39) | 338.43(333.92,342.99) | 361.00(352.94,369.22) | -1.27(-2.39,-0.14) |
| Viet Nam | 6.48(6.18,6.80) | 2.44(2.19,2.72) | -6.81(-9.06,-4.51) | 42.18(41.34,43.03) | 22.97(21.84,24.15) | -4.77(-6.13,-3.39) | 576.25(573.15,579.36) | 607.16(601.43,612.94) | -3.13(-4.27,-1.98) |
| Yemen | 25.38(24.00,26.84) | 31.06(30.02,32.12) | -1.30(-2.56,-0.03) | 360.76(353.50,368.15) | 78.10(76.52,79.70) | -4.78(-5.28,-4.29) | 3055.06(3035.35,3074.88) | 2262.71(2253.73,2271.73) | -1.46(-2.04,-0.87) |
| Zambia | 14.05(12.20,16.13) | 11.42(10.09,12.89) | -0.53(-1.79,0.73) | 364.36(354.59,374.36) | 157.63(152.54,162.85) | -2.46(-3.22,-1.70) | 2276.57(2251.63,2301.75) | 2680.05(2658.03,2702.21) | 0.96(0.02,1.91) |
| Zimbabwe | 40.12(36.77,43.70) | 19.78(17.62,22.12) | -1.70(-3.59,0.23) | 190.53(184.19,197.07) | 135.21(130.34,140.25) | -0.62(-1.29,0.06) | 997.56(983.05,1012.26) | 1224.77(1209.81,1239.89) | 0.56(0.00,1.12) |

STable 5. Age-standardized incidence rate of nutritional deficiency in 0-14 years at the national level and their changing trends from 1990 to 2021.

|  | 1990 | 2021 |  | 1990 | 2021 |  |
| --- | --- | --- | --- | --- | --- | --- |
|  | Iodine deficiency | Iodine deficiency | Iodine deficiency | Vitamin A deficiency | Vitamin A deficiency | Vitamin A deficiency |
| countries | 1990 No.(95%UI) | 2021 No.(95%UI) | 1990-2021 EAPC No.(95%CI) | 1990 No.(95%UI) | 2021 No.(95%UI) | 1990-2021 EAPC No.(95%CI) |
| Afghanistan | 249.95(241.99,258.12) | 180.87(177.80,183.99) | -1.17(-2.62,0.31) | 78527.21(78393.50,78661.11) | 56334.20(56269.49,56398.97) | -1.07(-1.50,-0.65) |
| Albania | 41.93(36.58,48.03) | 48.45(35.97,64.08) | 1.11(-0.16,2.39) | 59853.93(59604.25,60104.47) | 27962.52(27657.91,28269.92) | -2.14(-3.02,-1.26) |
| Algeria | 90.29(87.69,92.96) | 141.23(137.18,145.37) | -1.18(-2.62,0.27) | 19458.74(19412.16,19505.41) | 6806.52(6778.93,6834.19) | -3.17(-4.11,-2.21) |
| American Samoa | 3.85(0.02,30.82) | 3.52(0.00,158.39) | 0.43(-1.13,2.02) | 15049.23(14473.58,15642.52) | 14237.29(13014.77,15558.34) | -0.32(-1.62,1.00) |
| Andorra | 43.27(4.26,255.82) | 110.15(11.74,450.97) | -0.17(-2.08,1.77) | 3386.95(2706.46,4205.25) | 1502.27(961.39,2252.45) | -4.74(-6.12,-3.34) |
| Angola | 730.45(721.89,739.09) | 977.84(968.72,987.04) | -1.91(-2.92,-0.88) | 49828.75(49760.23,49897.34) | 30638.58(30591.67,30685.56) | -2.99(-4.01,-1.95) |
| Antigua and Barbuda | 38.66(8.47,138.10) | 41.46(3.42,181.91) | 0.12(-0.73,0.98) | 8730.22(8011.03,9504.60) | 4544.97(3918.54,5250.23) | -1.67(-2.24,-1.09) |
| Argentina | 24.05(22.65,25.53) | 48.14(45.16,51.27) | 0.67(-0.81,2.16) | 35015.38(34949.89,35080.98) | 22050.22(21987.90,22112.68) | -1.92(-2.27,-1.57) |
| Armenia | 120.69(113.24,128.52) | 254.48(228.34,282.98) | 1.03(-0.35,2.43) | 941.96(921.66,962.63) | 982.82(931.86,1036.05) | -0.61(-1.59,0.37) |
| Australia | 15.71(14.45,17.06) | 44.76(40.89,48.93) | 2.48(0.90,4.08) | 45.01(42.80,47.31) | 42.89(39.13,46.95) | -0.45(-1.77,0.89) |
| Austria | 106.71(95.85,118.52) | 34.25(31.10,37.66) | -2.87(-4.02,-1.72) | 4096.37(4034.85,4158.69) | 656.71(642.02,671.67) | -5.79(-6.75,-4.83) |
| Azerbaijan | 39.03(35.55,42.84) | 21.17(19.33,23.15) | -3.12(-5.09,-1.12) | 7399.86(7341.52,7458.57) | 2475.39(2454.01,2496.91) | -3.83(-5.53,-2.10) |
| Bahamas | 22.02(11.61,38.49) | 47.30(18.13,101.50) | 0.37(-1.70,2.48) | 3139.34(3001.66,3282.20) | 3453.79(3155.91,3773.28) | -1.26(-2.92,0.42) |
| Bahrain | 115.26(86.59,150.98) | 115.78(90.98,145.70) | -0.62(-0.94,-0.31) | 8562.18(8305.85,8824.99) | 3906.77(3757.14,4061.22) | -2.67(-2.91,-2.42) |
| Bangladesh | 572.44(570.06,574.83) | 401.19(397.21,405.19) | -1.56(-2.87,-0.24) | 20170.51(20157.34,20183.70) | 6806.38(6791.61,6821.17) | -2.86(-3.71,-2.01) |
| Barbados | 42.56(25.61,67.52) | 111.15(54.68,203.64) | -0.37(-1.87,1.16) | 4181.39(3996.21,4373.68) | 5392.73(4951.21,5865.56) | -1.63(-2.73,-0.51) |
| Belarus | 18.19(16.32,20.23) | 43.85(36.94,51.74) | -1.25(-3.53,1.08) | 2221.81(2200.99,2242.78) | 1479.53(1438.54,1521.48) | -4.93(-6.72,-3.11) |
| Belgium | 54.00(49.24,59.22) | 88.53(79.80,98.01) | 2.28(1.15,3.42) | 4466.90(4413.96,4520.38) | 1577.37(1539.51,1615.97) | -2.26(-3.33,-1.18) |
| Belize | 29.41(11.36,63.99) | 20.56(7.17,47.60) | -0.99(-2.40,0.44) | 23571.20(22962.95,24192.88) | 10241.47(9870.57,10623.69) | -2.59(-3.73,-1.43) |
| Benin | 72.06(68.26,76.03) | 109.53(104.86,114.36) | 0.58(-0.47,1.64) | 64088.73(63978.86,64198.75) | 55195.66(55092.95,55298.54) | -0.86(-1.75,0.04) |
| Bermuda | 37.78(1.80,203.57) | 27.14(0.03,298.24) | -0.35(-1.44,0.74) | 5671.32(4899.17,6540.91) | 2543.67(1874.12,3394.93) | -2.35(-2.99,-1.71) |
| Bhutan | 126.15(102.67,153.82) | 44.42(31.84,62.09) | -1.11(-3.02,0.83) | 51955.15(51484.63,52429.40) | 8513.29(8280.24,8751.84) | -6.96(-7.88,-6.03) |
| Bolivia (Plurinational State of) | 17.05(14.33,20.18) | 16.27(13.77,19.13) | -0.96(-2.32,0.41) | 21939.17(21842.02,22036.68) | 13053.48(12982.50,13124.78) | -2.71(-3.60,-1.81) |
| Bosnia and Herzegovina | 105.05(95.91,115.05) | 98.56(80.15,120.11) | 0.02(-1.07,1.11) | 49542.23(49309.26,49776.15) | 24584.72(24289.21,24883.14) | -1.47(-2.50,-0.43) |
| Botswana | 29.08(23.41,36.10) | 33.45(27.46,40.72) | -0.09(-1.47,1.31) | 52673.34(52369.88,52978.29) | 20214.44(20032.41,20397.87) | -3.73(-4.79,-2.65) |
| Brazil | 14.06(13.56,14.57) | 20.77(19.98,21.60) | -1.56(-2.86,-0.25) | 49982.15(49948.26,50016.05) | 20990.54(20966.30,21014.82) | -4.32(-5.23,-3.41) |
| Brunei Darussalam | 26.09(13.38,49.19) | 53.33(27.85,93.92) | 1.80(0.57,3.05) | 7256.69(6969.97,7553.24) | 2968.39(2760.89,3188.75) | -2.33(-3.37,-1.27) |
| Bulgaria | 63.67(55.73,72.49) | 14.05(11.77,16.70) | -4.30(-5.60,-2.99) | 38215.43(38018.19,38413.50) | 8078.55(8018.70,8138.78) | -4.58(-5.57,-3.57) |
| Burkina Faso | 108.92(104.98,112.99) | 105.01(101.61,108.51) | -0.94(-1.90,0.03) | 116793.87(116648.97,116938.93) | 50045.96(49972.78,50119.23) | -2.36(-2.85,-1.88) |
| Burundi | 314.60(305.70,323.75) | 104.30(101.63,107.03) | -3.13(-4.01,-2.24) | 65893.26(65742.36,66044.44) | 17287.62(17251.56,17323.74) | -3.76(-4.73,-2.77) |
| Cabo Verde | 215.98(175.72,263.23) | 86.30(69.00,106.82) | -2.16(-3.67,-0.63) | 61987.53(61319.42,62661.82) | 6646.22(6493.62,6801.74) | -6.39(-7.62,-5.15) |
| Cambodia | 238.24(230.22,246.47) | 67.63(63.06,72.46) | -4.18(-5.81,-2.52) | 63934.99(63820.08,64050.08) | 21224.86(21152.53,21297.41) | -4.39(-5.46,-3.31) |
| Cameroon | 46.38(44.29,48.54) | 90.86(87.80,93.99) | 1.69(0.85,2.53) | 73341.45(73258.68,73424.30) | 41042.23(40978.79,41105.75) | -2.44(-3.18,-1.70) |
| Canada | 46.36(42.97,49.96) | 49.89(46.23,53.79) | 1.33(0.05,2.62) | 8714.66(8667.22,8762.32) | 2688.65(2661.13,2716.40) | -3.67(-5.06,-2.25) |
| Central African Republic | 1003.16(973.37,1033.69) | 603.02(589.01,617.36) | -0.42(-1.30,0.47) | 83887.65(83625.68,84150.31) | 68757.44(68581.12,68934.15) | -0.23(-0.57,0.12) |
| Chad | 249.56(240.33,259.07) | 69.54(67.31,71.84) | -4.46(-5.27,-3.65) | 129335.32(129128.57,129542.35) | 71045.22(70960.95,71129.57) | -2.98(-3.53,-2.43) |
| Chile | 61.88(57.31,66.74) | 45.27(40.56,50.41) | -1.57(-3.11,-0.01) | 26148.92(26054.80,26243.32) | 12544.83(12464.55,12625.54) | -2.54(-3.73,-1.33) |
| China | 78.03(77.68,78.39) | 67.21(66.72,67.71) | -0.35(-2.49,1.84) | 17218.33(17213.38,17223.28) | 6072.34(6066.09,6078.58) | -3.74(-5.14,-2.31) |
| Colombia | 98.64(95.25,102.12) | 64.15(61.87,66.52) | 1.82(0.51,3.14) | 16322.12(16280.11,16364.23) | 4958.06(4934.71,4981.52) | -2.49(-3.32,-1.65) |
| Comoros | 68.32(50.30,91.20) | 75.76(55.71,101.08) | 0.23(-1.03,1.50) | 69121.07(68533.79,69712.65) | 27523.14(27133.96,27916.97) | -3.65(-4.35,-2.94) |
| Congo | 1572.48(1529.14,1616.81) | 1047.84(1026.26,1069.86) | -0.86(-1.93,0.23) | 102459.10(102118.58,102800.58) | 73801.36(73590.68,74012.57) | -1.54(-2.35,-0.72) |
| Cook Islands | 6.91(0.00,245.47) | 3.61(0.00,472.70) | 0.05(-1.39,1.52) | 34081.02(31722.55,36590.20) | 8168.80(6624.38,10019.05) | -3.58(-4.40,-2.75) |
| Costa Rica | 95.05(84.52,106.61) | 46.10(41.35,51.28) | -0.48(-1.74,0.80) | 14615.13(14488.69,14742.51) | 2689.43(2653.20,2726.06) | -3.85(-4.77,-2.92) |
| Croatia | 72.91(61.74,85.61) | 20.69(16.58,25.58) | -3.83(-5.29,-2.33) | 35587.31(35339.87,35836.17) | 7156.37(7078.14,7235.32) | -4.94(-6.01,-3.86) |
| Cuba | 34.45(32.13,36.91) | 70.82(62.42,80.09) | 1.29(0.12,2.47) | 4996.11(4967.12,5025.23) | 5352.21(5279.67,5425.55) | -0.06(-1.19,1.09) |
| Cyprus | 61.40(46.71,80.67) | 82.52(60.67,110.32) | 0.75(-0.80,2.32) | 8393.84(8177.57,8614.99) | 1807.26(1698.05,1922.15) | -3.95(-5.01,-2.89) |
| Czechia | 23.24(20.20,26.73) | 27.58(22.72,33.23) | 1.20(0.42,1.98) | 22356.63(22247.08,22466.66) | 11031.32(10932.18,11131.20) | -1.48(-2.30,-0.65) |
| Côte d'Ivoire | 175.95(170.11,181.96) | 69.58(67.54,71.69) | -2.79(-3.74,-1.83) | 85440.17(85310.78,85569.71) | 30305.69(30253.95,30357.51) | -3.44(-3.68,-3.21) |
| Democratic People's Republic of Korea | 5.18(4.55,5.87) | 10.02(8.20,12.15) | 0.47(-0.98,1.94) | 16705.99(16670.86,16741.18) | 18997.30(18919.23,19075.63) | 0.44(-0.58,1.47) |
| Democratic Republic of the Congo | 2245.99(2234.69,2257.34) | 933.81(930.63,936.99) | -1.61(-2.73,-0.47) | 58554.17(58500.78,58607.60) | 31026.74(31007.84,31045.65) | -1.63(-2.66,-0.59) |
| Denmark | 43.93(38.82,49.55) | 97.13(84.66,110.98) | 3.20(1.85,4.58) | 1474.40(1446.41,1502.85) | 702.24(670.75,734.95) | -1.88(-3.10,-0.64) |
| Djibouti | 601.50(554.47,652.46) | 354.61(336.35,373.69) | -1.45(-2.95,0.07) | 51680.83(51150.44,52215.83) | 12530.34(12415.71,12645.81) | -3.92(-4.49,-3.35) |
| Dominica | 43.97(19.34,88.06) | 38.26(4.14,209.81) | -0.03(-1.45,1.40) | 6865.16(6504.06,7242.77) | 3850.77(3235.26,4568.34) | -1.97(-3.34,-0.58) |
| Dominican Republic | 65.28(61.13,69.69) | 35.36(32.34,38.67) | -1.12(-2.47,0.26) | 26650.90(26552.29,26749.82) | 8196.51(8142.76,8250.56) | -3.37(-4.50,-2.24) |
| Ecuador | 15.52(13.26,18.09) | 16.20(14.01,18.66) | 0.84(-0.73,2.44) | 20048.06(19966.79,20129.60) | 9807.71(9754.64,9861.03) | -2.25(-3.52,-0.95) |
| Egypt | 319.25(315.13,323.40) | 74.80(73.81,75.80) | -4.52(-5.76,-3.26) | 13042.23(13018.20,13066.30) | 2283.52(2278.24,2288.81) | -3.80(-4.90,-2.68) |
| El Salvador | 65.74(61.14,70.68) | 56.16(51.13,61.68) | 0.27(-1.20,1.77) | 36251.05(36118.52,36383.98) | 9925.55(9844.87,10006.80) | -3.94(-4.94,-2.93) |
| Equatorial Guinea | 3043.73(2921.55,3170.07) | 912.31(861.03,966.01) | -4.31(-5.44,-3.17) | 78860.55(78277.11,79447.80) | 14700.82(14498.58,14905.37) | -5.97(-6.56,-5.38) |
| Eritrea | 64.11(58.93,69.72) | 45.73(42.07,49.69) | 1.11(0.09,2.13) | 88254.92(88028.49,88481.84) | 39552.49(39420.21,39685.13) | -1.58(-2.26,-0.88) |
| Estonia | 16.69(10.81,25.55) | 14.14(7.49,26.15) | -1.49(-2.68,-0.29) | 2853.38(2755.53,2954.18) | 518.54(466.86,575.18) | -6.07(-7.19,-4.93) |
| Eswatini | 151.39(130.01,175.49) | 78.78(66.76,92.90) | -2.56(-3.60,-1.52) | 51648.39(51271.60,52027.57) | 21995.39(21747.04,22246.11) | -3.64(-4.63,-2.64) |
| Ethiopia | 657.72(653.68,661.79) | 548.60(544.77,552.46) | 0.78(-0.88,2.47) | 83548.00(83493.62,83602.41) | 29858.34(29829.18,29887.52) | -3.55(-4.36,-2.74) |
| Fiji | 6.32(3.41,10.89) | 7.93(2.65,18.91) | -1.19(-2.27,-0.09) | 22498.48(22299.40,22699.02) | 20799.33(20453.75,21149.65) | -1.94(-2.59,-1.28) |
| Finland | 45.17(39.45,51.77) | 130.51(113.88,148.97) | 1.30(-0.41,3.05) | 3354.99(3291.95,3419.06) | 1338.35(1287.98,1390.35) | -3.99(-5.20,-2.76) |
| France | 45.89(44.14,47.70) | 59.93(57.82,62.11) | 2.30(1.11,3.50) | 812.84(803.73,822.05) | 384.75(378.27,391.34) | -0.96(-1.54,-0.37) |
| Gabon | 430.60(409.74,452.28) | 236.05(222.59,250.15) | -4.50(-6.27,-2.69) | 21096.62(20946.93,21247.15) | 8116.71(8040.17,8193.85) | -3.62(-4.65,-2.58) |
| Gambia | 171.12(158.78,184.21) | 138.66(130.63,147.10) | -1.37(-2.33,-0.41) | 57534.65(57301.16,57768.88) | 26083.86(25972.92,26195.20) | -2.75(-3.53,-1.97) |
| Georgia | 86.52(76.38,97.70) | 26.36(22.72,30.47) | -2.27(-4.11,-0.39) | 5200.35(5122.06,5279.62) | 1826.66(1794.28,1859.51) | -3.05(-4.61,-1.46) |
| Germany | 101.87(98.40,105.43) | 91.48(88.12,94.95) | -0.33(-2.15,1.53) | 1166.15(1155.17,1177.22) | 546.01(537.57,554.56) | -2.67(-3.69,-1.64) |
| Ghana | 402.76(394.25,411.40) | 111.16(108.67,113.71) | -2.59(-3.74,-1.43) | 83814.61(83702.89,83926.44) | 38589.70(38533.23,38646.24) | -2.44(-3.35,-1.52) |
| Greece | 63.36(58.04,69.21) | 58.35(52.49,64.89) | 1.47(0.01,2.95) | 6820.41(6748.91,6892.55) | 2313.65(2268.33,2359.75) | -2.47(-3.52,-1.42) |
| Greenland | 22.68(2.07,119.14) | 15.43(1.48,66.52) | -0.90(-2.53,0.75) | 2787.48(2372.29,3261.86) | 944.37(768.86,1150.21) | -3.10(-4.28,-1.90) |
| Grenada | 50.12(22.11,108.81) | 21.19(4.79,61.87) | -4.51(-5.88,-3.12) | 22960.06(22115.63,23831.99) | 3791.08(3500.92,4100.60) | -5.93(-7.22,-4.63) |
| Guam | 3.92(0.28,17.90) | 2.73(0.06,18.09) | -0.22(-1.61,1.19) | 9964.11(9647.34,10289.01) | 4094.08(3879.07,4318.60) | -2.03(-3.11,-0.94) |
| Guatemala | 58.42(55.21,61.81) | 122.87(116.40,129.62) | 1.79(1.32,2.27) | 30285.74(30195.97,30375.72) | 9189.91(9137.64,9242.43) | -3.60(-3.95,-3.25) |
| Guinea | 588.17(573.29,603.37) | 105.92(103.26,108.64) | -5.21(-5.88,-4.53) | 105309.74(105114.03,105505.76) | 28344.33(28299.12,28389.59) | -2.84(-3.71,-1.96) |
| Guinea-Bissau | 240.73(226.35,255.83) | 283.00(263.48,303.73) | -0.95(-2.40,0.53) | 61305.72(61072.95,61539.18) | 56120.61(55839.67,56402.73) | -1.68(-2.65,-0.69) |
| Guyana | 121.64(100.40,146.28) | 35.00(26.54,45.43) | -2.89(-4.19,-1.57) | 22058.13(21780.54,22338.72) | 4220.18(4125.30,4316.83) | -4.20(-5.21,-3.18) |
| Haiti | 85.93(82.03,89.97) | 123.95(117.82,130.33) | -1.54(-2.89,-0.16) | 32159.32(32086.53,32232.25) | 26564.51(26475.17,26654.09) | -3.05(-4.07,-2.02) |
| Honduras | 130.92(122.13,140.22) | 50.91(48.44,53.47) | -2.11(-3.26,-0.94) | 24363.88(24248.48,24479.74) | 4709.07(4684.11,4734.13) | -4.19(-5.18,-3.19) |
| Hungary | 41.76(38.45,45.30) | 47.57(40.41,55.69) | 1.30(0.36,2.24) | 17533.09(17465.99,17600.40) | 13720.03(13597.79,13843.18) | 0.21(-0.62,1.04) |
| Iceland | 44.85(18.77,91.87) | 21.48(10.38,39.89) | -1.37(-2.47,-0.27) | 3073.18(2836.97,3325.99) | 503.56(445.33,567.95) | -5.87(-6.71,-5.01) |
| India | 1465.02(1462.54,1467.50) | 441.58(440.18,442.99) | -4.02(-5.47,-2.56) | 61073.69(61059.02,61088.37) | 22741.10(22731.18,22751.02) | -3.90(-5.00,-2.78) |
| Indonesia | 50.75(49.98,51.53) | 44.27(43.22,45.35) | 0.53(-0.65,1.73) | 71355.26(71318.95,71391.58) | 15737.58(15719.44,15755.73) | -2.92(-4.06,-1.77) |
| Iran (Islamic Republic of) | 55.23(53.80,56.70) | 34.48(33.14,35.87) | -0.59(-2.18,1.03) | 21638.39(21604.27,21672.56) | 3453.90(3437.90,3469.97) | -4.58(-5.58,-3.56) |
| Iraq | 271.28(264.70,277.99) | 274.07(268.10,280.16) | -1.75(-3.04,-0.44) | 25515.02(25454.78,25575.38) | 8405.54(8373.63,8437.55) | -3.98(-4.62,-3.33) |
| Ireland | 47.92(42.96,53.35) | 125.77(110.72,142.35) | 3.49(2.12,4.88) | 2927.09(2886.94,2967.71) | 1059.46(1018.40,1101.90) | -3.18(-4.27,-2.08) |
| Israel | 110.40(99.95,121.68) | 34.62(32.36,37.00) | -3.47(-4.32,-2.61) | 31021.26(30855.70,31187.56) | 5949.44(5918.29,5980.74) | -4.82(-5.57,-4.07) |
| Italy | 286.83(281.78,291.97) | 472.68(461.24,484.33) | 0.34(-1.86,2.59) | 6655.33(6625.53,6685.25) | 3041.89(3015.11,3068.86) | -2.53(-4.10,-0.94) |
| Jamaica | 46.24(41.05,51.93) | 78.60(63.34,96.55) | 1.73(0.26,3.22) | 7853.59(7786.83,7920.82) | 7417.56(7267.80,7569.80) | -0.07(-1.06,0.93) |
| Japan | 83.90(81.27,86.60) | 31.80(30.41,33.26) | -0.35(-1.88,1.20) | 4048.45(4030.83,4066.14) | 1292.60(1282.24,1303.04) | -2.16(-3.13,-1.17) |
| Jordan | 67.99(63.89,72.30) | 144.95(136.40,153.92) | 2.93(2.22,3.65) | 14578.04(14516.27,14640.02) | 12888.31(12807.14,12969.89) | -0.70(-1.30,-0.08) |
| Kazakhstan | 142.74(136.39,149.32) | 78.08(73.83,82.53) | -2.40(-3.85,-0.94) | 43655.33(43546.97,43763.92) | 16637.92(16578.57,16697.45) | -2.72(-3.84,-1.58) |
| Kenya | 98.83(96.32,101.39) | 207.01(202.72,211.38) | 2.21(0.99,3.43) | 101028.47(100936.96,101120.05) | 60723.95(60654.97,60793.01) | -1.08(-2.06,-0.10) |
| Kiribati | 15.22(1.14,70.43) | 5.23(0.45,22.44) | -4.15(-5.34,-2.95) | 71285.05(69766.83,72831.81) | 33186.59(32582.97,33799.39) | -2.58(-3.63,-1.52) |
| Kuwait | 127.53(108.76,148.81) | 101.95(87.98,117.62) | 0.98(-0.41,2.39) | 5771.53(5643.49,5901.94) | 1458.66(1406.45,1512.45) | -2.79(-3.61,-1.96) |
| Kyrgyzstan | 66.91(59.68,74.83) | 36.65(33.03,40.64) | -0.80(-1.99,0.41) | 11084.33(10994.14,11175.14) | 8152.67(8088.87,8216.89) | -0.02(-1.13,1.11) |
| Lao People's Democratic Republic | 42.47(38.63,46.69) | 25.37(21.79,29.45) | -2.75(-3.97,-1.51) | 84699.02(84491.54,84906.93) | 28842.96(28717.90,28968.48) | -3.68(-4.63,-2.72) |
| Latvia | 16.12(11.53,22.43) | 27.55(16.19,44.24) | -1.72(-3.52,0.12) | 2721.75(2647.50,2797.77) | 822.14(755.00,894.05) | -5.70(-7.08,-4.29) |
| Lebanon | 591.53(565.75,618.27) | 107.57(101.86,113.55) | -5.33(-6.73,-3.92) | 10047.55(9945.41,10150.59) | 1707.48(1683.39,1731.85) | -5.99(-6.43,-5.54) |
| Lesotho | 1595.31(1552.45,1639.30) | 247.37(229.77,266.34) | -1.46(-3.72,0.84) | 83734.78(83372.32,84098.56) | 38235.68(37961.32,38511.70) | -1.74(-2.74,-0.73) |
| Liberia | 44.47(40.41,48.83) | 59.07(54.55,63.96) | -2.17(-3.49,-0.83) | 43878.16(43748.80,44007.82) | 33387.66(33257.51,33518.24) | -3.37(-4.31,-2.41) |
| Libya | 195.03(182.45,208.30) | 125.42(118.59,132.55) | 0.00(-1.13,1.14) | 12611.87(12513.93,12710.44) | 2488.57(2459.09,2518.33) | -3.52(-4.38,-2.66) |
| Lithuania | 26.15(19.58,34.35) | 27.37(17.52,41.12) | -1.60(-3.02,-0.17) | 3262.38(3186.95,3339.29) | 634.86(584.04,689.24) | -6.24(-7.09,-5.38) |
| Luxembourg | 49.95(29.03,84.83) | 80.33(49.20,125.47) | -1.06(-2.35,0.26) | 2979.96(2766.32,3207.38) | 958.41(840.72,1089.15) | -5.27(-6.17,-4.37) |
| Madagascar | 157.72(151.90,163.72) | 106.04(103.96,108.15) | 0.12(-1.33,1.58) | 53391.35(53296.53,53486.33) | 19573.63(19546.15,19601.14) | -2.18(-3.18,-1.17) |
| Malawi | 152.33(147.82,156.97) | 129.69(126.30,133.19) | 0.53(-0.35,1.42) | 78305.07(78186.79,78423.51) | 33639.06(33569.33,33708.91) | -1.44(-2.23,-0.64) |
| Malaysia | 156.04(153.01,159.13) | 33.03(31.48,34.64) | -4.85(-6.54,-3.13) | 5733.69(5714.05,5753.39) | 782.38(775.43,789.37) | -4.63(-5.66,-3.59) |
| Maldives | 94.76(64.03,135.89) | 9.42(4.40,18.39) | -7.86(-9.00,-6.71) | 59115.97(58372.54,59867.76) | 3908.67(3778.78,4042.18) | -8.34(-8.81,-7.87) |
| Mali | 127.61(122.05,133.38) | 95.40(92.33,98.57) | -2.35(-3.44,-1.24) | 120802.36(120632.41,120972.50) | 63317.84(63239.97,63395.79) | -2.70(-3.45,-1.94) |
| Malta | 51.18(31.94,82.29) | 41.05(25.27,63.84) | -3.55(-4.97,-2.11) | 7763.42(7435.10,8103.91) | 1220.99(1126.41,1321.98) | -5.58(-6.35,-4.82) |
| Marshall Islands | 7.74(0.40,40.34) | 5.77(0.00,98.38) | -2.02(-3.66,-0.34) | 51792.66(50722.16,52881.72) | 48259.98(46502.03,50075.33) | -0.87(-1.92,0.18) |
| Mauritania | 288.92(274.55,304.02) | 363.13(346.35,380.55) | -1.06(-1.99,-0.12) | 68209.50(67949.08,68470.75) | 22031.33(21909.22,22154.00) | -2.65(-3.64,-1.64) |
| Mauritius | 39.47(32.11,48.21) | 22.45(14.06,36.01) | -3.65(-5.14,-2.13) | 12325.36(12187.46,12464.58) | 4749.30(4583.18,4920.59) | -3.63(-4.24,-3.01) |
| Mexico | 152.66(150.18,155.17) | 77.74(76.61,78.89) | -0.28(-1.80,1.26) | 40489.81(40452.55,40527.11) | 8639.39(8627.96,8650.83) | -3.14(-4.48,-1.79) |
| Micronesia (Federated States of) | 8.73(1.01,44.82) | 5.76(0.05,63.14) | -3.49(-5.19,-1.76) | 85663.53(84247.52,87100.28) | 71759.70(70102.08,73451.74) | -2.19(-3.38,-1.00) |
| Monaco | 50.24(0.91,453.31) | 32.13(2.52,155.05) | -3.66(-4.64,-2.66) | 1354.40(804.82,2183.65) | 278.21(144.90,492.65) | -5.76(-6.33,-5.19) |
| Mongolia | 73.08(67.37,79.15) | 31.78(28.22,35.69) | -4.54(-6.50,-2.54) | 7035.26(6976.65,7094.24) | 1799.77(1773.03,1826.83) | -4.53(-5.59,-3.46) |
| Montenegro | 37.64(24.53,57.42) | 51.14(27.81,87.40) | -1.52(-2.53,-0.50) | 23578.81(23165.74,23998.33) | 14272.02(13837.00,14718.25) | -3.32(-4.11,-2.53) |
| Morocco | 261.09(257.79,264.43) | 348.68(341.38,356.11) | 0.34(-0.64,1.33) | 23360.57(23328.36,23392.81) | 15732.64(15685.03,15780.36) | -1.72(-2.70,-0.73) |
| Mozambique | 311.69(304.21,319.32) | 122.60(119.18,126.10) | -2.22(-3.87,-0.55) | 106262.36(106126.96,106397.90) | 50671.48(50603.67,50739.37) | -2.06(-2.81,-1.32) |
| Myanmar | 64.24(62.48,66.04) | 35.47(33.76,37.26) | -7.58(-9.35,-5.78) | 79847.44(79769.48,79925.46) | 18384.21(18342.93,18425.56) | -7.47(-8.40,-6.53) |
| Namibia | 54.39(48.42,60.92) | 101.88(88.78,116.46) | -0.81(-1.91,0.30) | 21904.17(21778.55,22030.35) | 17200.96(17035.47,17367.81) | -2.38(-3.08,-1.68) |
| Nauru | 9.25(0.00,376.28) | 4.58(0.00,364.59) | -4.08(-4.87,-3.28) | 40708.86(37327.34,44337.77) | 25955.58(23442.17,28699.42) | -2.66(-3.62,-1.69) |
| Nepal | 111.56(108.63,114.57) | 70.60(68.66,72.58) | -0.07(-1.80,1.69) | 47845.46(47770.89,47920.13) | 5543.63(5526.65,5560.66) | -5.66(-6.54,-4.77) |
| Netherlands | 51.43(47.68,55.47) | 78.48(71.89,85.55) | 2.15(0.81,3.51) | 2732.38(2699.30,2765.81) | 868.00(845.44,891.05) | -2.75(-3.64,-1.85) |
| New Zealand | 26.88(22.01,32.79) | 51.21(42.27,61.56) | 1.28(-0.07,2.66) | 1161.22(1124.32,1199.19) | 790.49(755.26,827.05) | -0.67(-1.74,0.42) |
| Nicaragua | 53.11(49.70,56.70) | 135.39(124.85,146.64) | -0.05(-1.92,1.87) | 11776.60(11723.08,11830.31) | 3666.94(3611.99,3722.57) | -6.15(-7.62,-4.66) |
| Niger | 392.81(383.29,402.53) | 162.81(159.88,165.79) | -3.10(-4.30,-1.88) | 120889.44(120731.34,121047.72) | 98860.25(98776.58,98943.97) | -0.78(-1.62,0.08) |
| Nigeria | 150.46(148.47,152.47) | 150.67(149.28,152.07) | -1.29(-2.64,0.07) | 26054.14(26030.95,26077.35) | 10019.01(10008.62,10029.41) | -3.79(-4.40,-3.18) |
| Niue | 14.92(0.00,2536.52) | 9.30(0.00,5490.88) | -2.88(-4.67,-1.04) | 39361.84(30989.57,49502.14) | 21533.32(12820.70,34391.09) | -2.37(-3.81,-0.91) |
| North Macedonia | 66.63(52.72,83.29) | 21.37(15.84,28.34) | -3.78(-5.15,-2.38) | 65151.71(64699.72,65606.25) | 14307.56(14156.81,14459.65) | -4.80(-5.99,-3.60) |
| Northern Mariana Islands | 7.86(0.00,121.36) | 11.56(0.00,217.79) | 0.65(-0.84,2.16) | 13705.25(12650.72,14832.22) | 16313.75(14713.26,18052.25) | 1.13(0.10,2.16) |
| Norway | 110.83(97.21,125.91) | 35.70(31.94,39.84) | -1.45(-2.85,-0.03) | 2679.24(2617.11,2742.64) | 256.91(245.94,268.29) | -5.81(-6.45,-5.16) |
| Oman | 98.46(87.25,110.82) | 55.40(49.27,62.27) | -1.51(-2.85,-0.15) | 17537.90(17394.21,17682.63) | 2619.61(2569.21,2670.84) | -6.28(-7.41,-5.13) |
| Pakistan | 251.30(249.69,252.90) | 615.74(612.37,619.13) | -0.55(-2.47,1.41) | 21585.35(21571.89,21598.83) | 5787.03(5777.91,5796.16) | -5.22(-6.24,-4.18) |
| Palau | 13.41(0.00,411.04) | 5.30(0.00,576.90) | -2.88(-4.49,-1.25) | 24667.58(21973.00,27628.07) | 12812.04(10684.44,15305.62) | -2.11(-3.47,-0.73) |
| Palestine | 110.81(99.97,122.60) | 105.85(96.26,116.17) | -1.77(-3.13,-0.39) | 32968.05(32786.65,33150.31) | 10333.92(10240.00,10428.56) | -4.56(-5.46,-3.66) |
| Panama | 51.20(44.29,59.14) | 67.94(58.32,78.77) | 0.79(0.17,1.42) | 12034.18(11908.61,12160.89) | 5574.54(5488.97,5661.21) | -2.51(-2.96,-2.05) |
| Papua New Guinea | 8.11(6.66,9.81) | 4.43(3.60,5.45) | -3.70(-4.83,-2.55) | 16969.36(16903.83,17035.11) | 17859.54(17791.56,17927.74) | -0.16(-1.00,0.68) |
| Paraguay | 12.49(10.63,14.59) | 25.01(20.73,29.97) | 1.77(0.77,2.79) | 21035.90(20959.07,21112.95) | 16721.18(16605.89,16837.13) | -0.99(-2.04,0.06) |
| Peru | 13.97(12.47,15.61) | 6.57(5.86,7.37) | -3.05(-4.29,-1.78) | 26388.29(26323.66,26453.05) | 8981.11(8949.46,9012.85) | -5.07(-6.12,-4.01) |
| Philippines | 110.90(109.14,112.69) | 80.61(79.44,81.79) | -2.40(-4.30,-0.47) | 40851.55(40809.74,40893.38) | 11160.38(11148.10,11172.67) | -3.00(-3.96,-2.03) |
| Poland | 24.80(23.63,26.02) | 19.76(18.47,21.12) | 1.72(0.68,2.76) | 18945.45(18913.32,18977.62) | 5646.53(5625.01,5668.12) | -1.95(-2.96,-0.92) |
| Portugal | 42.95(40.23,45.84) | 64.62(59.53,70.03) | 2.56(1.24,3.90) | 6448.07(6410.06,6486.27) | 2007.79(1980.85,2035.03) | -1.46(-2.42,-0.50) |
| Puerto Rico | 63.31(53.70,74.23) | 51.00(35.01,71.97) | -0.05(-2.07,2.01) | 8375.32(8264.93,8486.93) | 3247.80(3112.00,3388.21) | -1.95(-3.44,-0.44) |
| Qatar | 80.51(56.55,112.08) | 78.12(64.18,94.38) | -1.98(-3.26,-0.68) | 4435.23(4253.95,4623.16) | 1255.47(1198.81,1314.31) | -5.20(-6.08,-4.31) |
| Republic of Korea | 71.76(68.37,75.29) | 65.47(60.35,70.92) | -0.74(-2.31,0.84) | 8307.11(8270.51,8343.83) | 1730.76(1704.18,1757.67) | -5.15(-6.24,-4.04) |
| Republic of Moldova | 17.36(12.87,23.00) | 13.58(9.00,20.44) | 1.15(-0.18,2.50) | 5347.33(5265.44,5430.26) | 1591.81(1530.30,1655.53) | -2.27(-3.36,-1.18) |
| Romania | 59.00(55.82,62.36) | 46.64(43.81,49.62) | -0.03(-1.77,1.74) | 32925.98(32835.99,33016.18) | 8182.70(8145.65,8219.88) | -3.27(-4.30,-2.24) |
| Russian Federation | 50.95(49.34,52.61) | 28.61(27.57,29.70) | -4.28(-5.71,-2.83) | 858.15(851.75,864.58) | 222.61(219.13,226.14) | -6.64(-7.75,-5.52) |
| Rwanda | 384.96(373.60,396.60) | 237.78(229.73,246.06) | -2.13(-3.82,-0.40) | 61860.88(61723.29,61998.74) | 25054.88(24973.46,25136.51) | -3.09(-3.71,-2.46) |
| Saint Kitts and Nevis | 45.46(9.06,172.79) | 38.61(0.44,270.58) | -1.78(-3.34,-0.20) | 11103.41(10181.67,12096.67) | 4125.11(3323.79,5073.10) | -3.59(-4.68,-2.48) |
| Saint Lucia | 23.29(8.18,60.36) | 32.25(3.68,124.16) | -1.46(-2.71,-0.20) | 15147.27(14596.42,15716.29) | 7633.30(6967.81,8348.53) | -2.65(-3.92,-1.36) |
| Saint Vincent and the Grenadines | 135.67(71.86,236.13) | 57.83(10.45,188.69) | -3.25(-4.84,-1.65) | 27566.76(26561.93,28602.83) | 11088.86(10163.86,12080.71) | -3.25(-4.11,-2.38) |
| Samoa | 5.44(0.63,26.72) | 5.96(0.36,29.22) | -0.66(-1.99,0.68) | 49358.47(48542.05,50186.89) | 35228.19(34511.79,35957.12) | -1.46(-2.49,-0.41) |
| San Marino | 130.73(4.28,719.80) | 66.61(2.76,530.12) | 0.00(-1.40,1.42) | 2608.80(1703.48,3869.75) | 811.29(387.64,1566.65) | -3.20(-4.50,-1.88) |
| Sao Tome and Principe | 129.74(80.56,200.22) | 72.32(46.86,111.23) | -3.18(-4.32,-2.02) | 93812.76(92370.08,95274.21) | 25751.90(25126.53,26390.64) | -5.46(-6.36,-4.55) |
| Saudi Arabia | 45.84(42.88,48.96) | 14.96(14.09,15.88) | -3.78(-4.99,-2.54) | 3697.90(3674.01,3721.93) | 323.30(318.97,327.68) | -7.34(-8.58,-6.08) |
| Senegal | 132.01(128.10,136.02) | 115.55(111.86,119.36) | -0.79(-1.73,0.17) | 55299.19(55217.74,55380.74) | 14744.43(14696.69,14792.32) | -4.00(-4.91,-3.08) |
| Serbia | 32.11(28.61,36.04) | 41.51(33.72,50.66) | -0.55(-2.55,1.49) | 63399.66(63216.09,63583.69) | 35281.41(35048.42,35515.66) | -2.64(-3.69,-1.57) |
| Seychelles | 35.01(10.30,108.35) | 11.85(2.19,39.95) | -5.92(-7.56,-4.26) | 15583.92(14750.96,16457.21) | 2244.00(2046.08,2457.12) | -6.50(-7.26,-5.72) |
| Sierra Leone | 138.09(129.66,146.97) | 62.71(60.04,65.47) | -3.51(-4.44,-2.58) | 82539.25(82345.87,82733.02) | 26370.45(26314.11,26426.88) | -4.13(-4.90,-3.35) |
| Singapore | 51.13(41.28,62.75) | 19.08(15.91,22.75) | -1.99(-3.23,-0.74) | 5808.10(5703.95,5913.83) | 769.78(749.41,790.61) | -5.70(-6.47,-4.93) |
| Slovakia | 39.15(34.11,44.93) | 39.88(31.63,49.75) | -0.05(-1.32,1.23) | 28335.06(28174.29,28496.63) | 11423.56(11279.51,11569.11) | -2.80(-3.96,-1.62) |
| Slovenia | 39.30(30.23,51.08) | 50.48(35.39,70.23) | -0.42(-2.02,1.21) | 18288.35(18048.09,18531.42) | 9676.60(9456.05,9901.37) | -2.13(-3.39,-0.85) |
| Solomon Islands | 4.87(1.95,10.25) | 8.17(3.06,18.28) | 0.53(-0.51,1.58) | 55522.40(55133.27,55913.68) | 73448.82(72846.47,74055.32) | 0.62(-0.24,1.50) |
| Somalia | 695.52(684.91,706.29) | 899.63(890.39,908.96) | 0.49(-0.47,1.45) | 135186.33(135018.20,135354.63) | 149432.78(149305.31,149560.33) | -0.19(-0.74,0.37) |
| South Africa | 54.17(52.90,55.46) | 63.56(61.68,65.50) | 1.06(-0.23,2.37) | 25612.91(25584.41,25641.43) | 15424.20(15388.84,15459.64) | -1.60(-2.69,-0.50) |
| South Sudan | 85.26(81.33,89.34) | 76.37(73.70,79.12) | -0.99(-1.98,0.00) | 50391.18(50299.16,50483.34) | 33171.90(33113.79,33230.08) | -1.62(-2.43,-0.80) |
| Spain | 162.28(155.55,169.23) | 99.83(94.35,105.57) | -1.61(-3.23,0.04) | 6918.40(6875.83,6961.18) | 2056.58(2031.31,2082.10) | -3.93(-5.05,-2.80) |
| Sri Lanka | 146.99(142.39,151.74) | 47.62(43.94,51.56) | -6.21(-7.94,-4.44) | 47457.73(47353.63,47562.03) | 10991.56(10929.70,11053.71) | -6.80(-7.79,-5.81) |
| Sudan | 176.20(173.29,179.14) | 272.65(267.73,277.64) | -0.31(-1.44,0.84) | 31953.57(31913.85,31993.33) | 14664.60(14631.11,14698.17) | -3.38(-4.21,-2.55) |
| Suriname | 89.13(60.02,128.53) | 43.88(23.83,75.03) | -2.26(-3.90,-0.60) | 22697.10(22193.55,23210.05) | 9778.85(9439.93,10127.59) | -2.43(-3.86,-0.98) |
| Sweden | 54.56(49.64,59.96) | 91.42(82.52,101.08) | 0.48(-0.33,1.30) | 2858.59(2815.68,2902.04) | 936.45(907.02,966.65) | -2.95(-4.00,-1.90) |
| Switzerland | 73.35(64.12,83.61) | 22.43(19.92,25.19) | -2.11(-3.59,-0.60) | 1248.17(1212.61,1284.63) | 176.18(168.70,183.93) | -5.33(-6.49,-4.16) |
| Syrian Arab Republic | 135.43(132.38,138.53) | 424.15(409.22,439.52) | 2.46(1.66,3.27) | 13481.60(13450.14,13513.12) | 13082.39(13002.08,13163.11) | -1.05(-1.68,-0.42) |
| Taiwan (Province of China) | 17.59(15.29,20.16) | 4.21(3.50,5.04) | -2.92(-4.91,-0.88) | 10996.27(10935.96,11056.84) | 621.92(612.43,631.54) | -7.50(-8.96,-6.00) |
| Tajikistan | 85.89(80.77,91.32) | 110.67(104.57,117.07) | -0.34(-1.71,1.06) | 26677.91(26572.41,26783.76) | 16999.58(16928.17,17071.24) | -0.81(-1.91,0.31) |
| Thailand | 149.77(145.71,153.92) | 24.11(22.89,25.37) | -5.35(-7.02,-3.66) | 41355.42(41296.77,41414.14) | 2207.10(2196.52,2217.72) | -9.01(-10.30,-7.69) |
| Timor-Leste | 57.61(45.28,72.59) | 17.29(13.35,22.07) | -2.87(-4.19,-1.52) | 80102.68(79628.09,80579.68) | 17156.33(17037.12,17276.24) | -2.86(-3.90,-1.81) |
| Togo | 394.44(378.59,410.83) | 108.69(104.65,112.85) | -3.79(-4.79,-2.78) | 83836.39(83618.07,84055.19) | 21324.53(21270.29,21378.89) | -2.83(-3.35,-2.30) |
| Tokelau | 19.46(0.00,3369.52) | 13.09(0.00,7114.27) | 0.23(-1.63,2.12) | 66993.73(53973.84,82419.18) | 38629.80(24856.38,57785.61) | -0.04(-1.61,1.57) |
| Tonga | 7.63(0.71,42.25) | 7.83(0.12,53.12) | 0.42(-0.46,1.30) | 43268.31(42274.11,44282.74) | 27426.12(26517.94,28360.33) | -0.49(-1.14,0.18) |
| Trinidad and Tobago | 59.02(50.85,68.19) | 30.82(23.42,39.95) | -4.78(-6.56,-2.96) | 4852.80(4778.85,4927.70) | 1821.31(1762.95,1881.25) | -4.60(-5.66,-3.53) |
| Tunisia | 129.27(121.34,137.62) | 31.64(29.54,33.86) | -3.33(-4.25,-2.41) | 17192.21(17103.85,17280.95) | 1931.20(1913.85,1948.67) | -6.12(-6.67,-5.57) |
| Türkiye | 486.74(480.88,492.65) | 123.88(121.31,126.50) | -2.68(-3.80,-1.56) | 25160.27(25120.41,25200.17) | 5694.18(5673.34,5715.09) | -4.36(-4.74,-3.97) |
| Turkmenistan | 43.20(38.72,48.18) | 30.59(27.50,33.95) | -0.69(-1.83,0.46) | 8446.60(8371.07,8522.70) | 2786.56(2757.17,2816.22) | -2.18(-3.03,-1.32) |
| Tuvalu | 14.01(0.00,393.72) | 4.10(0.00,169.28) | -2.47(-3.62,-1.30) | 67131.30(62808.05,71700.96) | 22488.38(20804.30,24283.18) | -1.59(-2.56,-0.62) |
| Uganda | 129.57(125.72,133.52) | 54.20(53.06,55.36) | -2.02(-3.06,-0.97) | 51953.67(51878.88,52028.55) | 9967.18(9952.21,9982.18) | -3.78(-4.70,-2.86) |
| Ukraine | 77.12(74.61,79.71) | 229.93(220.62,239.55) | -0.99(-3.35,1.43) | 3939.71(3918.37,3961.15) | 2290.52(2261.78,2319.54) | -3.80(-5.32,-2.25) |
| United Arab Emirates | 318.83(292.11,347.44) | 151.55(141.20,162.65) | -1.96(-3.07,-0.83) | 5989.68(5877.90,6103.21) | 1480.77(1442.57,1519.82) | -3.89(-4.51,-3.25) |
| United Kingdom | 100.26(96.62,104.00) | 107.78(103.91,111.78) | 0.30(-1.04,1.66) | 3650.34(3630.54,3670.24) | 1572.09(1556.72,1587.57) | -2.71(-4.00,-1.41) |
| United Republic of Tanzania | 78.90(76.27,81.60) | 27.01(26.34,27.70) | -2.09(-3.26,-0.90) | 39741.71(39687.31,39796.17) | 10283.85(10270.23,10297.50) | -3.63(-4.58,-2.68) |
| United States of America | 25.04(24.45,25.64) | 50.93(49.73,52.16) | -0.25(-1.82,1.34) | 2121.28(2115.14,2127.44) | 1214.41(1208.90,1219.93) | -3.59(-4.87,-2.30) |
| United States Virgin Islands | 20.27(7.58,45.62) | 31.90(0.42,216.59) | -2.27(-3.82,-0.69) | 3710.33(3490.61,3941.11) | 2409.23(1865.31,3071.12) | -3.86(-5.05,-2.65) |
| Uruguay | 19.78(16.39,23.71) | 42.37(31.59,55.81) | 1.05(-0.05,2.17) | 14952.96(14858.02,15048.41) | 16862.21(16635.33,17091.57) | -1.33(-2.09,-0.56) |
| Uzbekistan | 174.87(169.99,179.88) | 94.06(90.68,97.54) | -5.14(-6.56,-3.69) | 11620.25(11583.71,11656.90) | 4960.57(4937.41,4983.83) | -4.40(-5.41,-3.37) |
| Vanuatu | 59.31(31.36,103.07) | 49.04(27.33,81.72) | -1.40(-2.55,-0.24) | 84729.09(83621.95,85848.82) | 62267.34(61465.18,63078.34) | -0.97(-2.01,0.09) |
| Venezuela (Bolivarian Republic of) | 36.04(34.62,37.52) | 115.20(110.18,120.41) | 1.60(0.01,3.21) | 10096.65(10071.85,10121.50) | 14211.62(14160.01,14263.40) | -0.99(-2.69,0.74) |
| Viet Nam | 74.02(72.98,75.08) | 32.31(31.32,33.34) | -6.02(-7.92,-4.08) | 16163.52(16147.14,16179.90) | 9991.24(9967.80,10014.73) | -4.49(-5.98,-2.97) |
| Yemen | 245.56(240.66,250.55) | 267.05(263.89,270.24) | -1.52(-2.49,-0.55) | 74885.32(74784.42,74986.33) | 15148.89(15126.34,15171.47) | -5.58(-6.07,-5.10) |
| Zambia | 149.37(143.02,155.95) | 126.89(122.21,131.71) | -0.24(-1.37,0.90) | 57891.83(57770.82,58013.06) | 21263.54(21205.22,21322.00) | -2.86(-3.61,-2.10) |
| Zimbabwe | 613.18(600.49,626.09) | 335.16(326.35,344.15) | -1.48(-2.91,-0.03) | 55287.41(55173.90,55401.13) | 37066.87(36981.26,37152.66) | -0.94(-1.44,-0.43) |

STable 5. Top three and bottom three countries of nutritional deficiency in 0-14 years.

| Measure | sex | Top three countries | | | Bottom three countries | | |
| --- | --- | --- | --- | --- | --- | --- | --- |
| 2021 ASR (per 100,000 people) | | | | | | | |
| Age-standardized DALY rate | | | | | | | |
|  | Iodine deficiency | Somalia(68.19) | Syrian Arab Republic(69.05) | Angola(79.97) | Guam(0.14) | American Samoa(0.15) | Congo(0.16) |
|  | Vitamin A deficiency | Mali(423.94) | Niger(509.76) | Somalia(690.83) | Australia(0.01) | Monaco(0.12) | Switzerland(0.13) |
|  | Dietary iron deficiency | Zambia(2680.05) | India(2797.92) | Mali(3712.04) | Singapore(28.93) | Monaco(40.02) | Greenland(42.20) |
| ASIR |  |  |  |  |  |  |  |
|  | Iodine deficiency | Democratic Republic of the Congo(933.81) | Angola(977.84) | Comoros(1047.84) | Guam(2.73) | American Samoa(3.52) | Congo(3.61) |
|  | Vitamin A deficiency | Comoros(73801.36) | Niger(98860.25) | Somalia(149432.78) | Australia(42.89) | Switzerland(176.18) | Russian Federation(222.61) |
| EAPC |  |  |  |  |  |  |  |
| DALY |  |  |  |  |  |  |  |
|  | Iodine deficiency | Denmark(3.55) | Jordan(4.28) | Ireland(4.28) | Myanmar(-9.97) | Sri Lanka(-9.89) | Seychelles(-9.42) |
|  | Vitamin A deficiency | Solomon Islands(0.35) | Kuwait(0.49) | Jamaica(1.09) | Taiwan (Province of China)(-8.81) | Maldives(-8.77) | Saudi Arabia(-8.31) |
|  | Dietary iron deficiency | South Africa(2.10) | Togo(2.16) | Burkina Faso(3.59) | Russian Federation(-4.64) | Peru(-4.22) | Austria(-3.89) |
| Incidence |  |  |  |  |  |  |  |
|  | Iodine deficiency | Jordan(2.93) | Denmark(3.20) | Ireland(3.49) | Maldives(-7.86) | Myanmar(-7.58) | Sri Lanka(-6.21) |
|  | Vitamin A deficiency | Democratic People's Republic of Korea(0.44) | Solomon Islands(0.62) | Northern Mariana Islands(1.13) | Thailand(-9.01) | Maldives(-8.34) | Taiwan (Province of China)(-7.50) |
